# A Stepwise, Nitrosonium-Catalyzed Aerobic Oxidation of Thiols to Disulfides and Thiosulfonates

**DOI:** 10.1021/acs.joc.6c00062

**Published:** 2026-03-20

**Authors:** Mallepalli Shankar, Duen-Ren Hou

**Affiliations:** Department of Chemistry, National Central University, 300 Jhong-Da Rd., Jhong-Li, Taoyuan 320317, Taiwan

## Abstract

A selective and controllable oxidation of thiols to disulfides or thiosulfonates has been developed using molecular oxygen, trifluoroacetic acid, and catalytic NaNO_3_. Both aromatic and aliphatic thiols afford high yields. Sequential oxidation from thiols to disulfides and then to thiosulfonates was observed, with the second step governed by the acid concentration and substrate steric and electronic effects. Acid and oxygen cooperatively enable *in situ* generation and turnover of the active nitrosonium species.

## Graphical abstract

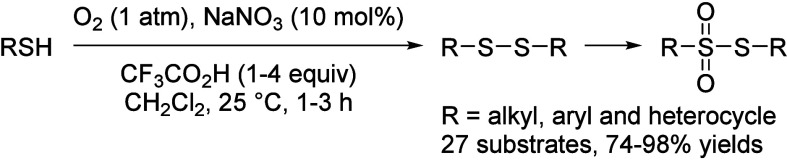

## Introduction

Thiosulfonates, also named as sulfonothioates or the *S*-esters of thiosulfonic acid, are a special class of disulfides, in which one of the sulfur atoms bears two-oxygen atoms (R–SO_2_–S–R′).^1^ Thiosulfonates are increasingly found in natural products and have been recognized for their biological activities, agricultural applications and therapeutic potential (Figure 1).^2,3^ Recently, thiosulfonates have emerged as powerful and convenient reagents for introducing sulfur-containing functional groups.^4–6^ Therefore, the development of efficient methods for their synthesis has attracted considerable attention from chemists.^7^ Oxidation of thiols or disulfides with various oxidants, including chlorine,^8^ *N*-bromosuccinimide (NBS),^9^ ceric ammonium nitrate (CAN),^10^ activated hydrogen peroxide (H_2_O_2_),^11^ potassium peroxymonosulfate (OXONE),^12^ *m*-chloroperbenzoic acid (mCPBA),^13^ Selectfluor,^14^ hypervalent iodines,^15^ HNO_3_/Al­(H_2_PO_4_)_3_ ^16^ and dinitrogen tetroxide (N_2_O_4_),^17^ has been applied to prepare thiosulfonates. Thiosulfonates have also been prepared through self-coupling reactions of sulfinamides,^18^ sulfonyl hydrazides,^19^ and sodium sulfinates^20^ and via reductive coupling reactions of sulfonyl chlorides.^21^ Various cross-coupling reactions, such as those involving sodium thiosulfonates with alkyl halides,^22^ or sulfonic, sulfinic acids and sulfur dioxide (and their derivatives) with diverse sulfur-containing partners,^23–25^ have been developed for the synthesis of unsymmetrical thiosulfonates. Although numerous synthetic strategies have been developed for thiosulfonates, methods that align well with the principles of green chemistry—particularly those demonstrating high atom economy (AE), reaction mass efficiency (RME), and low process mass intensity (PMI)—are still in demand.^7a,26^ In this regard, employing nontoxic molecular oxygen, the greenest and most economical oxidant, is especially appealing.^27^ Representative examples of thiosulfonate synthesis using oxygen or air are shown in Scheme 1.^11d,24c,24d,28^ Early studies employing oxygen as the oxidant or photoassisted conditions generally afforded thiosulfonates in low yields.^28a,28b^ The subsequent introduction of catalysts such as Cu­(I),^24c^ Fe­(III),^24d^ and polyoxomolybdates^11d^ significantly improved reaction efficiency. Nevertheless, alkyl thiosulfonates are typically obtained in lower yields than their aryl counterparts, highlighting the need for further enhancement of the reactivity of aliphatic thiols and for milder, more sustainable reaction conditions, such as low temperatures and metal-free protocols. In this context, a nitrosonium (NO^+^)-catalyzed strategy offers a promising alternative,^29^ and our recent advances in this area are described herein.

**Figure 1:**
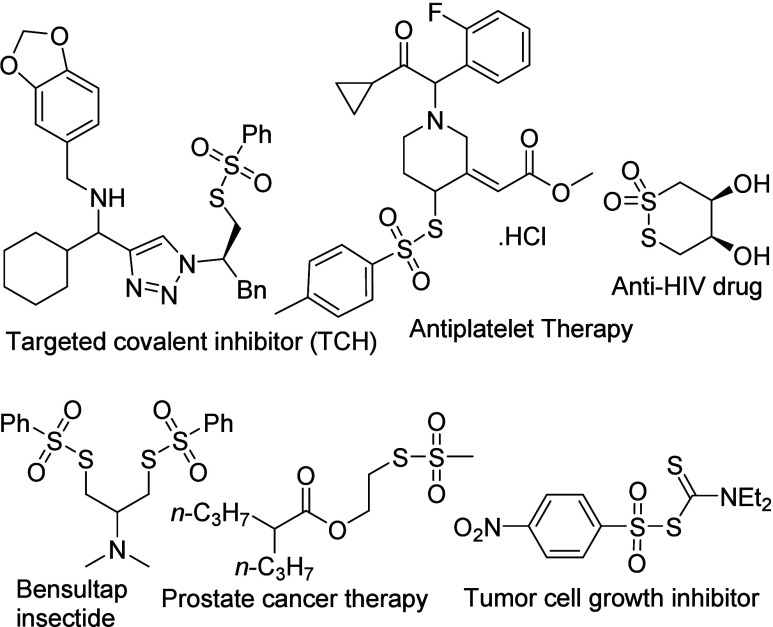
Selected examples of pharmaceutically important thiosulfonates.

**Scheme 1:**
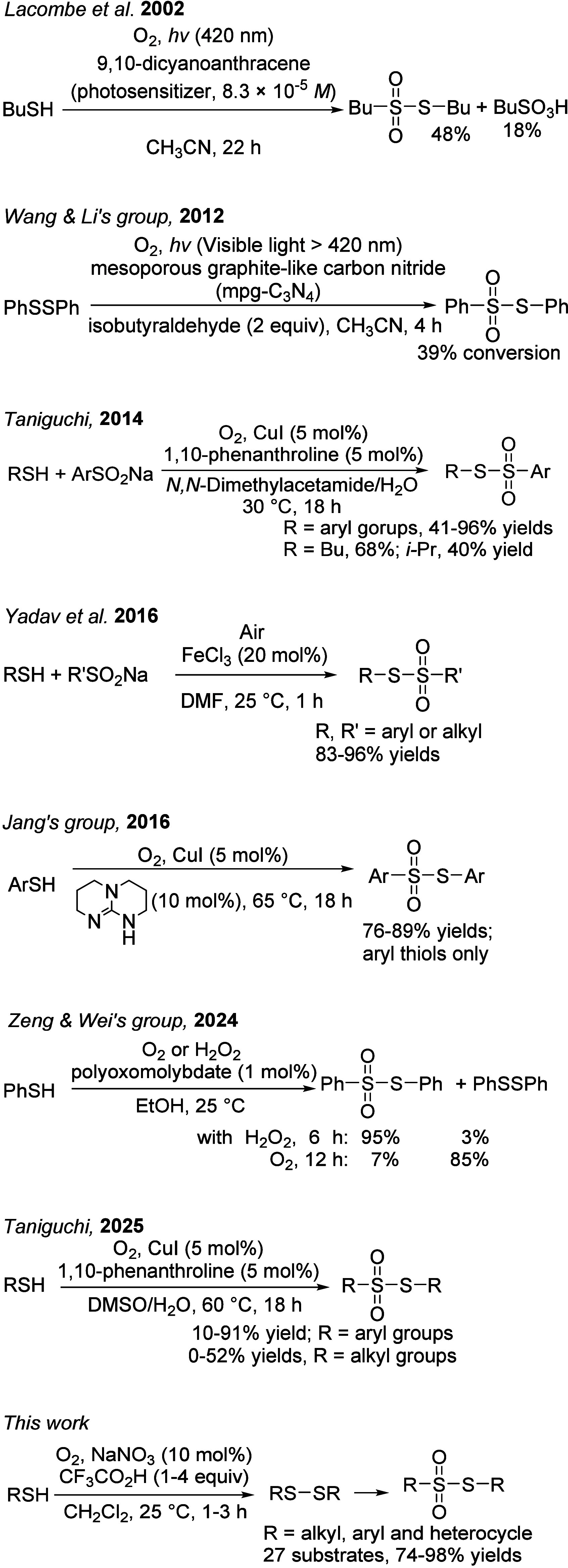
Syntheses of Thiosulfonates under Oxygen

## Results and Discussion

We began our investigation by treating thiophenol (**1a**, 1.0 mmol) with trifluoroacetic acid (TFA, 2.0 mmol) and various nitrosonium (NO^+^) precursors, including nitrosonium tetrafluoroborate (NOBF_4_), *tert*-butyl nitrite (*t*-C_4_H_9_-ONO), sodium nitrite (NaNO_2_), sodium nitrate (NaNO_3_) and nitric acid (HNO_3_), in dichloromethane under an oxygen atmosphere (entries 1–5, Table 1). After 1–2 h at room temperature, all reactions afforded *S*-phenyl benzenesulfonothioate (**2a**) in excellent yields (90% to quantitative). In contrast, thiosulfonate **2a** was not formed when either TFA or the NO^+^ source was omitted or when the reaction was conducted under a nitrogen atmosphere (entries 6–8). Under air, the reaction was slow, yielding 1,2-diphenyl disulfide (**3a**) after 16 h (entry 9). When nitronium tetrafluoroborate (NO_2_BF_4_) was used—a precursor to NO_2_^+^ and expected to generate NO^+^ via equilibrium with TFA^30^ —the yield of **2a** was slightly lower (79%, entry 10) after 2 h. Considering the reagent cost, operational convenience, and need to monitor or isolate reaction intermediates (e.g., disulfides; *vide infra*), sodium nitrate was selected as the preferred precursor for *in situ* generation of NO^+^. The reaction required 2 equiv or more of TFA as the formation of **2a** was suppressed with 1.5 equiv of TFA, but the yield of **2a** did not go higher with 2.5 equiv of the acid (entries 11 and 12). Other organic acids, such as acetic and trichloroacetic acids, did not give the product (entries 13 and 14). A small amount of **2a** was formed with *p*-toluenesulfonic acid, but 83% of thiosulfonate was obtained with methanesulfonic acid (entries 15 and 16, respectively). Among the inorganic acids screened (entries 17–21), only hydroiodic acid (HI) provided **2a** in a reasonable yield (68%). We believe that the p*K*_a_ values of the acids (see Table S1) play an important role; however, competing side reactions, such as further oxidation of thiols to sulfonic acids,^31^ may also contribute to the low efficiency observed. Regarding the usage of solvents, we found that this reaction was compatible with toluene (entry 22). In 1,2-dichloroethane, thiophenol was quantitatively converted to disulfide **3a** within 1 h but produced a complex mixture after 3 h; similarly, the reaction in chloroform led to a decomposition mixture after 3 h (entries 23 and 24). This reaction did not proceed in acetonitrile, dimethyl sulfoxide, *N,N*-dimethylformamide, alcohols and ethers (see Table S2 for the complete list). We concluded that the optimal conditions for this transformation are NaNO_3_ (0.1 mmol) and TFA (2.0 mmol) in dichloromethane at room temperature for 1 h. A gram-scale reaction of **1a** to **2a** was also achieved in 92% yield (shown in parentheses, entry 4).

**Table 1:**
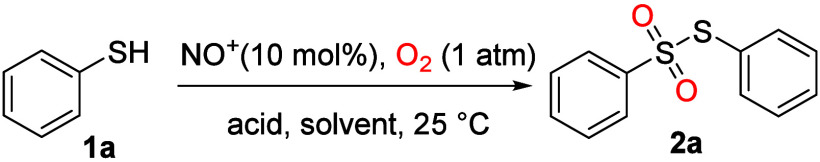
Optimization of Thiosulfonate Formation^a^

entry	NO^+^ source (0.1 equiv)	acid (equiv)	solvent	time (h)	yield (%)
1	NOBF_4_	TFA (2.0)	CH_2_Cl_2_	1	90
2	*t*-C_4_H_9_-ONO	TFA (2.0)	CH_2_Cl_2_	1	94
3	NaNO_2_	TFA (2.0)	CH_2_Cl_2_	1	97
4	NaNO_3_	TFA (2.0)	CH_2_Cl_2_	2	99 (92)^b^
5	HNO_3_ ^c^	TFA (2.0)	CH_2_Cl_2_	1	99
6	–	TFA (2.0)	CH_2_Cl_2_	16	0^c^
7	NaNO_3_	–	CH_2_Cl_2_	16	0^c^
8^d^	NaNO_3_	TFA (2.0)	CH_2_Cl_2_	2	0^c^
9^e^	NaNO_3_	TFA (2.0)	CH_2_Cl_2_	16	98^f^
10	NO_2_BF_4_	TFA (2.0)	CH_2_Cl_2_	2	79
11	NaNO_3_	TFA (1.5)	CH_2_Cl_2_	16	0^c^
12	NaNO_3_	TFA (2.5)	CH_2_Cl_2_	2	84
13	NaNO_3_	CH_3_CO_2_H (2.0)	CH_2_Cl_2_	24	0^c^
14	NaNO_3_	CCl_3_CO_2_H (2.0)	CH_2_Cl_2_	24	0^c^
15	NaNO_3_	*p*-TsOH (2.0)	CH_2_Cl_2_	24	24
16	NaNO_3_	CH_3_SO_3_H (2.0)	CH_2_Cl_2_	3	83
17	NaNO_3_	HCl_(aq)_ (2.0)	CH_2_Cl_2_	24	0^c^
18	NaNO_3_	HBr_(aq)_ (2.0)	CH_2_Cl_2_	16	–^g^
19	NaNO_3_	HI_(aq)_ (2.0)	CH_2_Cl_2_	16	68
20	NaNO_3_	H_2_SO_4_ (2.0)	CH_2_Cl_2_	3	0^c^
21	–	HNO_3_ (2.0)	CH_2_Cl_2_	1	20
22	NaNO_3_	TFA (2.0)	toluene	3	87
23	NaNO_3_	TFA (2.0)	(CH_2_Cl)_2_	1	98^f^
24	NaNO_3_	TFA (2.0)	CHCl_3_	3	–^g^

a A source of nitrosonium ion (0.1 mmol) was added to a solution of **1a** (110.2 mg, 1.0 mmol), TFA (153.1 μL, 2.0 mmol) and a solvent (1.0 mL) at 25 °C. The reaction mixture was stirred at 25 °C under an atmosphere of oxygen (balloon) for the time indicated, and then water (5 mL) was added. The products were obtained after extractions (5 mL CH_2_Cl_2_ × 3), dried over Na_2_SO_4_, filtered through a Celite pad and concentrated.

b **1a** (1.0 g, 9.1 mmol), NaNO_3_ (77.2 mg, 0.91 mmol) and TFA (1.4 mL) was applied to yield **2a** (1.04 g, 4.15 mmol, 92%).

c Starting material **1a** recovered.

d Under nitrogen atmosphere.

e Under air.

f Diphenyl disulfide.

g Decomposed.

After having the optimized reaction conditions in hand, the scope of this thiosulfonate formation was examined using various thiols (Table 2). In addition to thiophenol (**1a**), aryl thiols with alkyl substituents at *para*-, *meta*- and *ortho*-positions were all converted to the corresponding thiosulfonates (**2b**–**2f**) in excellent yields (entries 1–5). The reaction of thiol **1e** was not influenced by the sterically hindered 2,4,6-trimethylphenyl group at the sulfur atom (entry 4). *para*-Substituents on thiophenol, ranging from electron-donating (σ_para_ = −0.14, OMe) and electron-neutral (σ_para_ = 0, NHAc) to electron-withdrawing (σ_para_ = 0.15, 0.24, 0.26, 0.31, 0.47 for fluoro-, chloro-, bromo-, acetate and acetyl group, respectively), have little effect on the reaction (**2g**–**2m**, entries 6–12).^32^ 2-Naphthylthiol was converted to 2-naphthyl thiosulfonate **2n** with a 74% yield (entry 13). For thiols with sulfur bonded to an sp^3^ carbon, or alkyl thiols, a higher loading of TFA (4 equiv) was required to afford the corresponding thiosulfonates (**2o**–**2w**, entries 14–22). We were glad to find that all primary thiols reacted smoothly to give near-quantitative yields (98–99%, **2o**–**2u**), except for volatile *n*-propyl thiol, which furnished **2p** in 81% yield. Secondary thiols, such as isopropyl and cyclohexyl thiols, also provided the corresponding thiosulfonates **2v** and **2w** in slightly lower yields (96% and 98%, respectively; entries 21 and 22). In contrast, tertiary *tert*-butyl thiol (**1x**) exclusively formed disulfide **3x** (entry 23), indicating that disulfide formation precedes oxidation to thiosulfonates and that steric hindrance of the *tert*-butyl group inhibits this subsequent oxidation step. On the basis of this observation, a series of disulfides **3** were selectively prepared by reducing the TFA loading (1–2 equivalence) and shortening the reaction time (Table 3). These include 1,2-diphenyldisulfide (**3a**) and its derivatives bearing either electron-donating or electron-withdrawing substituents, such as 4-methyl (**3b**), 4-methoxy (**3g**), 4-fluoro (**3i**), and 4-nitro (**3y**) groups (entries 2–5). Using 3 equiv of TFA converted alkyl thiols, such as 1-dodecanethiol (**1t**), to 1,2-didodecyldisulfide (**3t**, entry 6). Notably, electron-deficient thiols, including 4-nitrobenzenethiol and pyridine-2-thiol, afforded exclusively the corresponding disulfides **3y** and **3z** (entries 5 and 7). These results indicate that electron-deficient aryl substituents, as well as steric hindrance—as observed for *tert*-butyl thiol—may disfavor further oxidation of disulfides to thiosulfonates. In addition, we observed that glutathione (GSH, **4**) underwent clean oxidation to its disulfide form, GSSG (**5**), a well-established biomarker of oxidative stress,^33^ under this condition (eq 1).

**Table 2:**
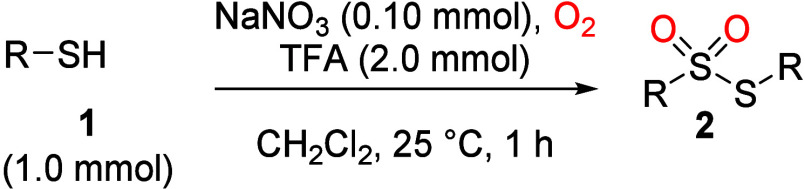
Formation of Thiosulfonates^a^

entry	R	product (% yield)
1	4-CH_3_-C_6_H_4_	**2b** (98)
2	3-CH_3_-C_6_H_4_	**2c** (75)
3	2,6-(CH_3_)_2_-C_6_H_3_	**2d** (83)
4	2,4,6-(CH_3_)_3_-C_6_H_2_	**2e** (88)
5	4-*t*-Bu-C_6_H_4_	**2f** (94)
6	4-OMe-C_6_H_4_	**2g** (93)
7	4-AcHN-C_6_H_4_	**2h** (74)
8	4-F-C_6_H_4_	**2i** (98)
9	4-Cl-C_6_H_4_	**2j** (94)
10	4-Br-C_6_H_4_	**2k** (82)
11	4-CO_2_CH_3_-C_6_H_4_	**2l** (94)
12	4-Ac-C_6_H_4_	**2m** (81)
13	2-naphthyl	**2n** (74)
14^b^	2-phenylethyl	**2o** (99)
15^b^	*n*-propyl	**2p** (81)
16^b^	*n*-butyl	**2q** (98)
17^b^	*n*-hexyl	**2r** (98)
18^b^	*n*-octyl	**2s** (98)
19^b^	*n*-dodecyl	**2t** (98)
20^b^	isobutyl	**2u** (98)
21^b^	isopropyl	**2v** (96)
22^b^	cyclohexyl	**2w** (98)
23^b^	*tert*-butyl	(*t*-BuS)_2_, **3x** (94)

a Sodium nitrate (0.10 mmol) was added to a solution of thiol (1.0 mmol), dichloromethane (1.0 mL), and trifluoroacetic acid (153.1 μL, 2.0 mmol). The reaction mixture was stirred at rt for 1 h under an oxygen atmosphere (balloon).

b TFA (306.0 μL, 4.0 mmol).

**Table 3:**
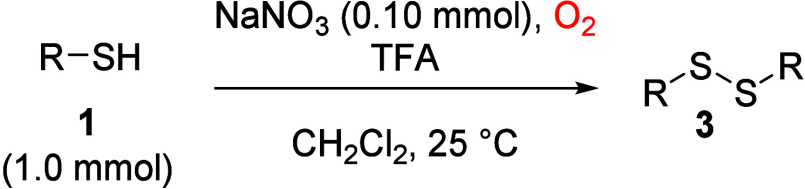
Formation of Disulfides

entry	R	condition	Product (% yield)
1	phenyl	TFA (2.0 equiv), 25 °C, 30 min	**3a** (98)
2	4-Me-C_6_H_4_	TFA (1.0 equiv), 25 °C, 1 h	**3b** (97)
3	4-OMe-C_6_H_4_	TFA (1.0 equiv), 25 °C, 3 h	**3g** (98)
4	4–F-C_6_H_4_	TFA (1.0 equiv), 25 °C, 3 h	**3i** (98)
5	4-NO_2_-C_6_H_4_	TFA (2.0 equiv), 25 °C, 3 h	**3y** (98)
6	*n*-dodecyl	TFA (3.0 equiv), 25 °C, 3 h	**3t** (98)
7	2-pyridinyl	TFA (4.0 equiv), 25 °C, 16 h	**3z** (95)



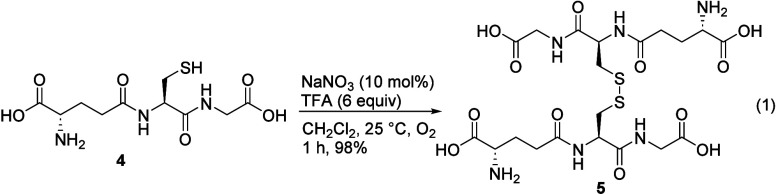

1


The stepwise oxidation of thiol **1** to disulfide **3** and then to thiosulfonate **2** was further confirmed by monitoring the reaction of 4-methylbenzenethiol (**1b**) with ^1^H NMR, in which the methyl signals of the three species were clearly resolved in the range of 2.1 ∼ 2.4 ppm (Figure 2). After the addition of sodium nitrate to the reaction mixture, **1b** was rapidly consumed, achieving complete conversion within 40 min to form disulfide **3b**, followed by the gradual conversion of **3b** into thiosulfonate **2b** over the next 30 min. Indeed, treating disulfide **3a** under the nitrosonium-catalyzed aerobic oxidation condition also afforded thiosulfonate **2a** in 96% yield (see the Experimental Section). We also observed that this reaction was inhibited by 2,6-di-*tert*-butyl-4-methylphenol (BHT) and 2,2,6,6-tetramethyl-1-piperidinyloxyl (TEMPO).

**Figure 2:**
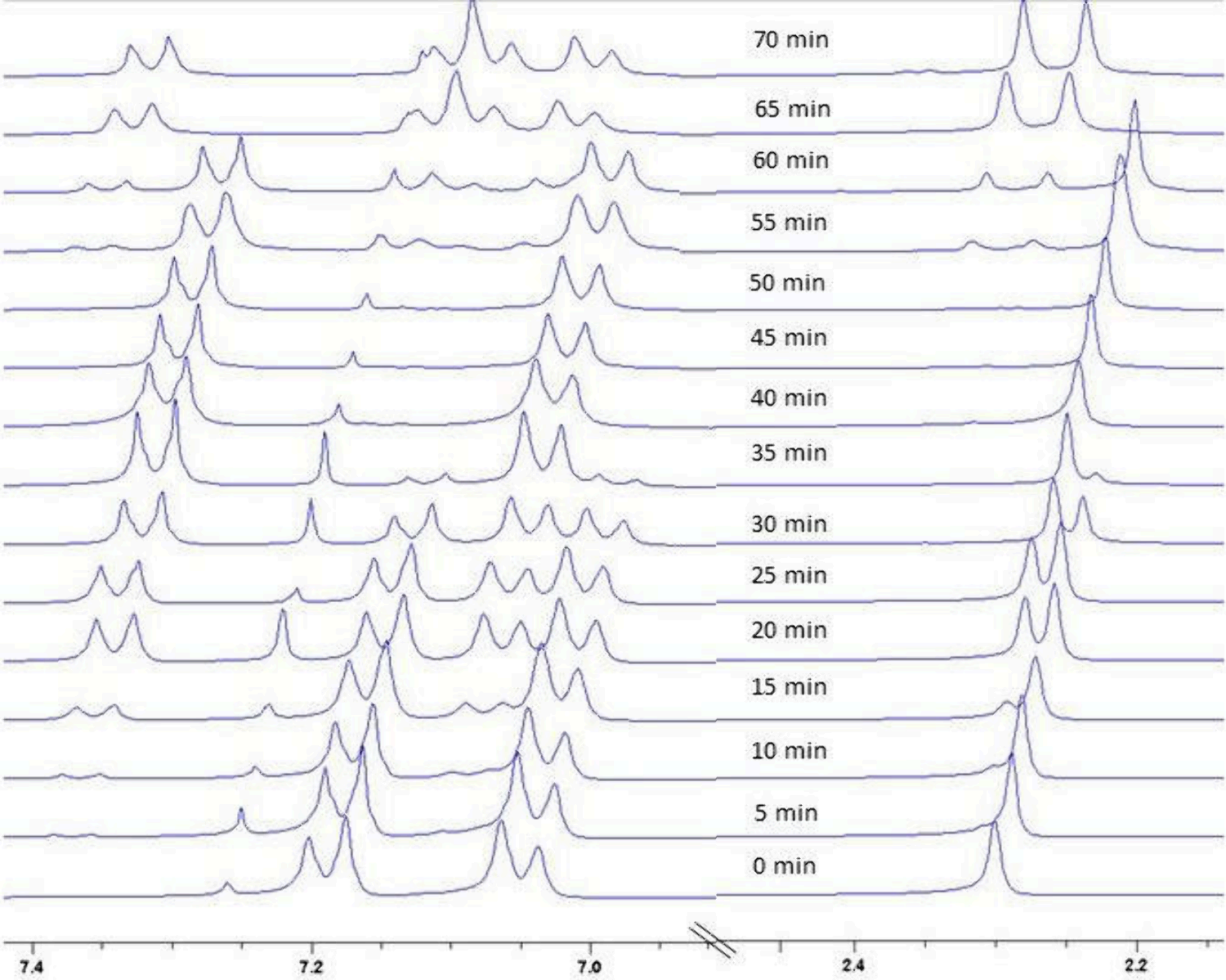
Stacked ^1^H NMR spectra (300 MHz, CDCl_3_) recorded during the oxidative conversion of **1b**.

The reaction mechanism is depicted in Scheme 2. Nitrosonium ion reacts with thiol **1** to form the corresponding *S*-nitrosothiol **6**, which subsequently reacts with another equivalent of thiol to afford disulfide **3**.^34^ Nitrosonium ions are regenerated from nitroxyl (azanone, HNO) by molecular oxygen under acidic conditions. Complete consumption of the thiol and accumulation of the disulfide were observed (Figure 2), enabling the isolation of disulfide **3** as shown in Table 3. Further oxidation of the disulfide to thiosulfonate is proposed to proceed via NO^+^-mediated, stepwise oxygenation, forming thiosulfinate **7**, disulfoxide **8**, and subsequent rearrangement to thiosulfonate **2**.^28a,35^ Alternatively, direct oxidation of thiosulfinate **7** to the corresponding thiosulfonate has also been proposed.^35,36^ Consistent with our previous observations in NO^+^-mediated sulfide oxidation,^29b^ no H_2_O_2_ was detected in the reaction medium using starch-iodide test (Figure S1), supporting a distinct NO^+^-driven oxygenation pathway. Previous oxidation methods relied on stoichiometric amounts of nitrogen oxides, such as N_2_O_4_;^17,37^ in contrast, the presence of TFA and molecular oxygen enables nitrosonium recycling, thereby facilitating efficient thiol conversion.

**Scheme 2:**
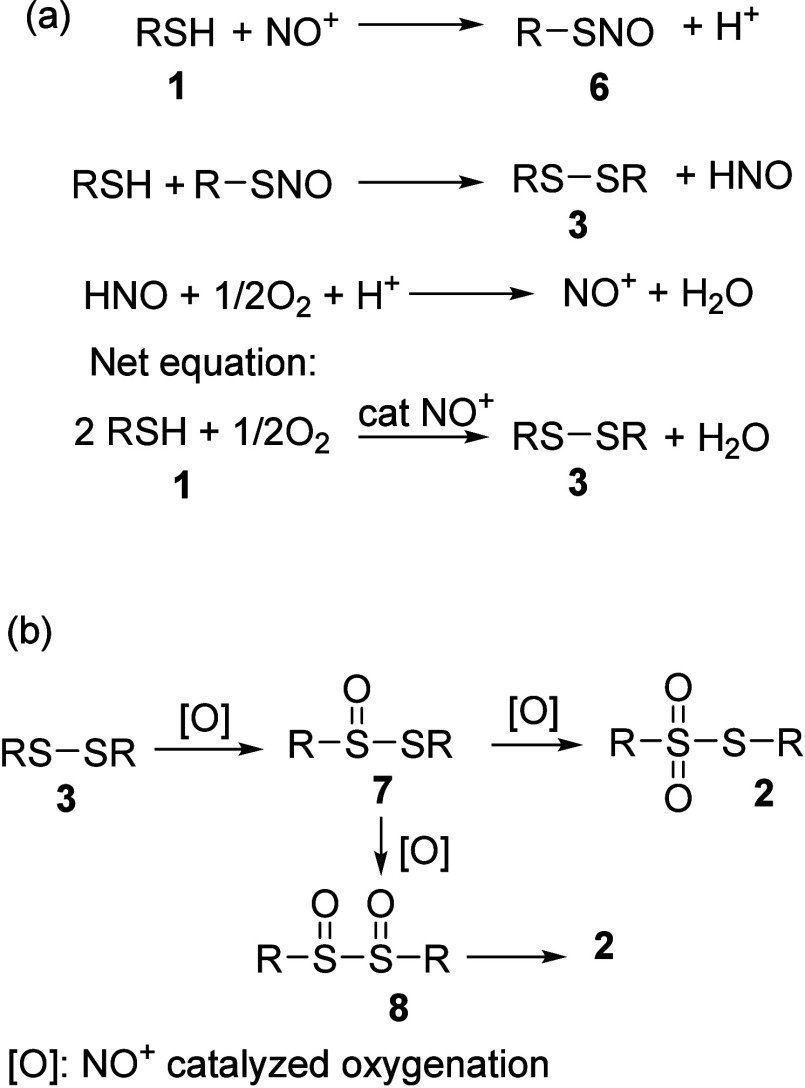
Reaction Mechanism for the Formation of (a) Disulfide and (b) Thiosulfonates

## Conclusion

In summary, we have developed a practical and efficient method for the selective oxidation of thiols. The reaction is catalyzed by NO^+^, generated *in situ* from NaNO_3_ in the presence of trifluoroacetic acid, and proceeds cooperatively with molecular oxygen at room temperature. Compared with previously reported aerobic thiol oxidations, this metal-free and mild system enables a stepwise, controllable conversion of thiols to disulfides or thiosulfonates with particularly high efficiency for aliphatic thiols.

## Experimental Section

### General Information

Reagents, such as NaNO_2_, NaNO_3_, NOBF_4_, NO_2_BF_4_, *tert*-butyl nitrite, sulfuric acid, TFA, and acetic acid, were purchased from commercial sources (ACS grade) and used without further purification. Thin-layer chromatography (TLC) was conducted using precoated silica gel 60 F_254_ plates containing a fluorescent indicator; spots were examined under UV light or revealed by KMnO_4_ solution. Purification by chromatography was conducted using silica gel (230–400 mesh). The ^1^H and ^13^C NMR spectra were recorded in a CDCl_3_ or D_2_O solution by using a Bruker Avance 500 and 300 NMR spectrometer. Chemical shifts for ^1^H and ^13^C NMR spectra are reported in δ units (parts per million) with reference to residual solvent peaks. High-resolution mass spectrometry (HRMS) data were recorded on a JMS-700 quadrupole mass spectrometer. ***Caution!*** Trifluoroacetic acid, a GHS Category 1 corrosive chemical; dichloromethane, a serious eye and skin irritant; and toxic chemicals, including many foul-smelling thiols, pose significant safety hazards. All reactions involving these reagents should be conducted in a well-ventilated fume hood with the appropriate personal protective equipment.

#### S-Phenyl Benzenesulfonothioate (**2a**)

Sodium nitrate (8.5 mg, 0.10 mmol) was added to a solution of thiophenol (**1a**, 110.2 mg, 1.0 mmol) and trifluoroacetic acid (153 μL, 228.0 mg, 2.0 mmol) in dichloromethane (1.0 mL) at 25 °C. The resulting reaction mixture was stirred at 25 °C for 1 h under an oxygen atmosphere (O_2_ balloon). After completion, the mixture was filtered through a pad of Celite and eluted with dichloromethane (10 mL). The filtrate was added with water (10 mL) and extracted with dichloromethane (3 × 5 mL). The combined organic layers were dried over anhydrous sodium sulfate, filtered, and concentrated under reduced pressure. The crude product was purified by column chromatography (SiO_2_, EtOAc/hexanes, 1:9, *R*_*f*_ 0.52) to give compound **2a** (123.3 mg, 0.49 mmol, 98%) as a colorless solid. Mp 40.0–42.0 °C; ^1^H NMR (300 MHz, CDCl_3_) δ 7.59–7.53 (m, 3H), 7.48–7.45 (m, 1H), 7.44–7.37 (m, 2H), 7.34–7.28 (m, 4H); ^13^C­{^1^H} NMR (75 MHz, CDCl_3_) δ 142.8, 136.6, 133.8, 131.5, 129.5, 128.9, 127.8, 127.5. The spectroscopic data were consistent with the reported values.^11d^ Compound **2a** was also prepared from diphenyldisulfide **3a** (218.4 mg, 1.0 mmol), sodium nitrate (8.5 mg, 0.10 mmol), and TFA (153 μL, 2.0 mmol) using the same procedure to give **2a** in 96% yield (239.6 mg, 0.96 mmol). The reactions were completely inhibited with BHT and TEMPO (0.3 mmol), and only **1a** was observed in the TLC analysis.

#### S-(p-Tolyl) 4-Methylbenzenesulfonothioate (**2b**)

Sodium nitrate (8.5 mg, 0.10 mmol) was added to a solution of 4-methylbenzenethiol (**1b**, 124.2 mg, 1.0 mmol) and trifluoroacetic acid (153 μL, 228.0 mg, 2.0 mmol) in dichloromethane (1.0 mL) at 25 °C. The resulting reaction mixture was stirred at 25 °C for 1 h under an oxygen atmosphere (O_2_ balloon). After completion, the mixture was filtered through a pad of Celite and eluted with dichloromethane (10 mL). The filtrate was added with water (10 mL) and extracted with dichloromethane (3 × 5 mL). The combined organic layers were dried over anhydrous sodium sulfate, filtered, and concentrated under reduced pressure. The crude product was purified by column chromatography (SiO_2_, EtOAc/hexanes, 1:9, *R*_*f*_ 0.56) to give compound **2b** (135.1 mg, 0.49 mmol, 98%) as a colorless solid. Mp 74.0–76.0 °C; ^1^H NMR (300 MHz, CDCl_3_) δ 7.44 (d, *J* = 8.1 Hz, 2H), 7.24–7.19 (m, 4H), 7.13 (d, *J* = 8.1 Hz, 2H), 2.41 (s, 3H), 2.37 (s, 3H); ^13^C­{^1^H} NMR (75 MHz, CDCl_3_) δ 144.7, 142.1, 140.3, 136.4, 130.2, 129.4, 127.5, 124.4, 21.6, 21.4. The spectroscopic data were consistent with the reported values.^11d^

#### S-(m-Tolyl) 3-Methylbenzenesulfonothioate (**2c**)

Sodium nitrate (8.5 mg, 0.10 mmol) was added to a solution of 3-methylbenzenethiol (**1b**, 124.2 mg, 1.0 mmol) and trifluoroacetic acid (153 μL, 228.0 mg, 2.0 mmol) in dichloromethane (1.0 mL) at 25 °C. The resulting reaction mixture was stirred at 25 °C for 1 h under an oxygen atmosphere (O_2_ balloon). After completion, the mixture was filtered through a pad of Celite and eluted with dichloromethane (10 mL). The filtrate was added with water (10 mL) and extracted with dichloromethane (3 × 5 mL). The combined organic layers were dried over anhydrous sodium sulfate, filtered, and concentrated under reduced pressure. The crude product was purified by column chromatography (SiO_2_, EtOAc/hexanes, 1:9, *R*_*f*_ 0.55) to give compound **2c** (104.2 mg, 0.37 mmol, 75%) as a colorless liquid. ^1^H NMR (300 MHz, CDCl_3_) δ 7.42–7.37 (m, 3H), 7.35–7.27 (m, 2H), 7.24–22 (m, 1H), 7.18–7.16 (m, 2H), 2.37 (s, 3H), 2.32 (s, 3H); ^13^C­{^1^H} NMR (75 MHz, CDCl_3_) δ 142.9, 139.5, 139.1, 137.3, 134.4, 133.8, 132.3, 129.3, 128.7, 128.1, 127.7, 124.9, 21.3. The spectroscopic data were consistent with the reported values.^11d^

#### S-(2,6-Dimethylphenyl) 2,6-Dimethylbenzenesulfonothioate (**2d**)

Sodium nitrate (8.5 mg, 0.10 mmol) was added to a solution of 2,6-dimethylbenzenethiol (**1d**, 138.2 mg, 1.0 mmol) and trifluoroacetic acid (153 μL, 228.0 mg, 2.0 mmol) in dichloromethane (1.0 mL) at 25 °C. The resulting reaction mixture was stirred at 25 °C for 1 h under an oxygen atmosphere (O_2_ balloon). After completion, the mixture was filtered through a pad of Celite and eluted with dichloromethane (10 mL). The filtrate was added with water (10 mL) and extracted with dichloromethane (3 × 5 mL). The combined organic layers were dried over anhydrous sodium sulfate, filtered, and concentrated under reduced pressure. The crude product was purified by column chromatography (SiO_2_, EtOAc/hexanes, 1:9, *R*_*f*_ 0.58) to give compound **2d** (126.7 mg, 0.41 mmol, 83%) as a colorless solid. Mp 118.0–120.0 °C; ^1^H NMR (300 MHz, CDCl_3_) δ 7.35–7.30 (m, 1H), 7.28–7.23 (m, 1H), 7.11–7.09 (m, 4H), 2.41 (s, 6H), 2.21 (s, 6H); ^13^C­{^1^H} NMR (75 MHz, CDCl_3_) δ 145.8, 142.1, 139.9, 133.0, 131.6, 131.5, 128.7, 126.6, 22.9, 21.6. The spectroscopic data were consistent with the reported values.^38^

#### S-Mesityl 2,4,6-Trimethylbenzenesulfonothioate (**2e**)

Sodium nitrate (8.5 mg, 0.10 mmol) was added to a solution of 2,4,6-trimethylbenzenethiol (**1e**, 152.3 mg, 1.0 mmol) and trifluoroacetic acid (153 μL, 228.0 mg, 2.0 mmol) in dichloromethane (1.0 mL) at 25 °C. The resulting reaction mixture was stirred at 25 °C for 1 h under an oxygen atmosphere (O_2_ balloon). After completion, the mixture was filtered through a pad of Celite and eluted with dichloromethane (10 mL). The filtrate was added with water (10 mL) and extracted with dichloromethane (3 × 5 mL). The combined organic layers were dried over anhydrous sodium sulfate, filtered, and concentrated under reduced pressure. The crude product was purified by column chromatography (SiO_2_, EtOAc/hexanes, 1:9, *R*_*f*_ 0.62) to give compound **2e** (146.3 mg, 0.44 mmol, 88%) as a colorless solid. Mp 130.0–132.0 °C; ^1^H NMR (300 MHz, CDCl_3_) δ 6.90–6.88 (m, 4H), 2.35 (s, 6H), 2.30 (s, 3H), 2.27 (s, 3H), 2.15 (s, 6H); ^13^C­{^1^H} NMR (75 MHz, CDCl_3_) δ 145.5, 143.5, 141.9, 139.8, 139.5, 132.0, 129.6, 123.4, 22.7, 21.5, 21.3, 21.1. The spectroscopic data were consistent with the reported values.^11d^

#### S-(4-(*tert*-Butyl)­phenyl) 4-(*tert*-Butyl)­benzenesulfonothioate (**2f**)

Sodium nitrate (8.5 mg, 0.10 mmol) was added to a solution of 4-(*tert*-butyl)­benzenethiol (**1f**, 166.3 mg, 1.0 mmol) and trifluoroacetic acid (153 μL, 228.0 mg, 2.0 mmol) in dichloromethane (1.0 mL) at 25 °C. The resulting reaction mixture was stirred at 25 °C for 1 h under an oxygen atmosphere (O_2_ balloon). After completion, the mixture was filtered through a pad of Celite and eluted with dichloromethane (10 mL). The filtrate was added with water (10 mL) and extracted with dichloromethane (3 × 5 mL). The combined organic layers were dried over anhydrous sodium sulfate, filtered, and concentrated under reduced pressure. The crude product was purified by column chromatography (SiO_2_, EtOAc/hexanes, 1:9, *R*_*f*_ 0.54) to give compound **2f** (169.7 mg, 0.47 mmol, 94%) as a colorless solid. Mp 145.0–147.0 °C; ^1^H NMR (300 MHz, CDCl_3_) δ 7.48 (d, *J* = 8.7 Hz, 2H), 7.40 (d, *J* = 8.7 Hz, 2H), 7.33 (d, *J* = 8.4 Hz, 2H), 7.27 (d, *J* = 8.4 Hz, 2H), 1.33 (s, 9H), 1.31 (s, 9H); ^13^C­{^1^H} NMR (75 MHz, CDCl_3_) δ 157.7, 155.1, 140.2, 136.4, 127.5, 126.5, 125.8, 124.7, 35.3, 35.0, 31.2, 31.1. The spectroscopic data were consistent with the reported values.^11d^

#### S-(4-Methoxyphenyl) 4-Methoxybenzenesulfonothioate (**2g**)

Sodium nitrate (8.5 mg, 0.10 mmol) was added to a solution of 4-methoxybenzenethiol (**1g**, 140.2 mg, 1.0 mmol) and trifluoroacetic acid (153 μL, 228.0 mg, 2.0 mmol) in dichloromethane (1.0 mL) at 25 °C. The resulting reaction mixture was stirred at 25 °C for 1 h under an oxygen atmosphere (O_2_ balloon). After completion, the mixture was filtered through a pad of Celite and eluted with dichloromethane (10 mL). The filtrate was added with water (10 mL) and extracted with dichloromethane (3 × 5 mL). The combined organic layers were dried over anhydrous sodium sulfate, filtered, and concentrated under reduced pressure. The crude product was purified by column chromatography (SiO_2_, EtOAc/hexanes, 1:9, *R*_*f*_ 0.42) to give compound **2g** (143.4 mg, 0.46 mmol, 93%) as a colorless solid. Mp 90.0–92.0 °C; ^1^H NMR (300 MHz, CDCl_3_) δ 7.51 (d, *J* = 9.0 Hz, 2H), 7.28 (d, *J* = 9.0 Hz, 2H), 6.90–6.84 (m, 4H), 3.88 (s, 3H), 3.84 (s, 3H); ^13^C­{^1^H} NMR (75 MHz, CDCl_3_) δ 163.6, 162.3, 138.4, 134.9, 130.0, 119.0, 115.0, 113.9, 55.8, 55.6. The spectroscopic data were consistent with the reported values.^11d^

#### S-(4-Acetamidophenyl) 4-Acetamidobenzenesulfonothioate (**2h**)

Sodium nitrate (8.5 mg, 0.10 mmol) was added to a solution of *N*-(4-mercaptophenyl)­acetamide (**1h**, 167.2 mg, 1.0 mmol) and trifluoroacetic acid (153 μL, 228.0 mg, 2.0 mmol) in dichloromethane (1.0 mL) at 25 °C. The resulting reaction mixture was stirred at 25 °C for 1 h under an oxygen atmosphere (O_2_ balloon). After completion, the mixture was filtered through a pad of Celite and eluted with dichloromethane (10 mL). The filtrate was added with water (10 mL) and extracted with dichloromethane (3 × 5 mL). The combined organic layers were dried over anhydrous sodium sulfate, filtered, and concentrated under reduced pressure. The crude product was purified by column chromatography (SiO_2_, EtOAc/hexanes, 1:9, *R*_*f*_ 0.2) to give compound **2h** (134.3 mg, 0.37 mmol, 74%) as a colorless solid. Mp 226.0–228.0 °C; ^1^H NMR (300 MHz, DMSO-*d*_6_) δ 10.5 (s, 1H), 10.3 (s, 1H), 7.72 (d, *J* = 9.0 Hz, 2H), 7.61 (d, *J* = 8.7 Hz, 2H), 7.47 (d, *J* = 9.0 Hz, 2H), 7.23 (d, *J* = 8.7 Hz, 2H), 2.09 (s, 3H), 2.06 (s, 3H); ^13^C­{^1^H} NMR (75 MHz, DMSO-*d*_6_) δ 170.0, 169.7, 144.7, 142.8, 137.6, 136.0, 129.1, 120.5, 119.9, 118.9, 40.5, 40.3, 40.0, 39.7, 39.4, 39.2, 38.9, 24.6, 24.5. The spectroscopic data were consistent with the reported values.^39^

#### S-(4-Fluorophenyl) 4-Fluorobenzenesulfonothioate (**2i**)

Sodium nitrate (8.5 mg, 0.10 mmol) was added to a solution of 4-fluorobenzenethiol (**1i**, 128.2 mg, 1.0 mmol) and trifluoroacetic acid (153 μL, 228.0 mg, 2.0 mmol) in dichloromethane (1.0 mL) at 25 °C. The resulting reaction mixture was stirred at 25 °C for 1 h under an oxygen atmosphere (O_2_ balloon). After completion, the mixture was filtered through a pad of Celite and eluted with dichloromethane (10 mL). The filtrate was added with water (10 mL) and extracted with dichloromethane (3 × 5 mL). The combined organic layers were dried over anhydrous sodium sulfate, filtered, and concentrated under reduced pressure. The crude product was purified by column chromatography (SiO_2_, EtOAc/hexanes, 1:9, *R*_*f*_ 0.52) to give compound **2i** (140.8 mg, 0.49 mmol, 98%) as a colorless solid. Mp 64.0–66.0 °C; ^1^H NMR (300 MHz, CDCl_3_) δ 7.61–7.56 (m, 2H), 7.38–7.33 (m, 2H), 7.14–7.03 (m, 4H); ^13^C­{^1^H} NMR (75 MHz, CDCl_3_) δ 165.7 (d, *J* = 256.0 Hz), 165.0 (d, *J* = 253.0 Hz), 139.0, 138.9, 130.6 (d, *J* = 10.0 Hz), 123.3, 117.1 (d, *J* = 22.0 Hz), 116.3 (d, *J* = 23.0 Hz); ^19^F-NMR (283 MHz, CDCl_3_) δ – 102.34, 106.8. The spectroscopic data were consistent with the reported values.^11d^

#### S-(4-Chlorophenyl) 4-chlorobenzenesulfonothioate (**2j**)

Sodium nitrate (8.5 mg, 0.10 mmol) was added to a solution of 4-chlorobenzenethiol (**1j**, 144.6 mg, 1.0 mmol) and trifluoroacetic acid (153 μL, 228.0 mg, 2.0 mmol) in dichloromethane (1.0 mL) at 25 °C. The resulting reaction mixture was stirred at 25 °C for 1 h under an oxygen atmosphere (O_2_ balloon). After completion, the mixture was filtered through a pad of Celite and eluted with dichloromethane (10 mL). The filtrate was added with water (10 mL) and extracted with dichloromethane (3 × 5 mL). The combined organic layers were dried over anhydrous sodium sulfate, filtered, and concentrated under reduced pressure. The crude product was purified by column chromatography (SiO_2_, EtOAc/hexanes, 1:9, *R*_*f*_ 0.49) to give compound **2j** (149.8 mg, 0.47 mmol, 94%) as a colorless solid. Mp 130.0–132.0 °C; ^1^H NMR (300 MHz, CDCl_3_) δ 7.51 (d, *J* = 8.7 Hz, 2H), 7.42 (d, *J* = 8.7 Hz, 2H), 7.35–7.28 (m, 4H); ^13^C­{^1^H} NMR (75 MHz, CDCl_3_) δ 141.4, 140.7, 138.6, 137.8, 130.0, 129.4, 129.0, 126.1. The spectroscopic data were consistent with the reported values.^11d^

#### S-(4-Bromophenyl) 4-bromobenzenesulfonothioate (**2k**)

Sodium nitrate (8.5 mg, 0.10 mmol) was added to a solution of 4-bromobenzenethiol (**1k**, 189.1 mg, 1.0 mmol) and trifluoroacetic acid (153 μL, 228.0 mg, 2.0 mmol) in dichloromethane (1.0 mL) at 25 °C. The resulting reaction mixture was stirred at 25 °C for 1 h under an oxygen atmosphere (O_2_ balloon). After completion, the mixture was filtered through a pad of Celite and eluted with dichloromethane (10 mL). The filtrate was added with water (10 mL) and extracted with dichloromethane (3 × 5 mL). The combined organic layers were dried over anhydrous sodium sulfate, filtered, and concentrated under reduced pressure. The crude product was purified by column chromatography (SiO_2_, EtOAc/hexanes, 1:9, *R*_*f*_ 0.51) to give compound **2k** (168.5 mg, 0.41 mmol, 82%) as a colorless solid. Mp 148.0–150.0 °C; ^1^H NMR (300 MHz, CDCl_3_) δ 7.60 (d, *J* = 8.4 Hz, 2H), 7.51 (d, *J* = 8.4 Hz, 2H), 7.44 (d, *J* = 8.7 Hz, 2H), 7.24 (d, *J* = 8.1 Hz, 2H); ^13^C­{^1^H} NMR (75 MHz, CDCl_3_) δ 142.0, 138.0, 133.1, 132.4, 129.3, 129.1, 127.2, 126.7. The spectroscopic data were consistent with the reported values.^11d^

#### Methyl 4-(((4-(Methoxy­carbonyl)­phenyl)­thio)­sulfonyl)­benzoate (**2l**)

Sodium nitrate (8.5 mg, 0.10 mmol) was added to a solution of methyl 4-mercaptobenzoate (**1l**, 166.3 mg, 1.0 mmol) and trifluoroacetic acid (153 μL, 228.0 mg, 2.0 mmol) in dichloromethane (1.0 mL) at 25 °C. The resulting reaction mixture was stirred at 25 °C for 1 h under an oxygen atmosphere (O_2_ balloon). After completion, the mixture was filtered through a pad of Celite and eluted with dichloromethane (10 mL). The filtrate was added with water (10 mL) and extracted with dichloromethane (3 × 5 mL). The combined organic layers were dried over anhydrous sodium sulfate, filtered, and concentrated under reduced pressure. The crude product was purified by column chromatography (SiO_2_, EtOAc/hexanes, 1:9, *R*_*f*_ 0.54) to give compound **2l** (169.7 mg, 0.47 mmol, 94%) as a colorless solid. Mp 142.0–144.0 °C; ^1^H NMR (300 MHz, CDCl_3_) δ 8.07 (d, *J* = 8.4 Hz, 2H), 7.98 (d, *J* = 8.4 Hz, 2H), 7.61 (d, *J* = 8.7 Hz, 2H), 7.43 (d, *J* = 8.4 Hz, 2H), 3.94 (s, 3H), 3.93 (s, 3H); ^13^C­{^1^H} NMR (75 MHz, CDCl_3_) δ 166.0, 165.3, 146.4, 136.4, 134.9, 133.0, 132.5, 130.6, 127.6, 52.9, 52.7. The spectroscopic data were consistent with the reported values.^21a^

#### S-(4-Acetylphenyl) 4-Acetylbenzenesulfonothioate (**2m**)

Sodium nitrate (8.5 mg, 0.10 mmol) was added to a solution of 1-(4-mercaptophenyl)­ethan-1-one (**1m**, 76.2 mg, 0.50 mmol) and trifluoroacetic acid (76 μL, 114.0 mg, 1.0 mmol) in dichloromethane (1.0 mL) at 25 °C. The resulting reaction mixture was stirred at 25 °C for 1 h under an oxygen atmosphere (O_2_ balloon). After completion, the mixture was filtered through a pad of Celite and eluted with dichloromethane (10 mL). The filtrate was added with water (10 mL) and extracted with dichloromethane (3 × 5 mL). The combined organic layers were dried over anhydrous sodium sulfate, filtered, and concentrated under reduced pressure. The crude product was purified by column chromatography (SiO_2_, EtOAc/hexanes, 1:9, *R*_*f*_ 0.45) to give compound **2m** (67.4 mg, 0.20 mmol, 81%) as a colorless solid. Mp 126.0–128.0 °C; ^1^H NMR (300 MHz, CDCl_3_) δ 7.99 (d, *J* = 8.4 Hz, 2H), 7.91 (d, *J* = 8.1 Hz, 2H), 7.67 (d, *J* = 8.4 Hz, 2H), 7.49 (d, *J* = 8.1 Hz, 2H), 2.64 (s, 3H), 2.62 (s, 3H); ^13^C­{^1^H} NMR (75 MHz, CDCl_3_) δ 197.2, 196.6, 146.5, 140.9, 139.2, 136.6, 132.6, 129.3, 129.0, 127.9, 27.1, 26.9. The spectroscopic data were consistent with the reported values.^39^

#### S-(Naphthalen-2-yl) Naphthalene-2-sulfonothioate (**2n**)

Sodium nitrate (8.5 mg, 0.10 mmol) was added to a solution of naphthalene-2-thiol (**1n**, 160.2 mg, 1.0 mmol) and trifluoroacetic acid (153 μL, 228.0 mg, 2.0 mmol) in dichloromethane (1.0 mL) at 25 °C. The resulting reaction mixture was stirred at 25 °C for 1 h under an oxygen atmosphere (O_2_ balloon). After completion, the mixture was filtered through a pad of Celite and eluted with dichloromethane (10 mL). The filtrate was added with water (10 mL) and extracted with dichloromethane (3 × 5 mL). The combined organic layers were dried over anhydrous sodium sulfate, filtered, and concentrated under reduced pressure. The crude product was purified by column chromatography (SiO_2_, EtOAc/hexanes, 1:9, *R*_*f*_ 0.30) to give compound **2n** (127.4 mg, 0.37 mmol, 74%) as a colorless solid. Mp 98.0–100.0 °C; ^1^H NMR (300 MHz, CDCl_3_) δ 7.91–7.87 (m, 2H), 7.84–7.82 (m, 2H), 7.73 (d, *J* = 8.4 Hz, 1H), 7.67–7.63 (m, 3H), 7.61–7.60 (m, 1H), 7.58–7.53 (m, 2H), 7.51–7.45 (m, 1H), 7.35 (dd, *J* = 8.4, 6.9 Hz, 1H); ^13^C­{^1^H} NMR (75 MHz, CDCl_3_) δ139.8, 137.8, 135.2, 134.2, 133.4, 132.0, 131.7, 129.6, 129.5, 129.4, 129.3, 129.2, 128.5, 128.4, 128.0, 127.9, 127.8, 127.0, 125.3, 122.5. The spectroscopic data were consistent with the reported values.^11d^

#### S-Phenethyl 2-Phenylethane-1-sulfonothioate (**2o**)

Sodium nitrate (8.5 mg, 0.10 mmol) was added to a solution of 2-phenylethane-1-thiol (**1o**, 138.2 mg, 1.0 mmol) and trifluoroacetic acid (153 μL, 228.0 mg, 2.0 mmol) in dichloromethane (1.0 mL) at 25 °C. The resulting reaction mixture was stirred at 25 °C for 1 h under an oxygen atmosphere (O_2_ balloon). After completion, the mixture was filtered through a pad of Celite and eluted with dichloromethane (10 mL). The filtrate was added with water (10 mL) and extracted with dichloromethane (3 × 5 mL). The combined organic layers were dried over anhydrous sodium sulfate, filtered, and concentrated under reduced pressure. The crude product was purified by column chromatography (SiO_2_, EtOAc/hexanes, 1:9, *R*_*f*_ 0.32) to give compound **2o** (151.4 mg, 0.49 mmol, 99%) as a colorless solid. Mp 42.0–44.0 °C; ^1^H NMR (300 MHz, CDCl_3_) δ 7.42–7.33 (m, 5H), 7.30–7.28 (m, 3H), 7.24–7.22 (m, 2H), 3.50–3.43 (m, 4H), 3.24–3.18 (m, 2H), 3.11 (t, *J* = 6.0 Hz, 2H); ^13^C­{^1^H} NMR (75 MHz, CDCl_3_) δ 138.5, 136.9, 128.8, 128.7, 128.5, 127.1, 127.0 63.4, 37.6, 36.0, 29.5. The spectroscopic data were consistent with the reported values.^15a^

#### S-Propyl Propane-1-sulfonothioate (**2p**)

Sodium nitrate (8.5 mg, 0.10 mmol) was added to a solution of propane-1-thiol (**1p**, 76.2 mg, 1.0 mmol) and trifluoroacetic acid (306 μL, 456.0 mg, 4.0 mmol) in dichloromethane (1.0 mL) at 25 °C. The resulting reaction mixture was stirred at 25 °C for 1 h under an oxygen atmosphere (O_2_ balloon). After completion, the mixture was filtered through a pad of Celite and eluted with dichloromethane (10 mL). The filtrate was added with water (10 mL) and extracted with dichloromethane (3 × 5 mL). The combined organic layers were dried over anhydrous sodium sulfate, filtered, and concentrated under reduced pressure. The crude product was purified by column chromatography (SiO_2_, EtOAc/hexanes, 1:9, *R*_*f*_ 0.76) to give compound **2p** (73.6 mg, 0.40 mmol, 81%) as a colorless liquid. ^1^H NMR (300 MHz, CDCl_3_) δ 3.31–3.25 (m, 2H), 3.11 (t, *J* = 7.2 Hz, 2H), 2.02–1.89 (m, 2H), 1.82–1.74 (m, 2H), 1.11–1.01 (m, 6H); ^13^C­{^1^H} NMR (75 MHz, CDCl_3_) δ 64.5, 38.3, 23.3, 17.5, 13.3, 12.8. The spectroscopic data were consistent with the reported values.^11d^

#### S-Butyl Butane-1-sulfonothioate (**2q**)

Sodium nitrate (8.5 mg, 0.10 mmol) was added to a solution of butane-1-thiol (**1q**, 90.2 mg, 1.0 mmol) and trifluoroacetic acid (306 μL, 456.0 mg, 4.0 mmol) in dichloromethane (1.0 mL) at 25 °C. The resulting reaction mixture was stirred at 25 °C for 1 h under an oxygen atmosphere (O_2_ balloon). After completion, the mixture was filtered through a pad of Celite and eluted with dichloromethane (10 mL). The filtrate was added with water (10 mL) and extracted with dichloromethane (3 × 5 mL). The combined organic layers were dried over anhydrous sodium sulfate, filtered, and concentrated under reduced pressure. The crude product was purified by column chromatography (SiO_2_, EtOAc/hexanes, 1:9, *R*_*f*_ 0.66) to give compound **2q** (103.7 mg, 0.49 mmol, 98%) as a colorless liquid. ^1^H NMR (300 MHz, CDCl_3_) δ 3.30–3.25 (m, 2H), 3.13–3.08 (m, 2H), 1.91–1.80 (m, 2H), 1.74–1.65 (m, 2H), 1.52–1.35 (m, 4H), 0.96–0.88 (m, 6H); ^13^C­{^1^H} NMR (75 MHz, CDCl_3_) δ 62.4, 36.0, 31.7, 25.5, 21.8, 21.3, 13.6, 13.5. The spectroscopic data were consistent with the reported values.^21a^

#### S-Hexyl Hexane-1-sulfonothioate (**2r**)

Sodium nitrate (8.5 mg, 0.10 mmol) was added to a solution of hexane-1-thiol (**1r**, 118.2 mg, 1.0 mmol) and trifluoroacetic acid (306 μL, 456.0 mg, 4.0 mmol) in dichloromethane (1.0 mL) at 25 °C. The resulting reaction mixture was stirred at 25 °C for 1 h under an oxygen atmosphere (O_2_ balloon). After completion, the mixture was filtered through a pad of Celite and eluted with dichloromethane (10 mL). The filtrate was added with water (10 mL) and extracted with dichloromethane (3 × 5 mL). The combined organic layers were dried over anhydrous sodium sulfate, filtered, and concentrated under reduced pressure. The crude product was purified by column chromatography (SiO_2_, EtOAc/hexanes, 1:9, *R*_*f*_ 0.66) to give compound **2r** (130.4 mg, 0.49 mmol, 98%) as a colorless liquid. ^1^H NMR (300 MHz, CDCl_3_) δ 3.25 (t, *J* = 7.8 Hz, 2H), 3.08 (t, *J* = 7.5 Hz, 2H), 1.90–1.80 (m, 2H), 1.74–1.64 (m, 2H), 1.42–1.32 (m, 4H), 1.32–1.23 (m, 8H), 0.87–0.83 (m, 6H); ^13^C­{^1^H} NMR (75 MHz, CDCl_3_) δ 62.6, 36.2, 31.2, 31.1, 29.6, 28.2, 27.6, 23.5, 22.4, 22.3_4_, 22.2_7_, 13.9. The spectroscopic data were consistent with the reported values.^12b^

#### S-Octyl Octane-1-sulfonothioate (**2s**)

Sodium nitrate (8.5 mg, 0.10 mmol) was added to a solution of octane-1-thiol (**1s**, 146.3 mg, 1.0 mmol) and trifluoroacetic acid (306 μL, 456.0 mg, 4.0 mmol) in dichloromethane (1.0 mL) at 25 °C. The resulting reaction mixture was stirred at 25 °C for 1 h under an oxygen atmosphere (O_2_ balloon). After completion, the mixture was filtered through a pad of Celite and eluted with dichloromethane (10 mL). The filtrate was added with water (10 mL) and extracted with dichloromethane (3 × 5 mL). The combined organic layers were dried over anhydrous sodium sulfate, filtered, and concentrated under reduced pressure. The crude product was purified by column chromatography (SiO_2_, EtOAc/hexanes, 1:9, *R*_*f*_ 0.71) to give compound **2s** (159.3 mg, 0.49 mmol, 98%) as a colorless liquid. ^1^H NMR (300 MHz, CDCl_3_) δ 3.27 (t, *J* = 7.8 Hz, 2H), 3.11 (t, *J* = 7.5 Hz, 2H), 1.94–1.84 (m, 2H), 1.77–1.67 (m, 2H), 1.41–1.39 (m, 4H), 1.26 (s, br, 16H), 0.89–0.84 (m, 6H); ^13^C­{^1^H} NMR (75 MHz, CDCl_3_) δ 62.8, 36.4, 31.8, 29.7, 29.2, 29.1, 29.0, 28.7, 28.1, 23.6, 22.7, 14.2. The spectroscopic data were consistent with the reported values.^12b^

#### S-Dodecyl Dodecane-1-sulfonothioate (**2t**)

Sodium nitrate (8.5 mg, 0.10 mmol) was added to a solution of dodecane-1-thiol (**1t**, 202.4 mg, 1.0 mmol) and trifluoroacetic acid (306 μL, 456.0 mg, 4.0 mmol) in dichloromethane (1.0 mL) at 25 °C. The resulting reaction mixture was stirred at 25 °C for 1 h under an oxygen atmosphere (O_2_ balloon). After completion, the mixture was filtered through a pad of Celite and eluted with dichloromethane (10 mL). The filtrate was added with water (10 mL) and extracted with dichloromethane (3 × 5 mL). The combined organic layers were dried over anhydrous sodium sulfate, filtered, and concentrated under reduced pressure. The crude product was purified by column chromatography (SiO_2_, EtOAc/hexanes, 1:9, *R*_*f*_ 0.74) to give compound **2t** (216.1 mg, 0.49 mmol, 98%) as a colorless liquid. ^1^H NMR (300 MHz, CDCl_3_) δ 3.28 (t, *J* = 7.8 Hz, 2H), 3.12 (t, *J* = 7.5 Hz, 2H), 1.95–1.84 (m, 2H), 1.77–1.68 (m, 2H), 1.42–1.40 (m, 3H), 1.25 (s, br, 33H), 0.89–0.85 (m, 6H); ^13^C­{^1^H} NMR (75 MHz, CDCl_3_) δ 62.8, 36.4, 32.0, 29.7, 29.6, 29.53, 29.46, 29.4, 29.2, 29.1, 28.7, 28.1, 23.6, 22.8, 14.2. The spectroscopic data were consistent with the reported values.^12b^

#### S-Isobutyl 2-Methylpropane-1-sulfonothioate (**2u**)

Sodium nitrate (8.5 mg, 0.10 mmol) was added to a solution of 2-methylpropane-1-thiol (**1u**, 90.2 mg, 1.0 mmol) and trifluoroacetic acid (306 μL, 456.0 mg, 4.0 mmol) in dichloromethane (1.0 mL) at 25 °C. The resulting reaction mixture was stirred at 25 °C for 1 h under an oxygen atmosphere (O_2_ balloon). After completion, the mixture was filtered through a pad of Celite and eluted with dichloromethane (10 mL). The filtrate was added with water (10 mL) and extracted with dichloromethane (3 × 5 mL). The combined organic layers were dried over anhydrous sodium sulfate, filtered, and concentrated under reduced pressure. The crude product was purified by column chromatography (SiO_2_, EtOAc/hexanes, 1:9, *R*_*f*_ 0.66) to give compound **2u** (102.9 mg, 0.49 mmol, 98%) as a colorless liquid. ^1^H NMR (300 MHz, CDCl_3_) δ 3.20 (d, *J* = 6.4 Hz, 2H), 3.01 (d, *J* = 6.9 Hz, 2H), 2.45–2.31 (m, 1H), 2.22–1.91 (m, 1H), 1.10 (d, *J* = 6.9 Hz, 6H), 1.01 (d, *J* = 6.6 Hz, 6H); ^13^C­{^1^H} NMR (75 MHz, CDCl_3_) δ 70.5, 44.6, 28.9, 25.2, 22.5, 21.7. The spectroscopic data were consistent with the reported values.^40^

#### S-Isopropyl Propane-2-sulfonothioate (**2v**)

Sodium nitrate (8.5 mg, 0.10 mmol) was added to a solution of 2-propanethiol (**1v**, 76.2 mg, 1.0 mmol) and trifluoroacetic acid (306 μL, 456.0 mg, 4.0 mmol) in dichloromethane (1.0 mL) at 25 °C. The resulting reaction mixture was stirred at 25 °C for 1 h under an oxygen atmosphere (O_2_ balloon). After completion, the mixture was filtered through a pad of Celite and eluted with dichloromethane (10 mL). The filtrate was added with water (10 mL) and extracted with dichloromethane (3 × 5 mL). The combined organic layers were dried over anhydrous sodium sulfate, filtered, and concentrated under reduced pressure. The crude product was purified by column chromatography (SiO_2_, EtOAc/hexanes, 1:9, *R*_*f*_ 0.54) to give compound **2v** (87.5 mg, 0.48 mmol, 96%) as a colorless liquid. ^1^H NMR (300 MHz, CDCl_3_) δ 3.67–3.53 (m, 1H), 3.37–3.24 (m, 1H), 1.41–1.38 (m, 12H); ^13^C­{^1^H} NMR (75 MHz, CDCl_3_) δ 63.5, 43.0, 24.3, 16.3. The spectroscopic data were consistent with the reported values.^11d^

#### S-Cyclohexyl Cyclohexane­sulfonothioate (**2w**)

Sodium nitrate (8.5 mg, 0.10 mmol) was added to a solution of cyclohexanethiol (**1w**, 116.2 mg, 1.0 mmol) and trifluoroacetic acid (306 μL, 456.0 mg, 4.0 mmol) in dichloromethane (1.0 mL) at 25 °C. The resulting reaction mixture was stirred at 25 °C for 1 h under an oxygen atmosphere (O_2_ balloon). After completion, the mixture was filtered through a pad of Celite and eluted with dichloromethane (10 mL). The filtrate was added with water (10 mL) and extracted with dichloromethane (3 × 5 mL). The combined organic layers were dried over anhydrous sodium sulfate, filtered, and concentrated under reduced pressure. The crude product was purified by column chromatography (SiO_2_, EtOAc/hexanes, 1:9, *R*_*f*_ 0.42) to give compound **2w** (129.7 mg, 0.49 mmol, 98%) as a colorless liquid. ^1^H NMR (300 MHz, CDCl_3_) δ 3.45–3.37 (m, 1H), 3.07–2.97 (m, 1H), 1.95–1.84 (m, 2H), 2.25 (d, *J* = 11.7 Hz, 2H), 2.07–2.03 (m, 2H), 1.69–1.66 (m, 3H), 1.61–1.45 (m, 5H), 1.41–1.33 (m, 2H), 1.29–1.13 (m, 4H); ^13^C­{^1^H} NMR (75 MHz, CDCl_3_) δ 71.4, 50.4, 34.2, 26.2, 25.9, 25.2, 25.13, 25.06. The spectroscopic data were consistent with the reported values.^11d^

#### 1,2-Diphenyldisulfane (**3a**)

Sodium nitrate (8.5 mg, 0.10 mmol) was added to a solution of thiophenol (**1a**, 110.2 mg, 1.0 mmol) and trifluoroacetic acid (153 μL, 228.0 mg, 2.0 mmol) in dichloromethane (1.0 mL) at 25 °C. The resulting reaction mixture was stirred at 25 °C for 30 min under an oxygen atmosphere (O_2_ balloon). After completion, the mixture was filtered through a pad of Celite and eluted with dichloromethane (10 mL). The filtrate was added with water (10 mL) and extracted with dichloromethane (3 × 5 mL). The combined organic layers were dried over anhydrous sodium sulfate, filtered, and concentrated under reduced pressure. The crude product was purified by column chromatography (SiO_2_, EtOAc/hexanes, 1:9, *R*_*f*_ 0.64) to give compound **3a** (107.0 mg, 0.49 mmol, 98%) as a colorless solid. Mp 58.0–60.0 °C; ^1^H NMR (300 MHz, CDCl_3_) δ 7.58–7.56 (m, 4H), 7.41–7.34 (m, 4H), 7.31–7.28 (m, 2H); ^13^C­{^1^H} NMR (75 MHz, CDCl_3_) δ137.1, 129.2, 127.6, 127.3. The spectroscopic data were consistent with the reported values.^41^

#### 1,2-Di-p-tolyldisulfane (**3b**)

Sodium nitrate (8.5 mg, 0.10 mmol) was added to a solution of 4-methylbenzenethiol (**1b**, 124.2 mg, 1.0 mmol) and trifluoroacetic acid (76 μL, 114.0 mg, 1.0 mmol) in dichloromethane (1.0 mL) at 25 °C. The resulting reaction mixture was stirred at 25 °C for 1 h under an oxygen atmosphere (O_2_ balloon). After completion, the mixture was filtered through a pad of Celite and eluted with dichloromethane (10 mL). The filtrate was added with water (10 mL) and extracted with dichloromethane (3 × 5 mL). The combined organic layers were dried over anhydrous sodium sulfate, filtered, and concentrated under reduced pressure. The crude product was purified by column chromatography (SiO_2_, hexanes, *R*_*f*_ 0.78) to give compound **3b** (119.4 mg, 0.49 mmol, 97%) as a colorless solid. Mp 46.0–48.0 °C; ^1^H NMR (300 MHz, CDCl_3_) δ 7.46 (d, *J* = 8.1 Hz, 4H), 7.16 (d, *J* = 7.8 Hz, 4H); ^13^C­{^1^H} NMR (75 MHz, CDCl_3_) δ 137.5, 134.0, 129.9, 128.6, 21.1. The spectroscopic data were consistent with the reported values.^11d^

#### 1,2-Bis­(4-methoxyphenyl)­disulfane (**3g**)

Sodium nitrate (8.5 mg, 0.10 mmol) was added to a solution of 4-methoxybenzenethiol (**1g**, 140.2 mg, 1.0 mmol) and trifluoroacetic acid (76 μL, 114.0 mg, 1.0 mmol) in dichloromethane (1.0 mL) at 25 °C. The resulting reaction mixture was stirred at 25 °C for 3 h under an oxygen atmosphere (O_2_ balloon). After completion, the mixture was filtered through a pad of Celite and eluted with dichloromethane (10 mL). The filtrate was added with water (10 mL) and extracted with dichloromethane (3 × 5 mL). The combined organic layers were dried over anhydrous sodium sulfate, filtered, and concentrated under reduced pressure. The crude product was purified by column chromatography (SiO_2_, EtOAc/hexanes, 1:9, *R*_*f*_ 0.48) to give compound **3g** (136.6 mg, 0.49 mmol, 98%) as a colorless solid. Mp 42.0–44.0 °C; ^1^H NMR (300 MHz, CDCl_3_) δ 7.27 (d, *J* = 8.7 Hz, 4H), 6.81 (d, *J* = 9.0 Hz, 4H), 3.78 (s, 6H); ^13^C­{^1^H} NMR (75 MHz, CDCl_3_) δ 158.6, 132.5, 119.9, 114.8, 55.4. The spectroscopic data were consistent with the reported values.^42^

#### 1,2-Bis­(4-fluorophenyl)­disulfane (**3i**)

Sodium nitrate (8.5 mg, 0.10 mmol) was added to a solution of 4-fluorobenzenethiol (**1i**, 128.2 mg, 1.0 mmol) and trifluoroacetic acid (76 μL, 114.0 mg, 1.0 mmol) in dichloromethane (1.0 mL) at 25 °C. The resulting reaction mixture was stirred at 25 °C for 3 h under an oxygen atmosphere (O_2_ balloon). After completion, the mixture was filtered through a pad of Celite and eluted with dichloromethane (10 mL). The filtrate was added with water (10 mL) and extracted with dichloromethane (3 × 5 mL). The combined organic layers were dried over anhydrous sodium sulfate, filtered, and concentrated under reduced pressure. The crude product was purified by column chromatography (SiO_2_, EtOAc/hexanes, 1:9, *R*_*f*_ 0.58) to give compound **3i** (124.7 mg, 0.49 mmol, 98%) as a colorless liquid. ^1^H NMR (500 MHz, CDCl_3_) δ 7.46 (dd, *J* = 8.5, 5.0 Hz, 4H), 7.02 (t, *J* = 8.5 Hz 4H); ^13^C­{^1^H} NMR (126 MHz, CDCl_3_) δ 162.7 (d, *J* = 248.2 Hz), 132.3, 131.4 (d, *J* = 8.8 Hz), 116.4 (d, *J* = 22.7 Hz); ^19^F-NMR (470 MHz, CDCl_3_) δ −113.3. The spectroscopic data were consistent with the reported values.^43^

#### 1,2-Bis­(4-nitrophenyl)­disulfane (**3y**)

Sodium nitrate (8.5 mg, 0.10 mmol) was added to a solution of 4-nitrobenzenethiol (**1y**, 155.2 mg, 1.0 mmol) and trifluoroacetic acid (76 μL, 114.0 mg, 1.0 mmol) in dichloromethane (1.0 mL) at 25 °C. The resulting reaction mixture was stirred at 25 °C for 3 h under an oxygen atmosphere (O_2_ balloon). After completion, the mixture was filtered through a pad of Celite and eluted with dichloromethane (10 mL). The filtrate was added with water (10 mL) and extracted with dichloromethane (3 × 5 mL). The combined organic layers were dried over anhydrous sodium sulfate, filtered, and concentrated under reduced pressure. The crude product was purified by column chromatography (SiO_2_, EtOAc/hexanes, 1:9, *R*_*f*_ 0.42) to give compound **3i** (152.9 mg, 0.49 mmol, 98%) as a colorless solid. Mp 182.0–184.0 °C; ^1^H NMR (300 MHz, CDCl_3_) δ 8.19 (d, *J* = 9.0 Hz, 4H), 7.61 (d, *J* = 9.0 Hz, 4H); ^13^C­{^1^H} NMR (75 MHz, CDCl_3_) δ 147.0, 144.2, 126.5, 124.6. The spectroscopic data were consistent with the reported values.^44^

#### 1,2-Didodecyldisulfane (**3t**)

Sodium nitrate (8.5 mg, 0.10 mmol) was added to a solution of 1-dodecanethiol (**1t**, 202.4 mg, 1.0 mmol) and trifluoroacetic acid (230 μL, 342.0 mg, 3.0 mmol) in dichloromethane (1.0 mL) at 25 °C. The resulting reaction mixture was stirred at 25 °C for 3 h under an oxygen atmosphere (O_2_ balloon). After completion, the mixture was filtered through a pad of Celite and eluted with dichloromethane (10 mL). The filtrate was added with water (10 mL) and extracted with dichloromethane (3 × 5 mL). The combined organic layers were dried over anhydrous sodium sulfate, filtered, and concentrated under reduced pressure. The crude product was purified by column chromatography (SiO_2_, hexanes, *R*_*f*_ 0.9) to give compound **3t** (199.4 mg, 0.49 mmol, 98%) as a colorless liquid. ^1^H NMR (300 MHz, CDCl_3_) δ 2.69–2.64 (m, 2H), 2.55–2.47 (m, 1H), 1.71–1.55 (m, 4H), 1.39–1.26 (m, 37H), 0.90–0.85 (m, 6H); ^13^C­{^1^H} NMR (75 MHz, CDCl_3_) δ 39.3, 34.2, 32.1, 29.8, 29.7, 29.5, 29.4, 29.2, 28.7, 24.8, 22.8, 14.2. The spectroscopic data were consistent with the reported values.^43^

#### 1,2-Di­(pyridin-2-yl)­disulfane (**3z**)

Sodium nitrate (8.5 mg, 0.10 mmol) was added to a solution of pyridine-2-thiol (**1z**, 111.2 mg, 1.0 mmol) and trifluoroacetic acid (306 μL, 456.0 mg, 4.0 mmol) in dichloromethane (1.0 mL) at 25 °C. The resulting reaction mixture was stirred at 25 °C for 16 h under an oxygen atmosphere (O_2_ balloon). After completion, the mixture was filtered through a pad of Celite and eluted with dichloromethane (10 mL). The filtrate was added with water (10 mL) and extracted with dichloromethane (3 × 5 mL). The combined organic layers were dried over anhydrous sodium sulfate, filtered, and concentrated under reduced pressure. The crude product was purified by column chromatography (SiO_2_, EtOAc/hexanes, 1:1, *R*_*f*_ 0.33) to give compound **3z** (104.6 mg, 0.47 mmol, 95%) as a colorless liquid. ^1^H NMR (300 MHz, CDCl_3_) δ 8.38–8.37 (m, 2H), 7.53–7.51 (m, 4H), 7.05–7.00 (m, 2H); ^13^C­{^1^H} NMR (75 MHz, CDCl_3_) δ 158.7, 149.4, 137.4, 121.1, 119.6. The spectroscopic data were consistent with the reported values.^45^

#### Oxidized Glutathione (GSSG, **5**)

Sodium nitrate (8.5 mg, 0.10 mmol) was added to a solution of glutathione (**4**, 307.3 mg, 1.0 mmol) and trifluoroacetic acid (460 μL, 684 mg, 6.0 mmol) in dichloromethane (1.0 mL) at 25 °C. The resulting reaction mixture was stirred at 25 °C for 1 h under an oxygen atmosphere (O_2_ balloon), and the solvent was removed under reduced pressure. The residue was washed with ethyl acetate (3 × 5 mL) and hexanes (3 × 5 mL) and concentrated to yield **5** (301.8 mg, 0.493 mmol, 98%) as a colorless solid. Mp 174.0–176.0 °C; ^1^H NMR (300 MHz, D_2_O) δ 4.63–4.59 (m, 2H), 3.99–3.94 (m, 3H), 3.88 (s, br, 4H), 3.17–3.09 (m, 2H), 2.89–2.81 (m, 1H), 2.52–2.46 (m, 4H), 2.20–2.06 (m, 4H); ^13^C­{^1^H} NMR (75 MHz, D_2_O) δ174.2, 172.7, 172.4, 171.3, 52.5, 52.1, 41.1, 38.6, 30.8, 25.3; HRMS (ESI) *m*/*z* calcd for [M + Na]^+^ (C_20_H_32_N_6_O_12_S_2_Na) 635.1411, found 635.1405; The spectroscopic data were consistent with the reported values.^46^

## Supplementary Material





## Data Availability

The data underlying this study are available in the published article and its .

## Notes

Oxidation of thiol **1a** conducted in various solvents, p*K*_a_ values of the acids used in this work, figures of starch-iodide test, and copies of ^1^H and ^13^C­{^1^H} NMR spectra for all synthesized compounds (PDF)
FAIR data, including the primary NMR FID files, for compounds **2a**–**2w**, **3a**–**3b**, **3g**, **3i**, **3t**, **3x**–**3z** and **5** (ZIP)
