# Supplementary material for: A Stepwise, Nitrosonium-Catalyzed Aerobic Oxidation of Thiols to Disulfides and Thiosulfonates
Source: J Org Chem. 2026 Mar 20;91(13):4755–66. doi: 10.1021/acs.joc.6c00062 (PMC13054865; doi:10.1021/acs.joc.6c00062)
Supplement: Supplementary file 1 [file jo6c00062_si_001.pdf]

Supporting Information

A Stepwise, Nitrosonium-Catalyzed Aerobic Oxidation of Thiols to Disulfides and Thiosulfonates.

Mallepalli Shankar and Duen-Ren Hou*

Department of Chemistry, National Central University, No. 300 Jhong-Da Rd., Jhong-li, Taoyuan, Taiwan, 320317.

Table of Contents

Table S1. pK_a Values of the acids used in this work……………………………........S5

Table S2. Oxidation of thiol **1a** conducted in various solvents……………………........S6

Figure S1. Starch-Iodide Test ……………………………………………………………S7

^{1}H NMR of compound **2a** …...….........…...............................….............................S8

^{13}C {^{1}H} NMR of compound **2a**.......…..S9

^{1}H NMR of compound **2b**..........….........…..S10

^{13}C {^{1}H} NMR of compound **2b.**...........……..S11

^{1}H NMR of compound **2c**..........…............…..S12

^{13}C {^{1}H} NMR of compound **2c**……..S13

^{1}H NMR of compound **2d**..…....…........….............…...S14

^{13}C {^{1}H} NMR of compound **2d**..……..…….....……S15

^{1}H NMR of compound **2e**…......…........…....................…...S16

^{13}C {^{1}H} NMR of compound **2e**…........……...….……….S17

^{1}H NMR of compound **2f**….......…........…....................…...S18

^{13}C {^{1}H} NMR of compound **2f**…......…...……….…..S19

^{1}H NMR of compound **2g**….......…........…....................…...S20

^{13}C {^{1}H} NMR of compound **2g**….......…...….……S21

^{1}H NMR of compound **2h**…......….........…...................…...S22

^{13}C {^{1}H} NMR of compound **2h**…..…………S23

^{1}H NMR of compound **2i**……....…........…....................…..S24

^{13}C {^{1}H} NMR of compound **2i**........……..…….…….…S25

^{19}F {^{1}H} NMR of compound **2i**..............…..S26

^{1}H NMR of compound **2j**……...……..........……...................….....................................S27
^{13}C {^{1}H} NMR of compound **2j**...……………..S28
^{1}H NMR of compound **2k**……...……..........……...................….....................................S29
^{13}C {^{1}H} NMR of compound **2k**.........………...…..………….S30
^{1}H NMR of compound **2l**……...……..........……...................….....................................S31
^{13}C {^{1}H} NMR of compound **2l**.........………...…………S32
^{1}H NMR of compound **2m**……...……..........……...................….....................................S33
^{13}C {^{1}H} NMR of compound **2m**....…...…………S34
^{1}H NMR of compound **2n**.........…...................…..S35
^{13}C {^{1}H} NMR of compound **2n**...……...…...................S36
^{1}H NMR of compound **2o**………...……..........……...................….....................................S37
^{13}C {^{1}H} NMR of compound **2o**..……………...S38
^{1}H NMR of compound **2p**……...……..........……...................….....................................S39
^{13}C {^{1}H} NMR of compound **2p**..…………S40
^{1}H NMR of compound **2q**……...……..........……...................….....................................S41
^{13}C {^{1}H} NMR of compound **2q**.........………..…………S42
^{1}H NMR of compound **2r**………...……..........……...................….....................................S43
^{13}C {^{1}H} NMR of compound **2r**…..…………S44
^{1}H NMR of compound **2s**....……..........……...................….....................................S45
^{13}C {^{1}H} NMR of compound **2s**.........………...……………..S46
^{1}H NMR of compound **2t**……...……..........……...................….....................................S47
^{13}C {^{1}H} NMR of compound **2t**...……………..S48
^{1}H NMR of compound **2u**……...……..........……...................….....................................S49
^{13}C {^{1}H} NMR of compound **2u**...……………S50

^{1}H NMR of compound **2v**..S51

^{13}C {^{1}H} NMR of compound **2v**..S52

^{1}H NMR of compound **2w**..S53

^{13}C {^{1}H} NMR of compound **2w**..S54

^{1}H NMR of compound **3a**..S55

^{13}C {^{1}H} NMR of compound **3a**..S56

^{1}H NMR of compound **3b**..S57

^{13}C {^{1}H} NMR of compound **3b**..S58

^{1}H NMR of compound **3g**..S59

^{13}C {^{1}H} NMR of compound **3g**..S60

^{1}H NMR of compound **3i**..S61

^{13}C {^{1}H} NMR of compound **3i**..S62

^{19}F {^{1}H} NMR of compound **3i**..S63

^{1}H NMR of compound **3t**..S64

^{13}C {^{1}H} NMR of compound **3t**..S65

^{1}H NMR of compound **3x**..S66

^{13}C {^{1}H} NMR of compound **3x**..S67

^{1}H NMR of compound **3y**..S68

^{13}C {^{1}H} NMR of compound **3y**..S69

^{1}H NMR of compound **3z**..S70

^{13}C {^{1}H} NMR of compound **3z**..S71

^{1}H NMR of compound **5**..S72

^{13}C {^{1}H} NMR of compound **5**..S73

Table S1. pK_a Values of the acids used in this work.

entry	acid	pKa
1	H_2SO_4	-
2	HNO_3	-1.3[1]
3	HCl	-7[1]
4	HBr	-9.0[2]
5	HI	-10.0[2]
6	CF_3CO_2H	0.3[3]
7	CCl_3CO_2H	0.66[4]
8	CH_3CO_2H	4.7[4]
9	*p*-TsOH	-2.8 (reference for benzenesulfonic acid)[5]
10	CH_3SO_3H	-1.86[6]

References:

1. Bell, R. P., *The Proton in Chemistry*. Springer New York, 1973.
2. Brownstein, S.; Stillman, A. E., *J. Phys. Chem.* **1959,** *63*, 2061-2062.
3. Balasuryia, D.; Queral-Beltran, A.; Vick, T.; Simpson, S.; Lacorte, S.; Aga, D. S.; Hoepker, A. C., *Environ. Sci. Technol. Lett.* **2025,** *12*, 1238-1246.
4. *CRC Handbook of Chemistry and Physics*, 88th ed Editor-in-Chief: David R. Lide (National Institute of Standards and Technology) CRC Press/Taylor & Francis Group: Boca Raton, FL. 2007.
5. Guthrie, J. P., *Can. J. Chem.* **1978,** *56*, 2342-2354.
6. Vivenzio, G.; Noki, S.; Chakraborty, A.; Lopez, J.; de la Torre, B. G.; Albericio, F., *ChemSusChem* **2025,** *18*, e202402752.

Table S2. Oxidation of thiol **1a** conducted in various solvents.[a]

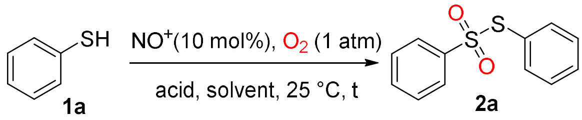

entry	NO^+ source	acid (equiv)	solvent	time (h)	yield (%)
1	$NaNO_3$	TFA (2.0)	CH_2Cl_2	2	99
2	$NaNO_3$	TFA (2.0)	toluene	3	87
3	$NaNO_3$	TFA (2.0)	$(CH_2Cl)_2$	1	98[b]
4	$NaNO_3$	TFA (2.0)	$CHCl_3$	3	-[c]
5	$NaNO_3$	TFA (2.0)	CH_3NO_2	24	0[d]
6	$NaNO_3$	TFA (2.0)	ethyl acetate	24	0[d]
7	$NaNO_3$	TFA (2.0)	CH_3CN	24	0[d]
8	$NaNO_3$	TFA (2.0)	MeOH	24	0[d]
9	$NaNO_3$	TFA (2.0)	EtOH	24	0[d]
10	$NaNO_3$	TFA (2.0)	CF_3CH_2OH	24	0[d]
11	$NaNO_3$	TFA (2.0)	THF	24	0[d]
12	$NaNO_3$	TFA (2.0)	DMF	24	0[d]
13	$NaNO_3$	TFA (2.0)	dioxane	24	0[d]

[a]A source of nitrosonium ion (0.1 mmol) was added to a solution of **1a** (110.2 mg, 1.0 mmol), TFA (153.1 μL, 1.0 mmol) and a solvent (1.0 mL) at 25 °C. The reaction mixture was stirred at 25 °C under an atmosphere of oxygen (balloon) for the time indicated, and then water (5 mL) was added. The products were obtained after extractions (5 mL ethyl acetate × 3), dried over Na_2SO_4, filtered through a Celite pad and concentrated.

[b]Diphenyl disulfide.

[c]Decomposed.

[d]Starting material **1a** recovered.

Starch-Iodide Test:

(a) A solution of thiophenol (110.2 mg, 1.0 mmol), TFA (153.1 µL, 2.0 mmol) and CH_2Cl_2 (1 mL) was prepared.

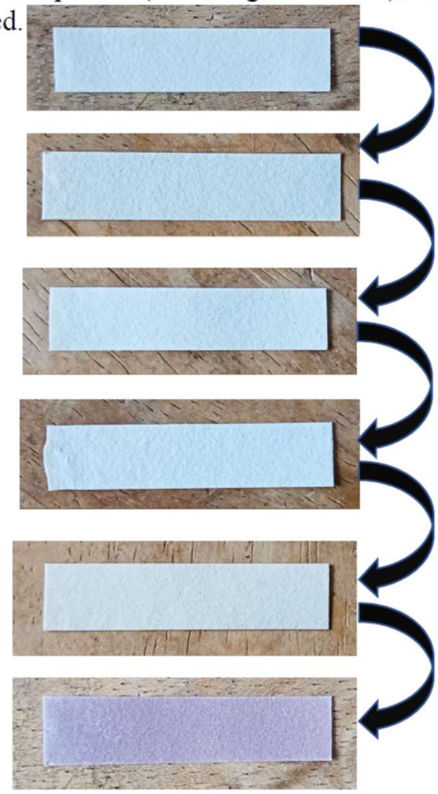

Figure S1. Starch-Iodide Test

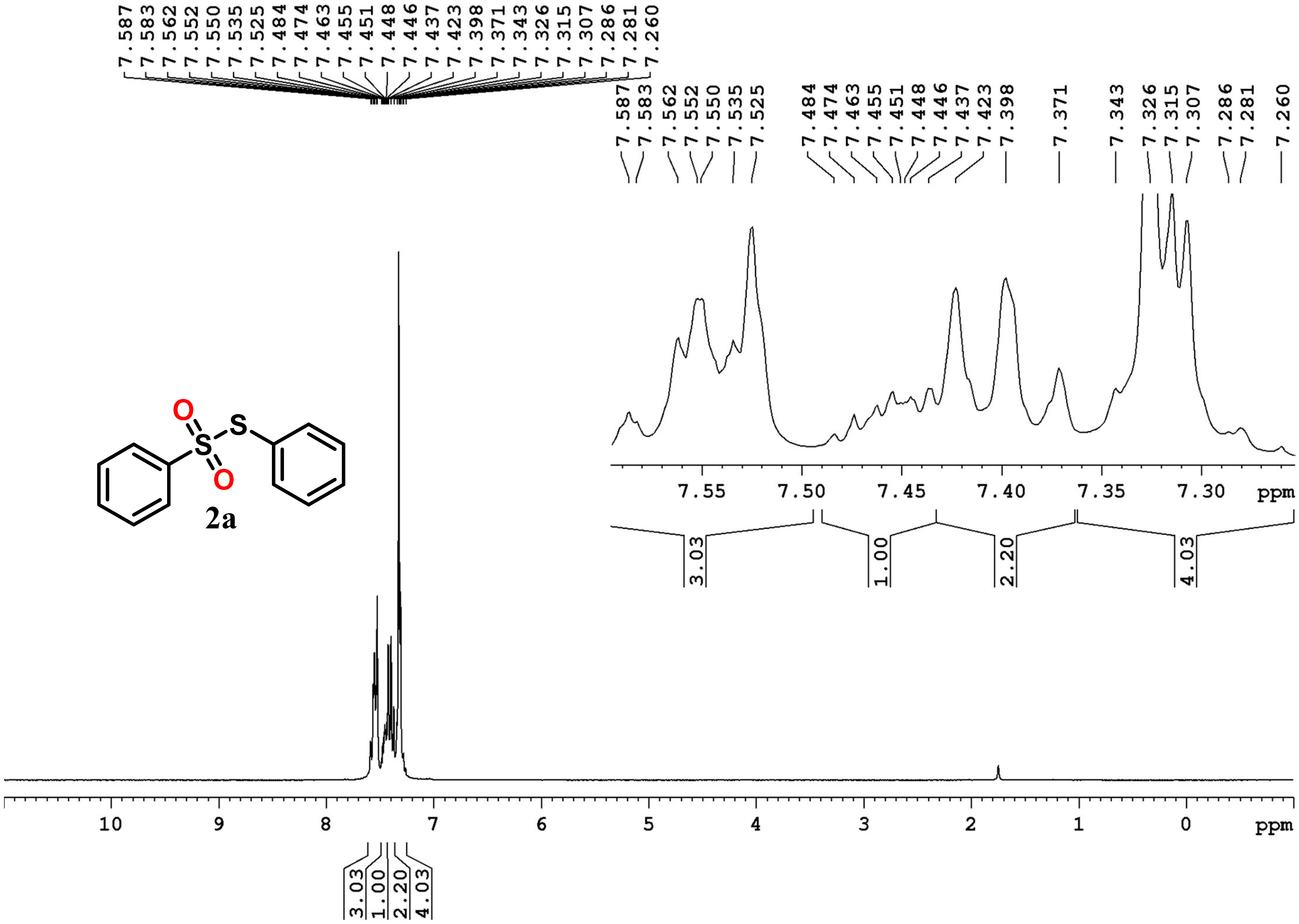

^1H NMR of compound **2a** (300 MHz, $CDCl_3$)

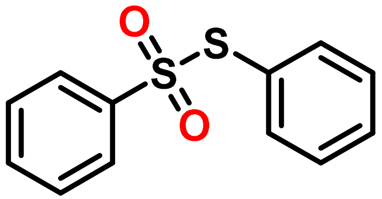

2a

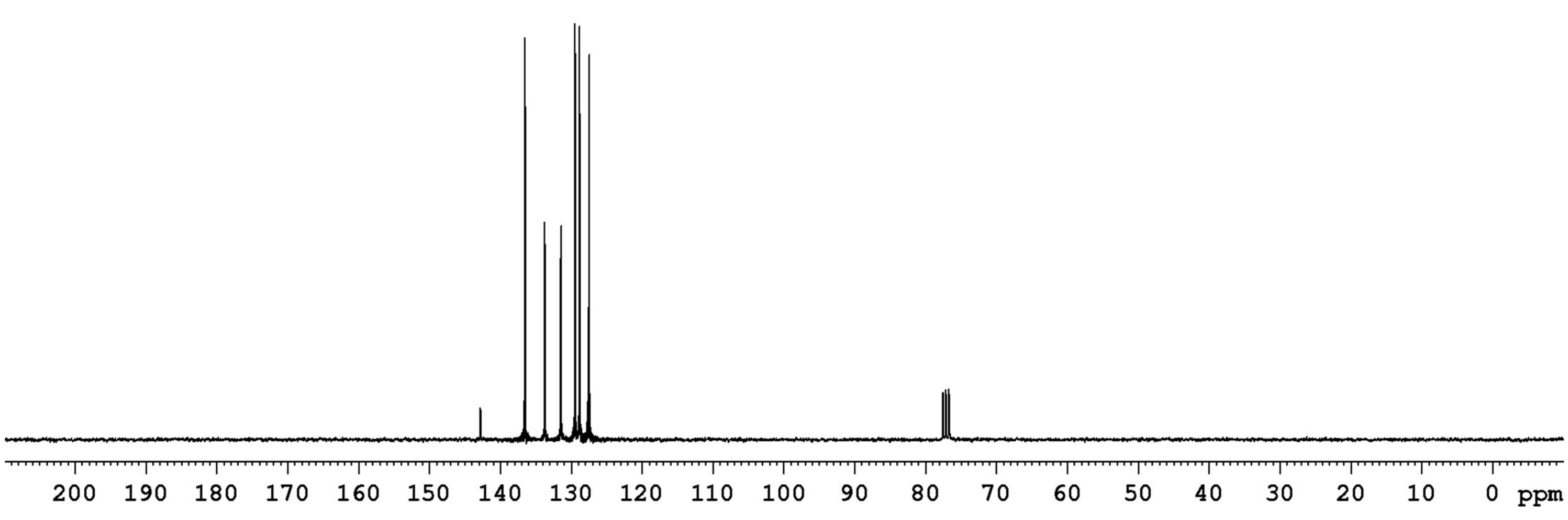

$^{13}C\ \{^{1}H\}$ NMR of compound **2a** (75 MHz, $CDCl_3$)

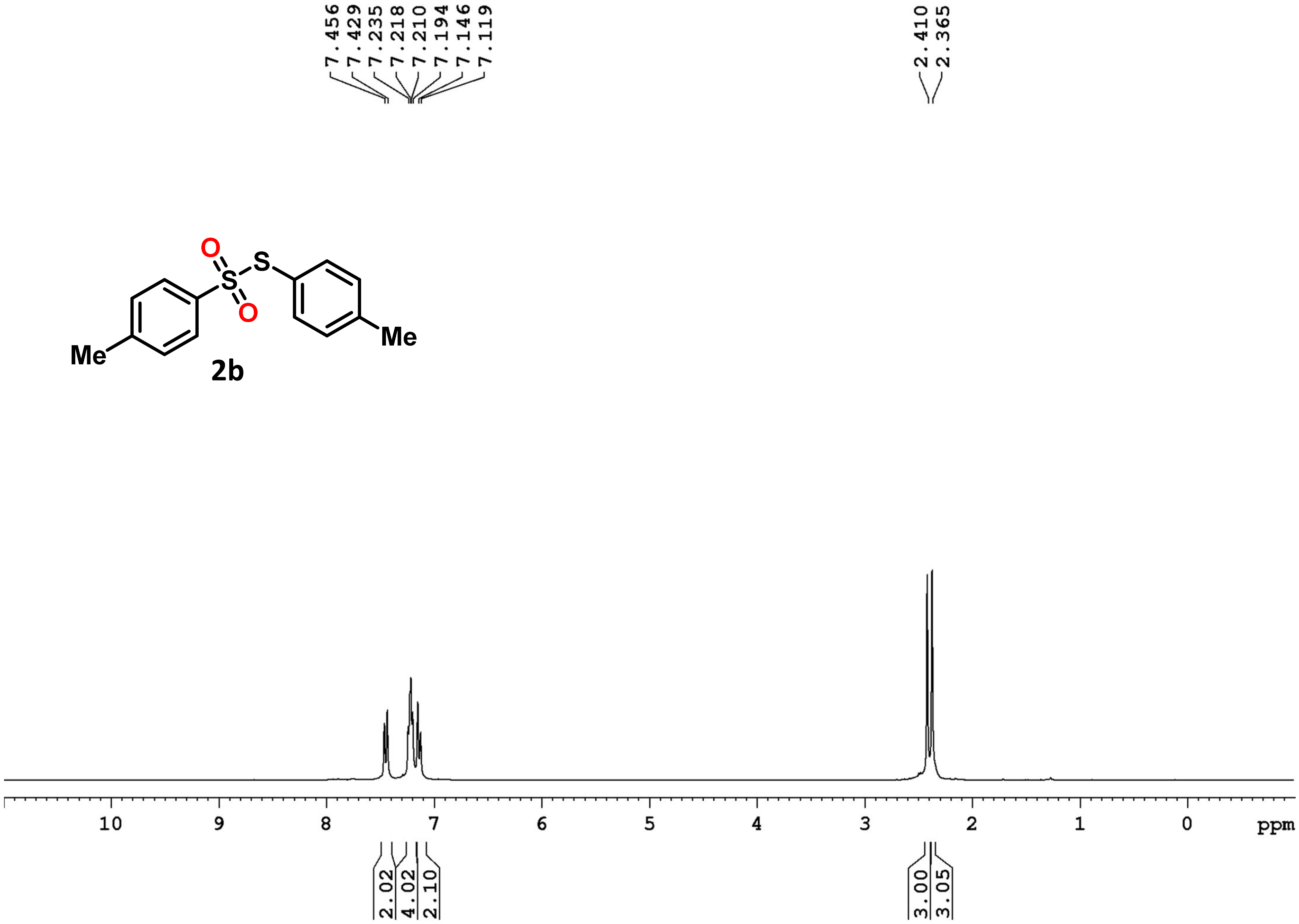

^{1}H NMR of compound **2b** (300 MHz, $CDCl_3$)

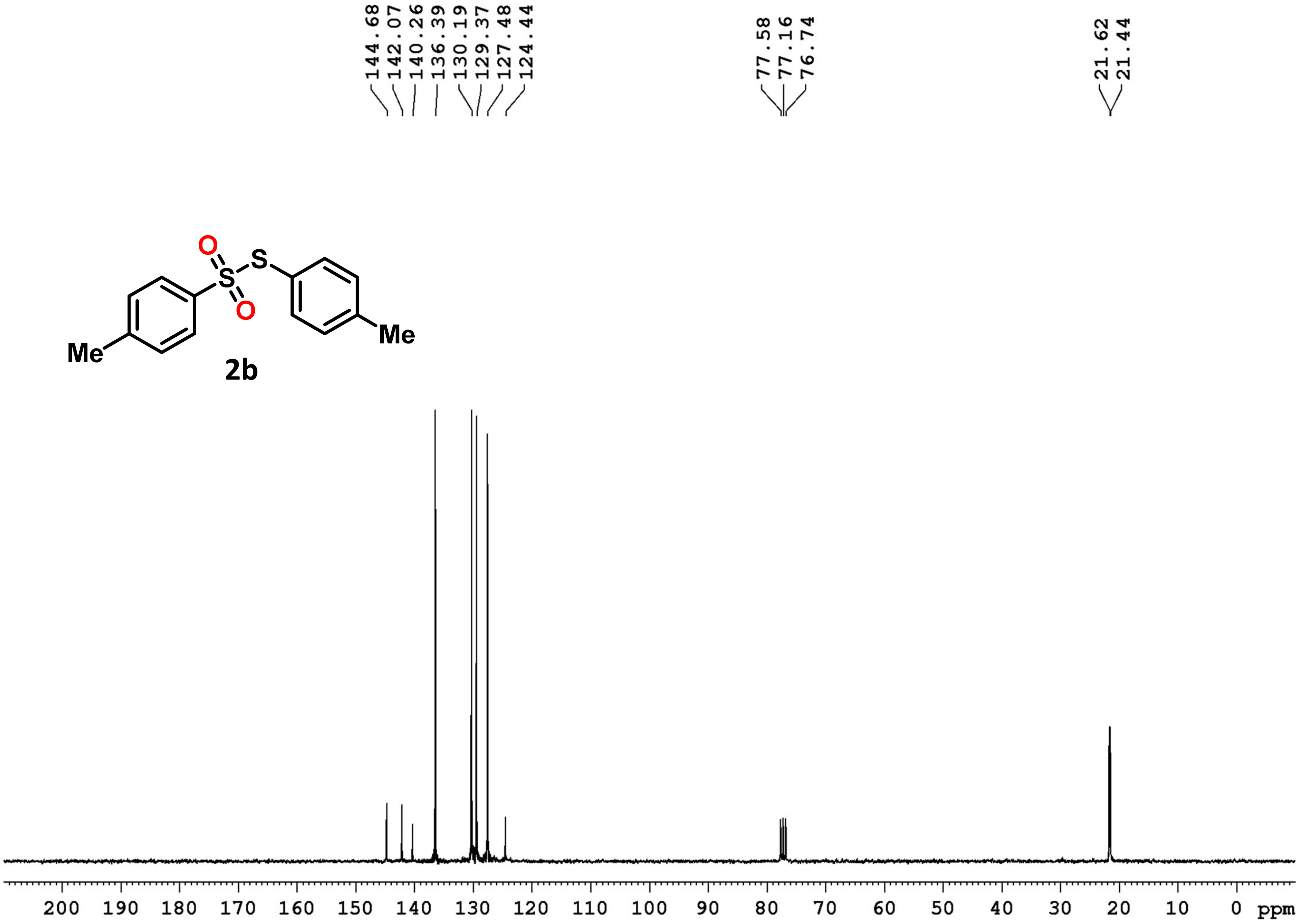

^{13}C {^{1}H} NMR of compound **2b** (75 MHz, $CDCl_3$)

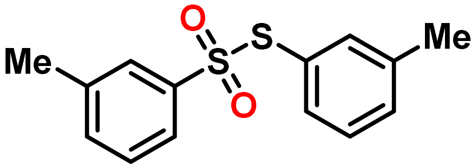

2c

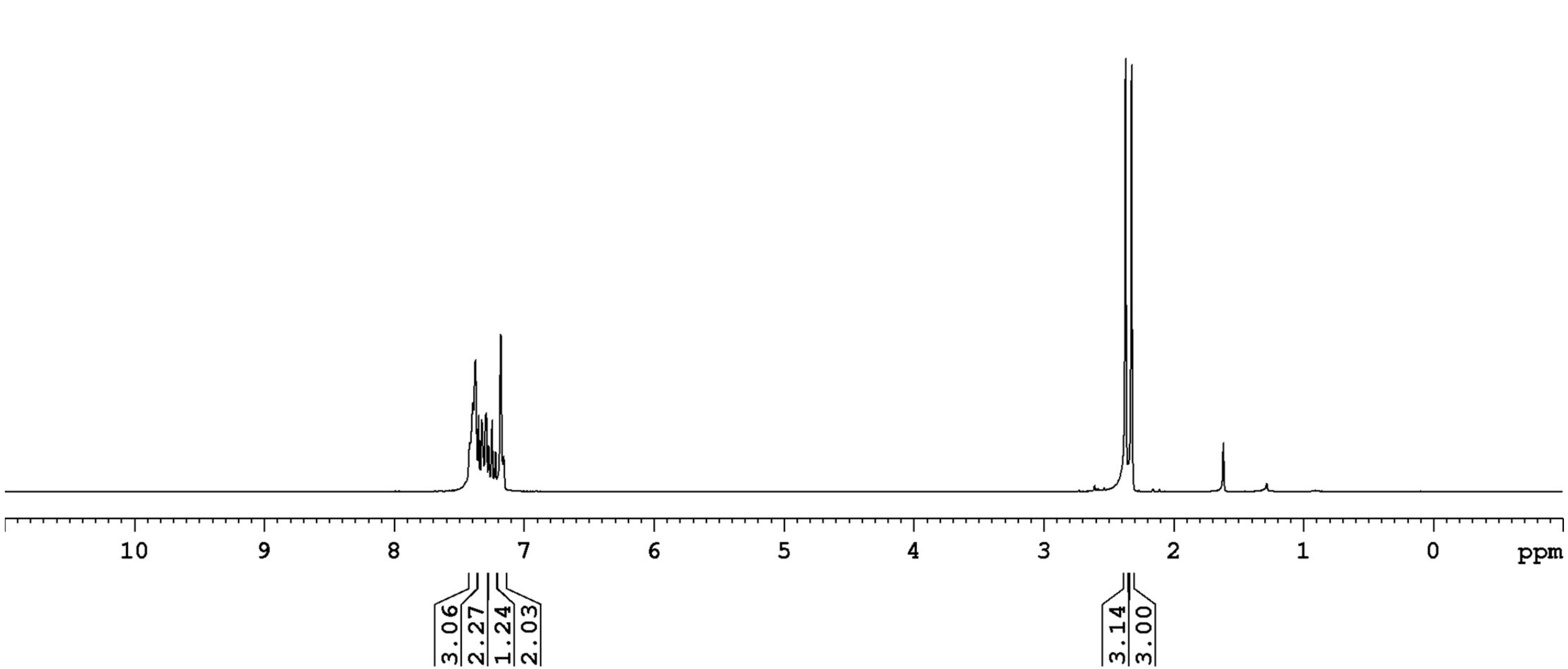

^{1}H NMR of compound **2c** (300 MHz, $CDCl_3$)

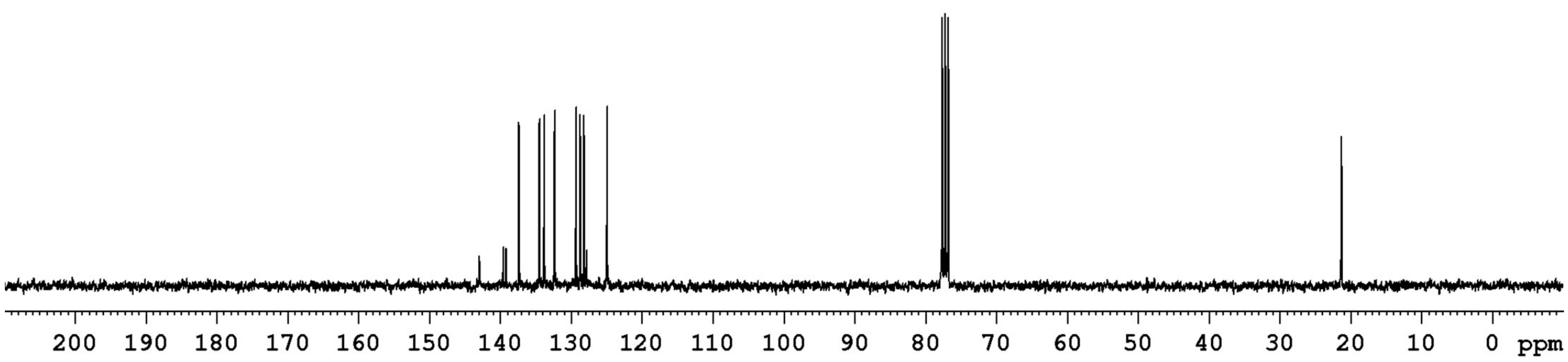

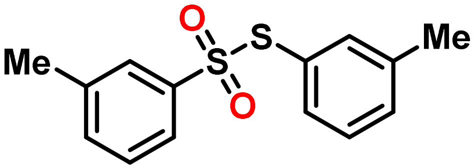

2c

^{13}C {^{1}H} NMR of compound **2c** (75 MHz, $CDCl_3$)

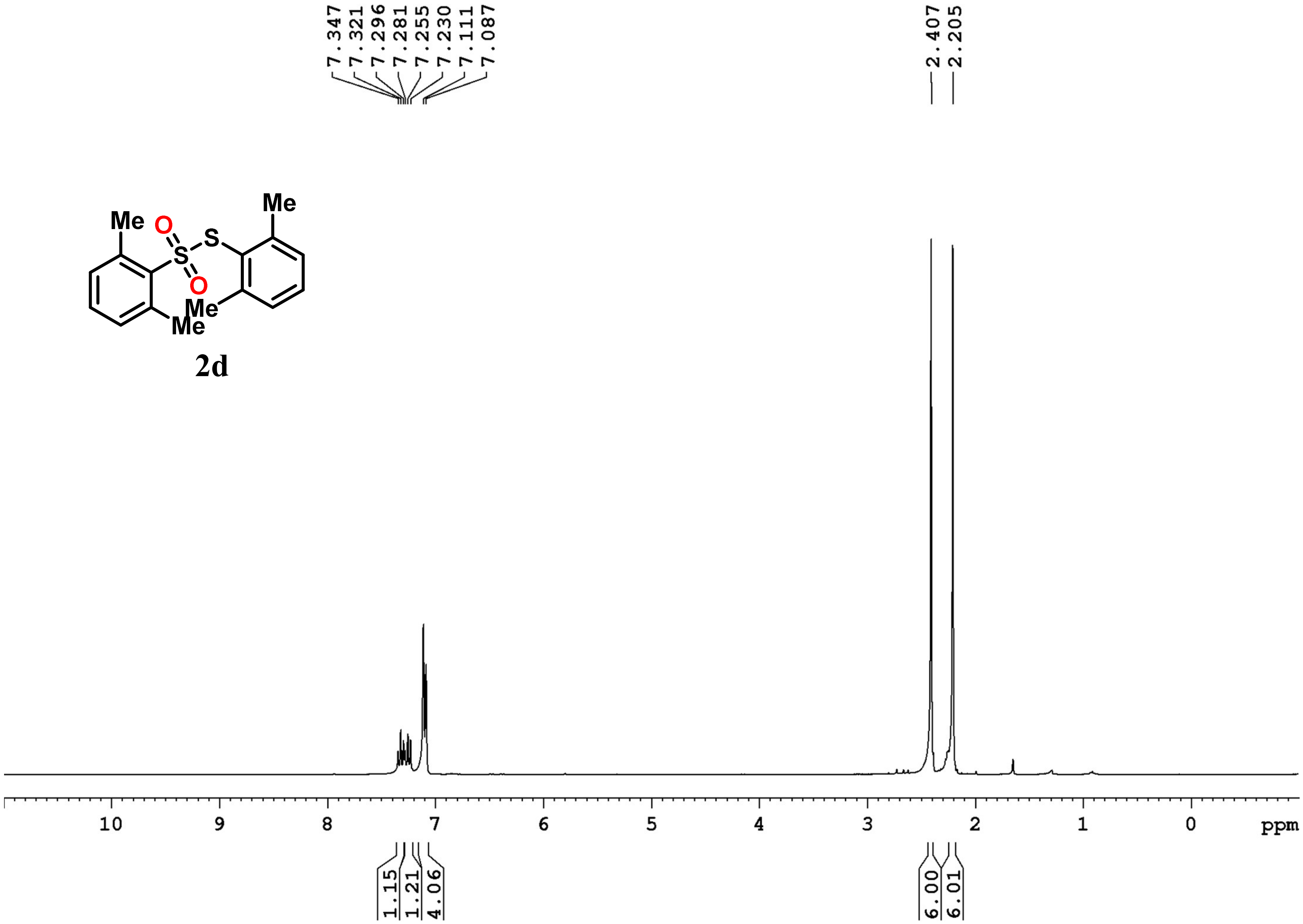

^{1}H NMR of compound **2d** (300 MHz, $CDCl_3$)

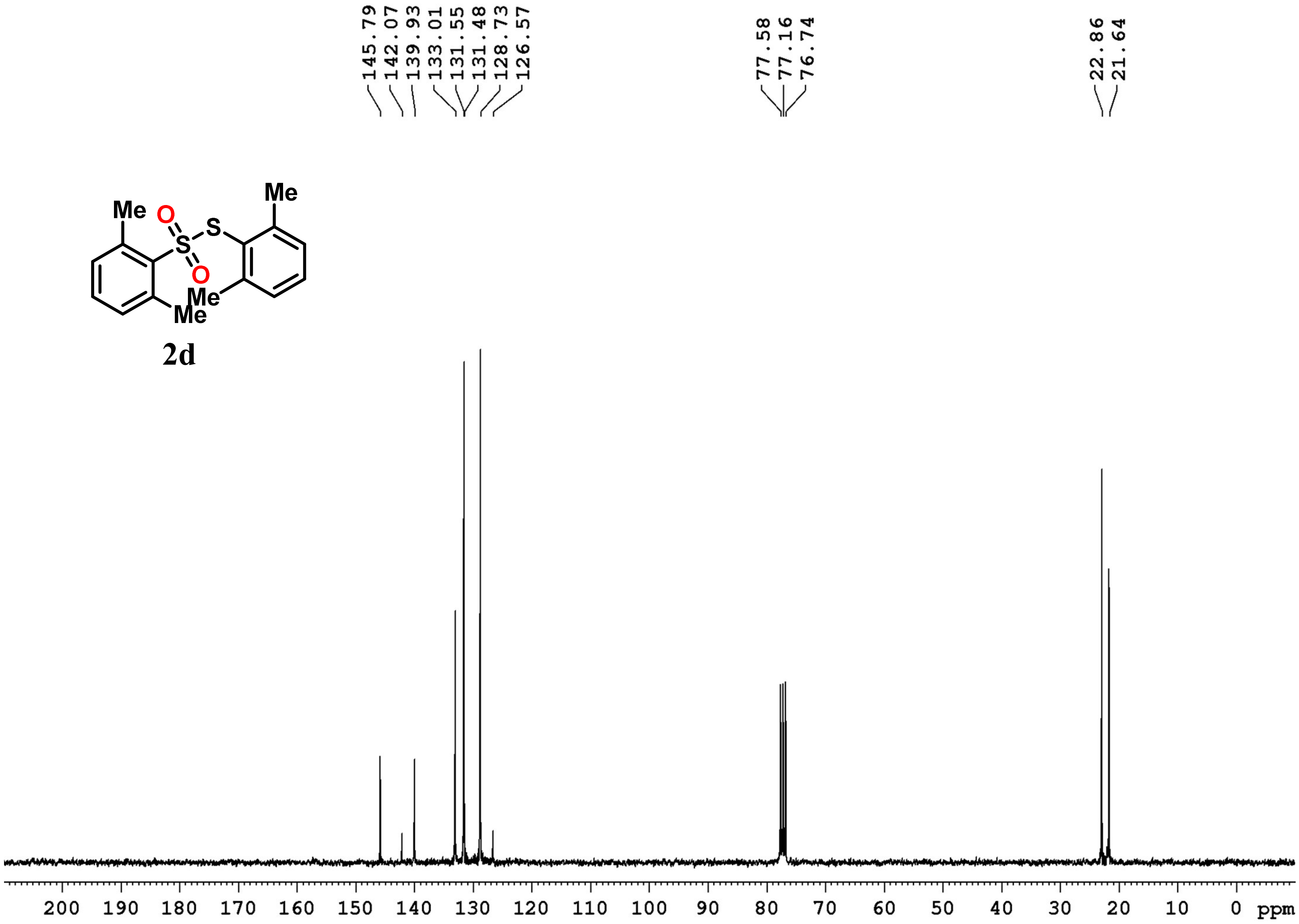

$^{13}C\ \{^{1}H\}$ NMR of compound **2d** (75 MHz, $CDCl_3$)

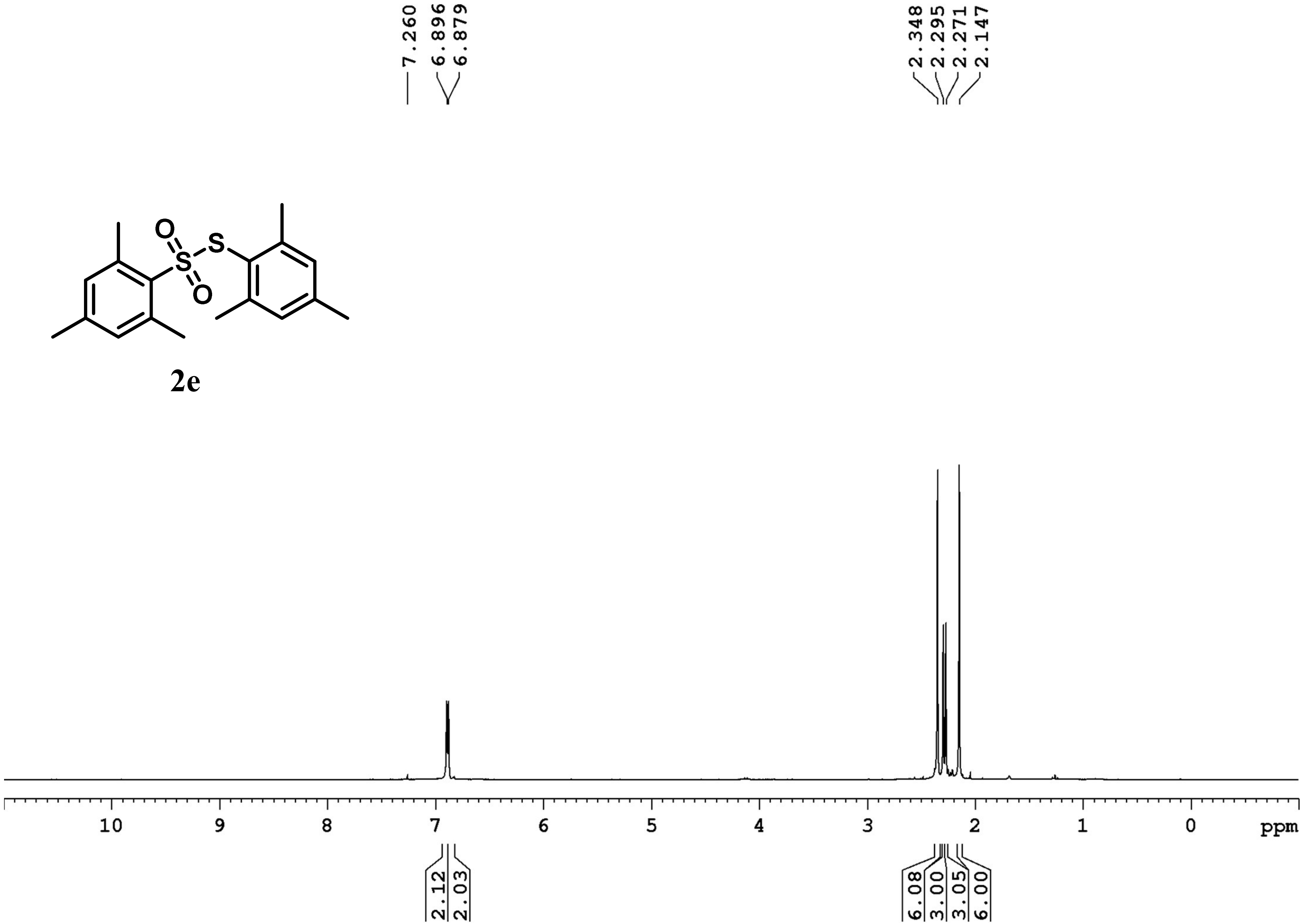

^{1}H NMR of compound **2e** (300 MHz, $CDCl_3$)

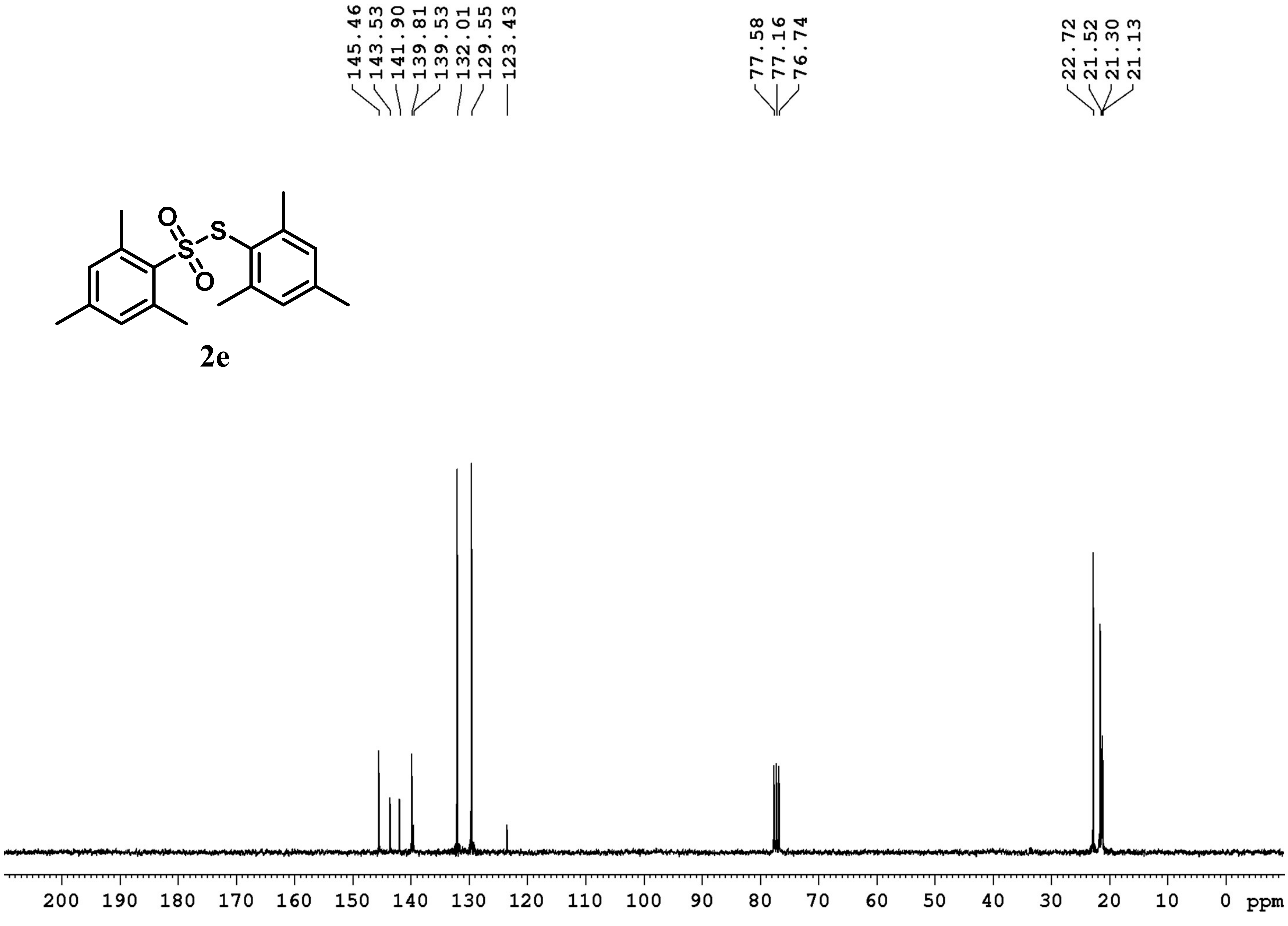

^{13}C {^{1}H} NMR of compound **2e** (75 MHz, $CDCl_3$)

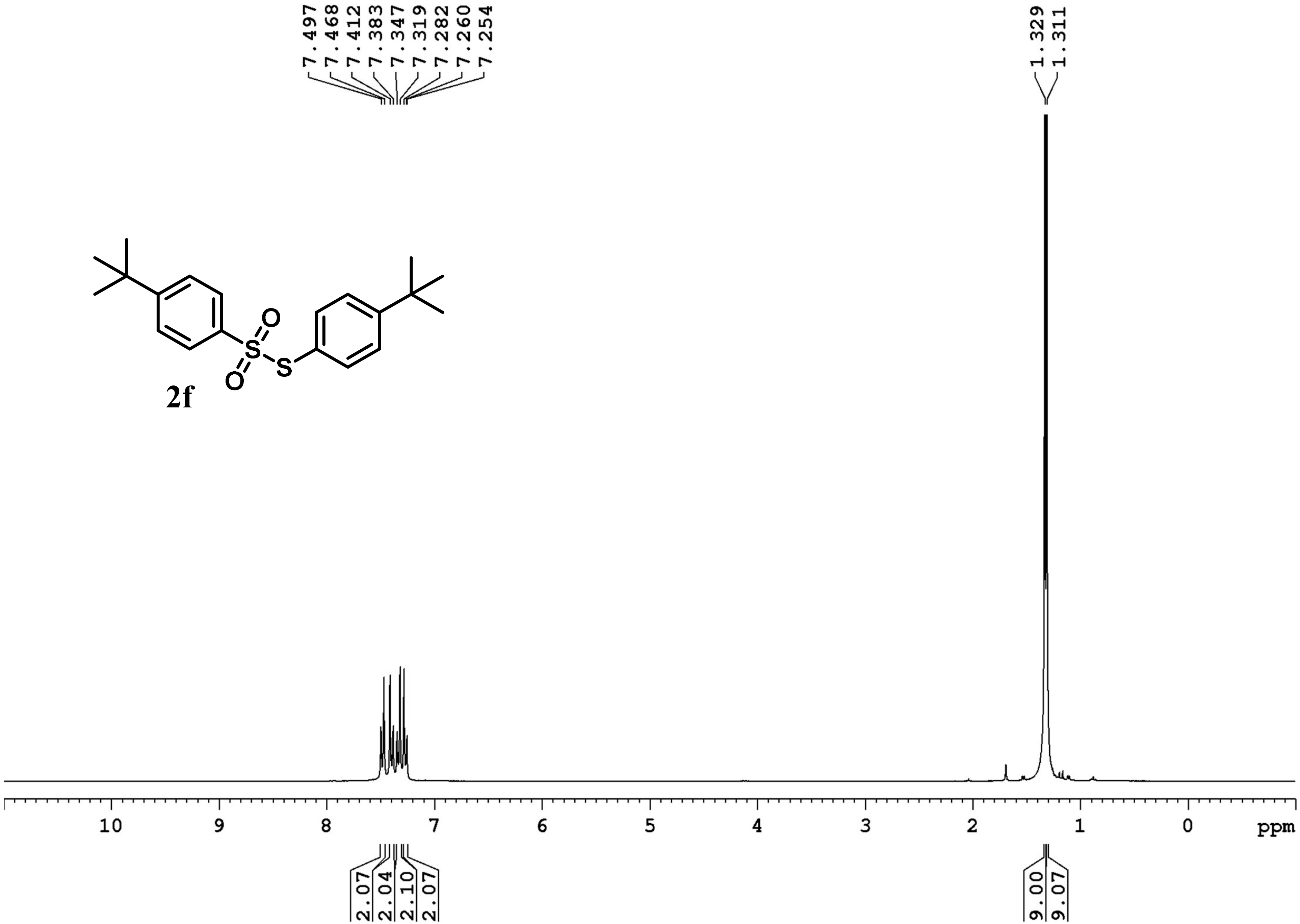

^{1}H NMR of compound **2f** (300 MHz, $CDCl_3$)

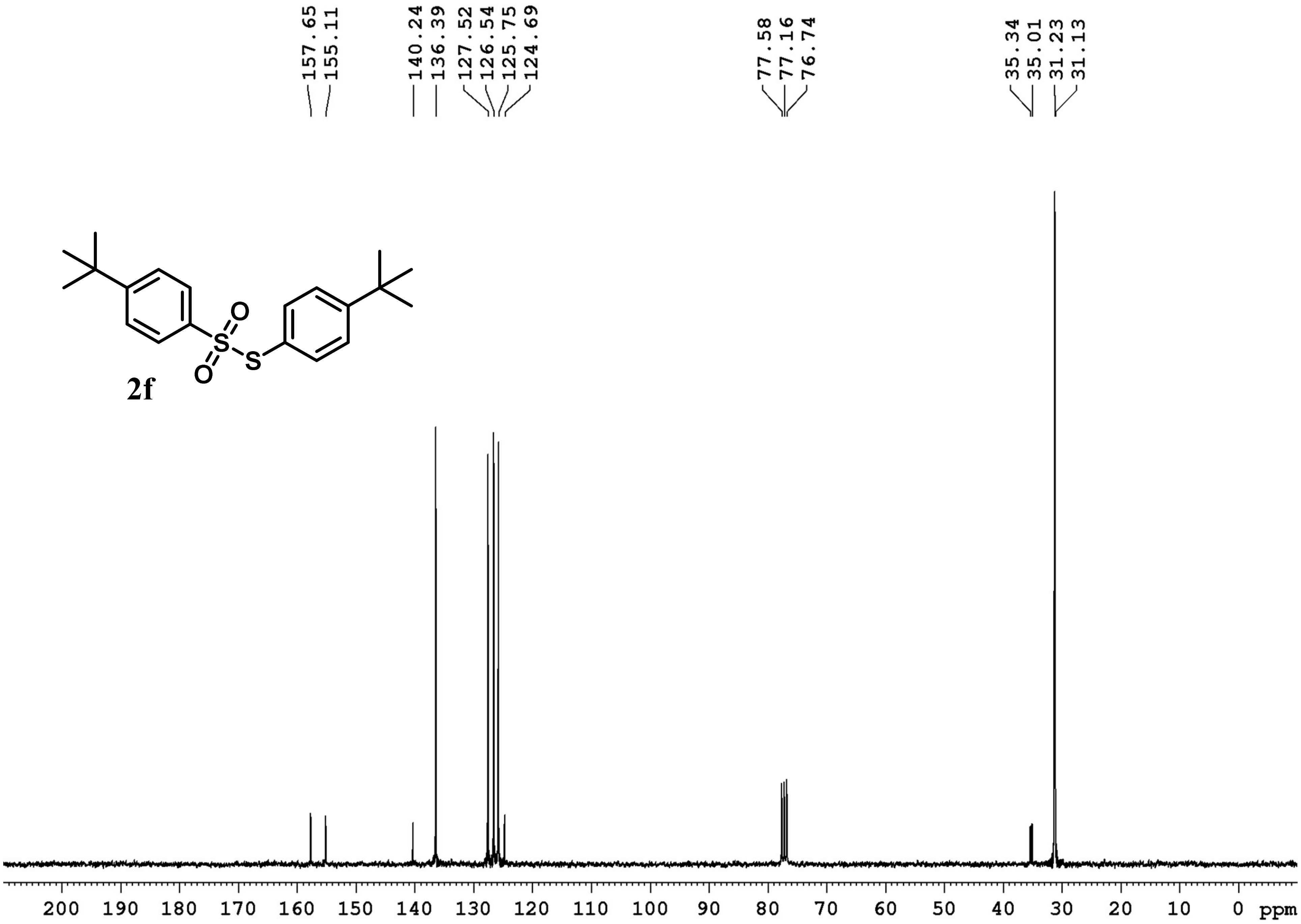

^{13}C {^{1}H} NMR of compound **2f** (75 MHz, $CDCl_3$)

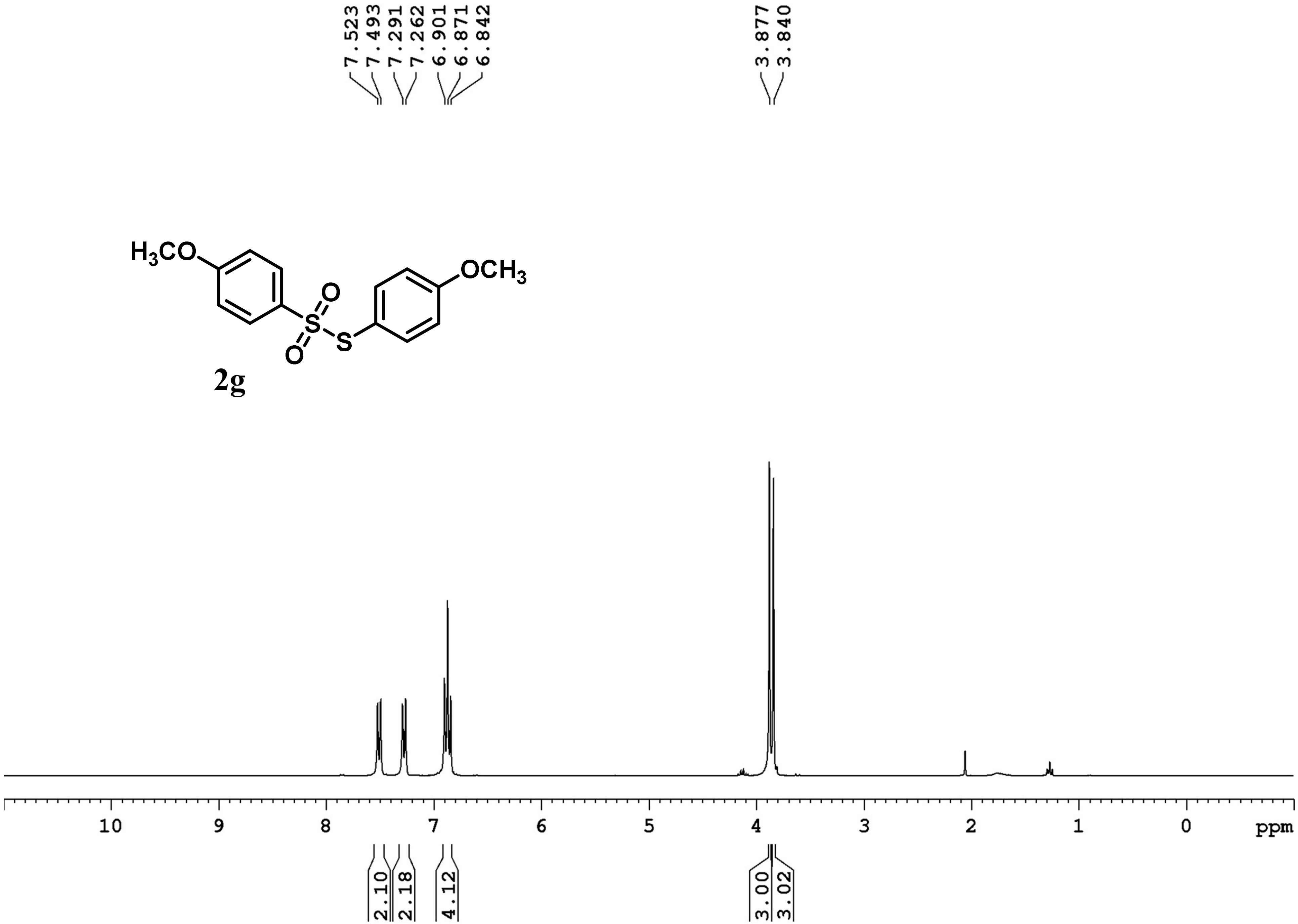

^{1}H NMR of compound **2g** (300 MHz, $CDCl_3$)

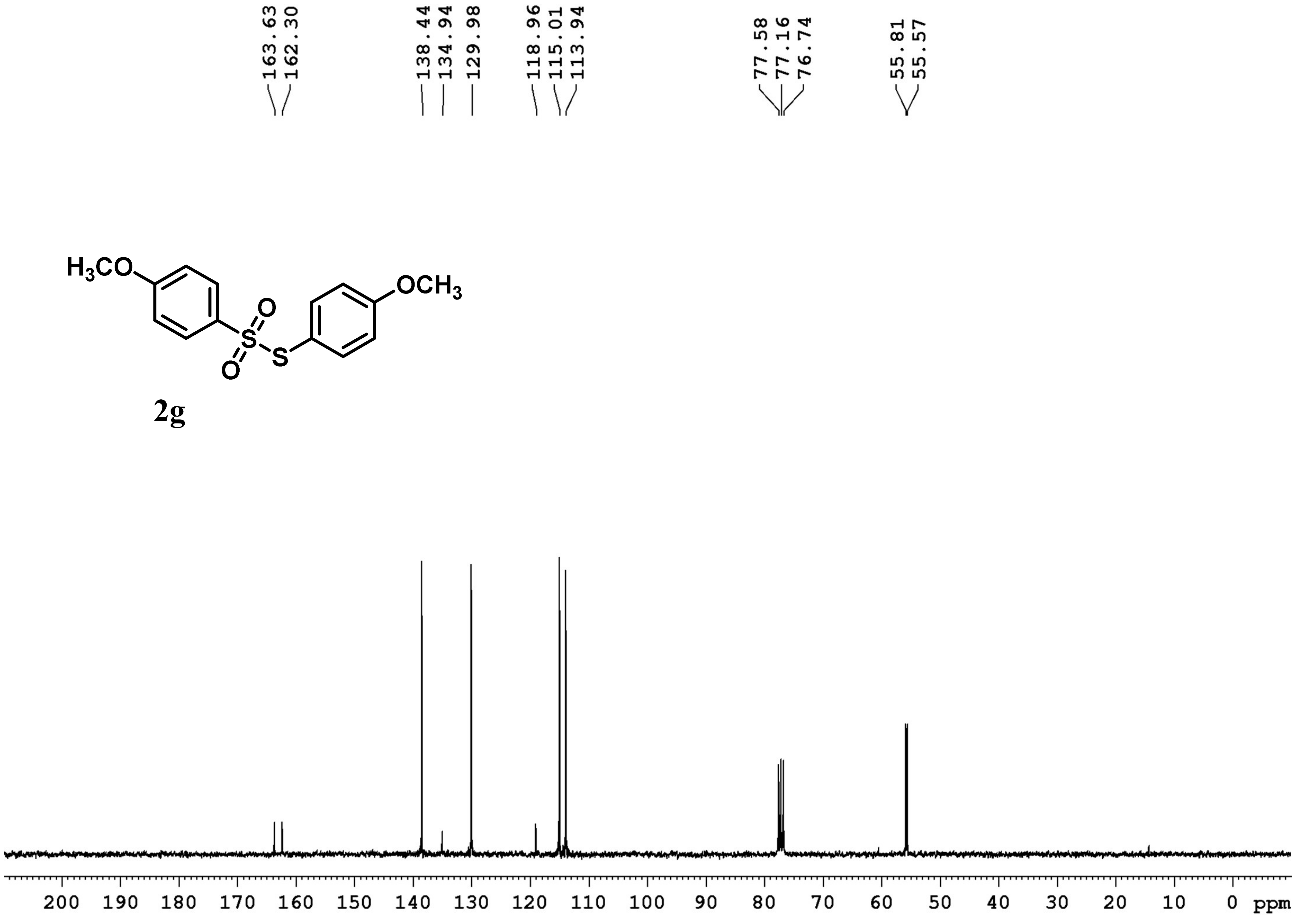

2g

^{13}C {^{1}H} NMR of compound **2g** (75 MHz, $CDCl_3$)

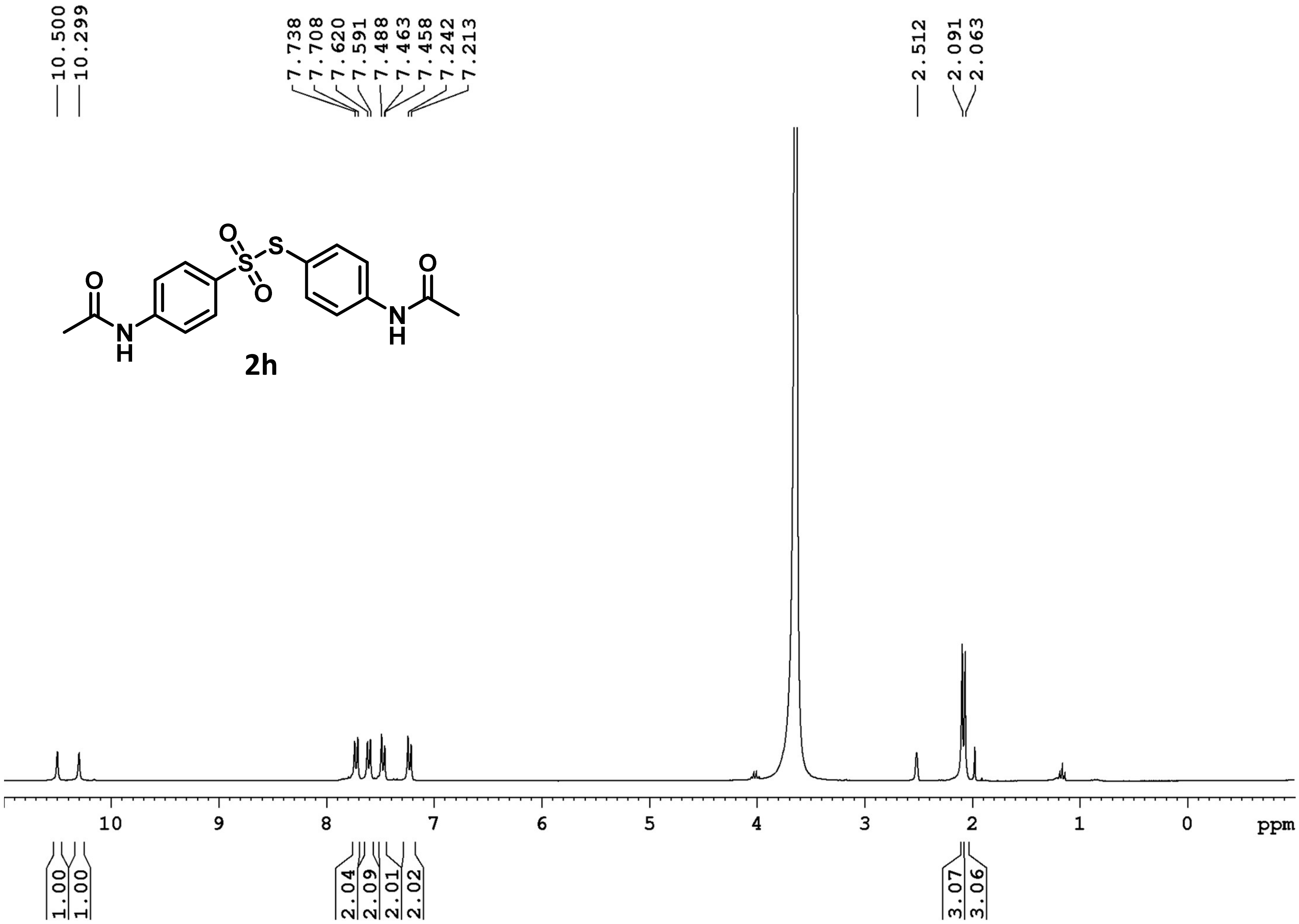

^{1}H NMR of compound **2h** (300 MHz, DMSO-d_6)

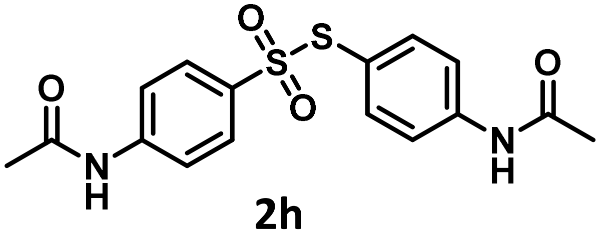

2h

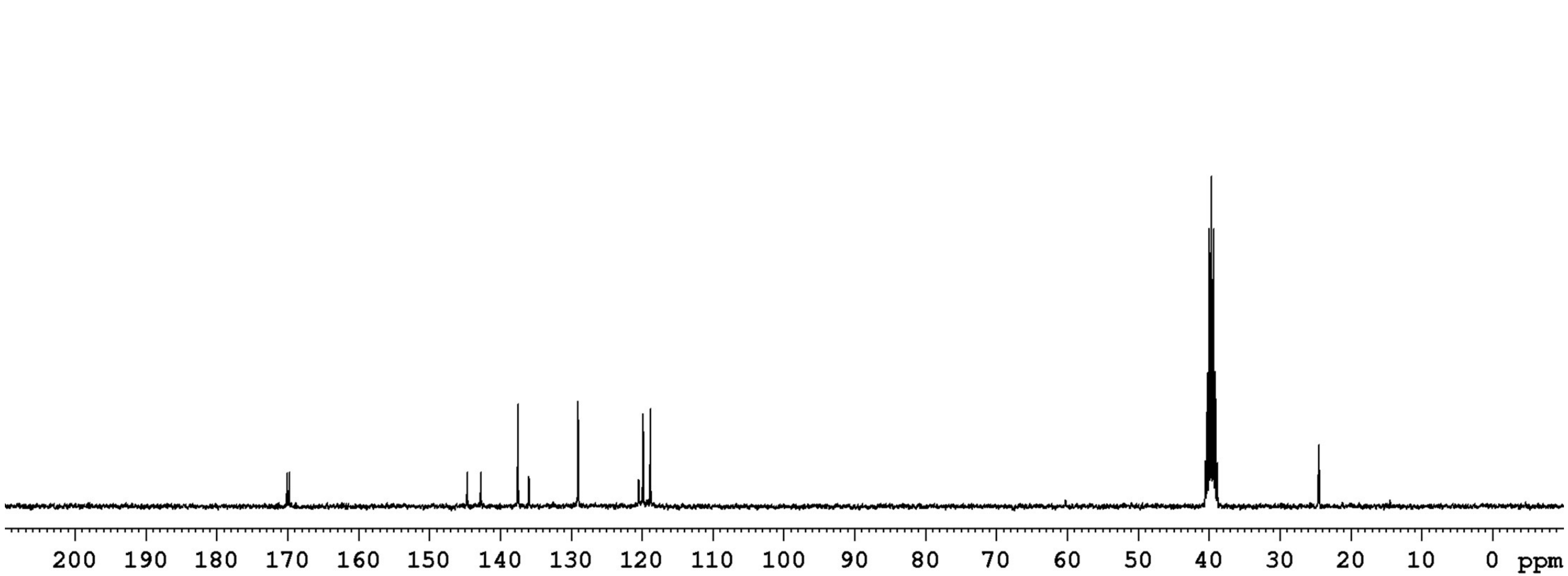

^{13}C {^{1}H} NMR of compound **2h** (75 MHz, DMSO-d_6)

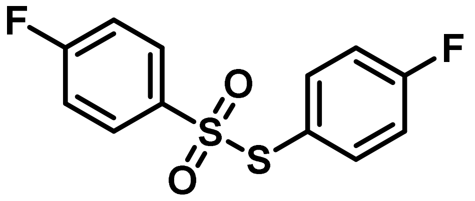

2i

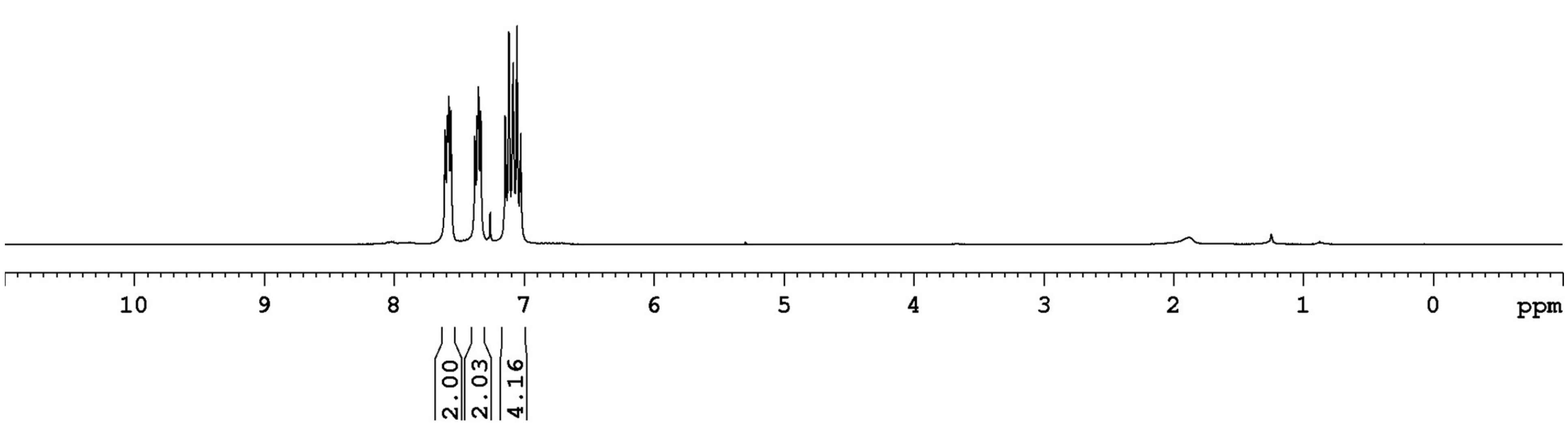

^{1}H NMR of compound **2i** (300 MHz, $CDCl_3$)

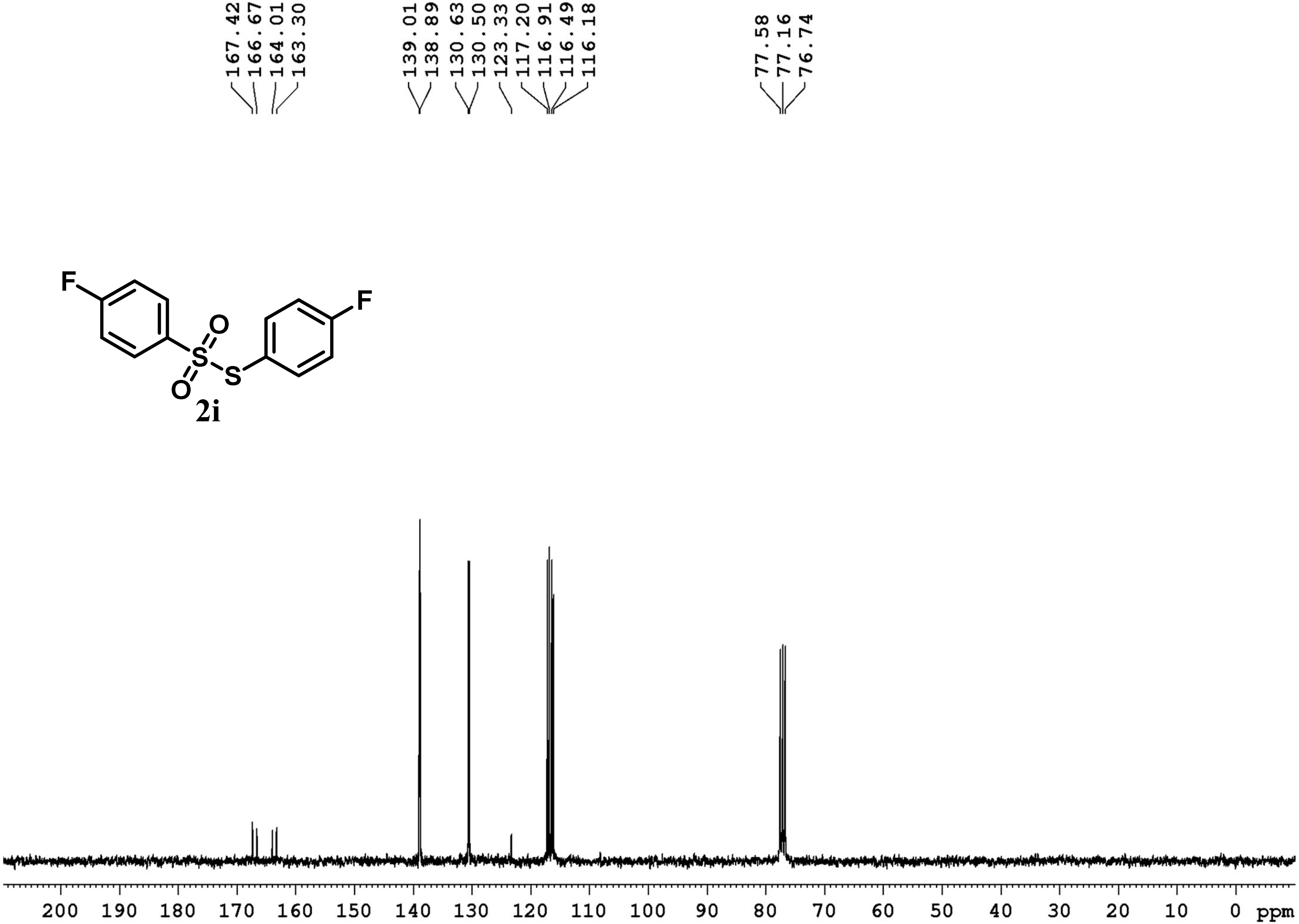

2i

^{13}C {^{1}H} NMR of compound **2i** (75 MHz, $CDCl_3$)

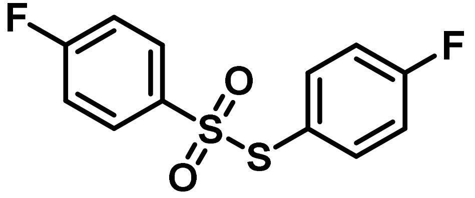

2i

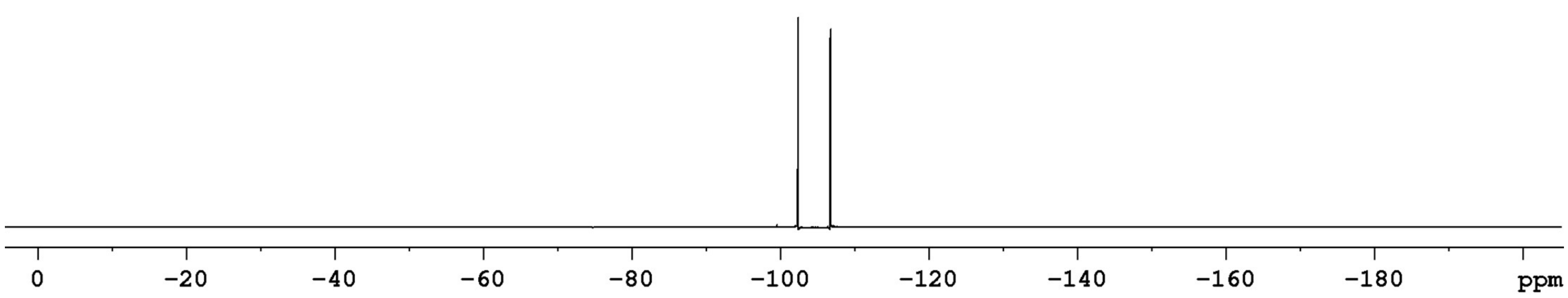

^{19}F {^{1}H} NMR of compound **2i** (283 MHz, $CDCl_3$)

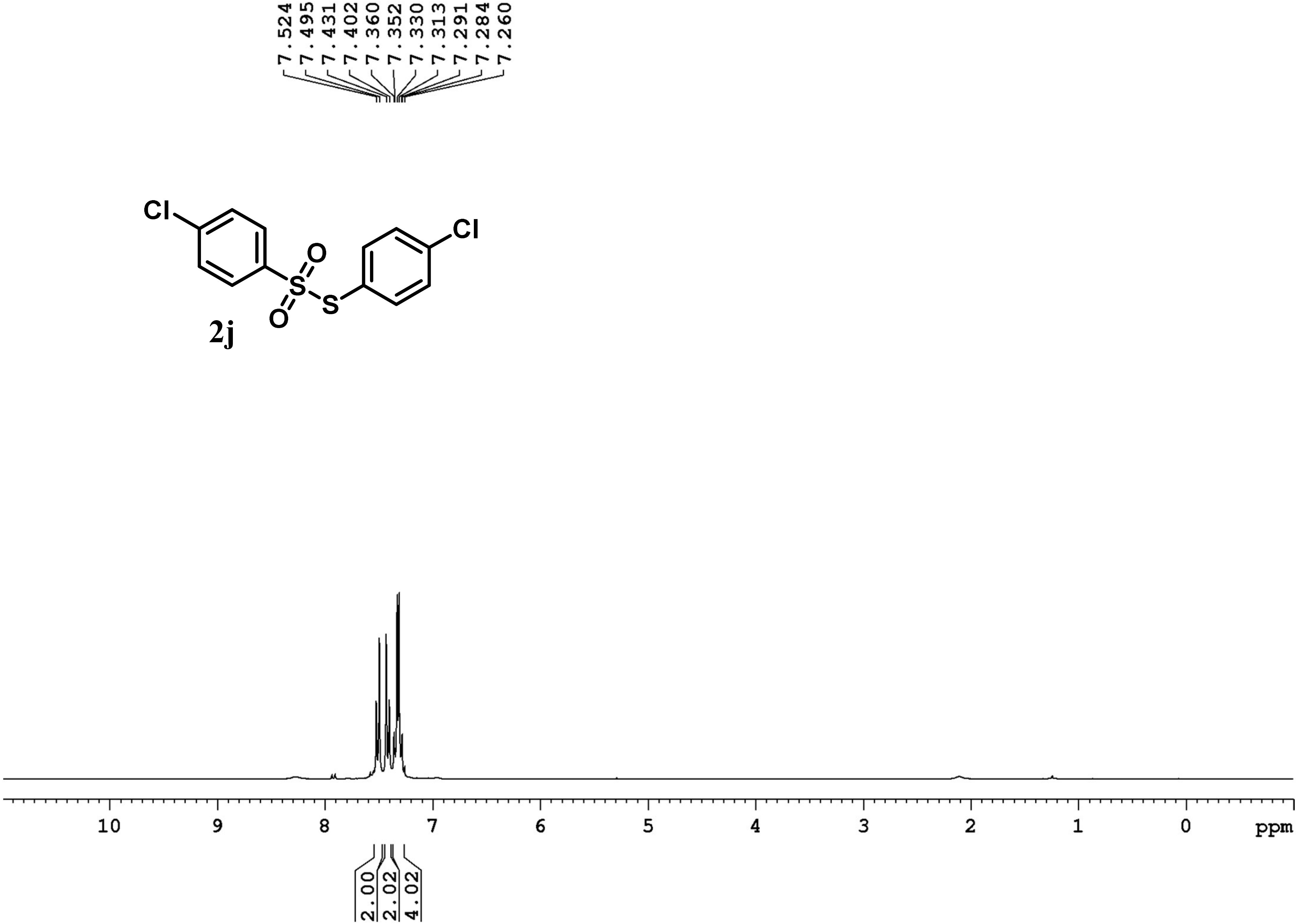

^{1}H NMR of compound **2j** (300 MHz, $CDCl_3$)

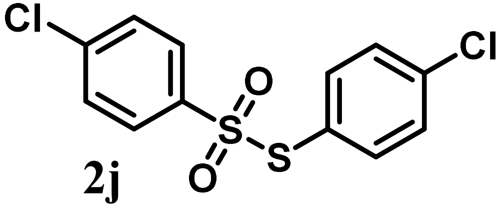

2j

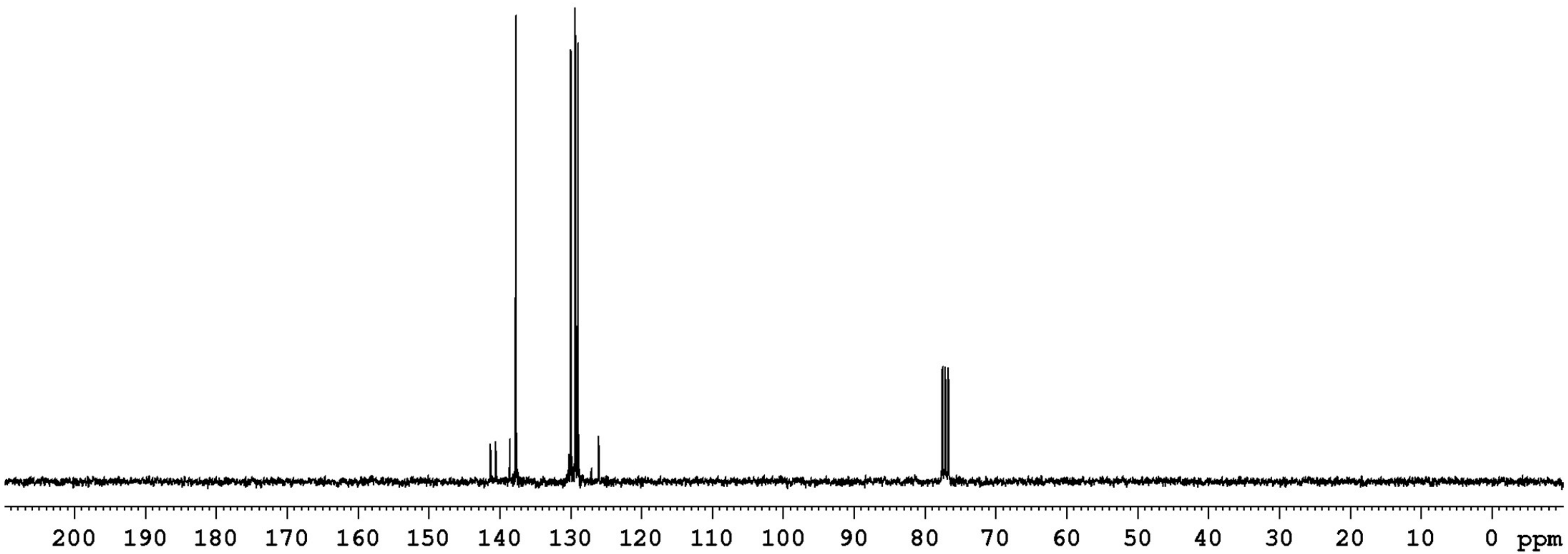

$^{13}C\ \{^{1}H\}$ NMR of compound **2j** (75 MHz, $CDCl_3$)

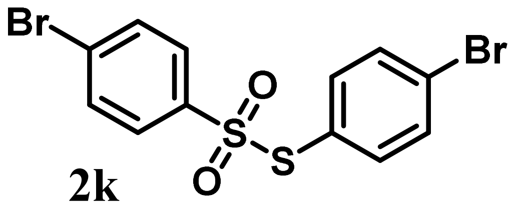

2k

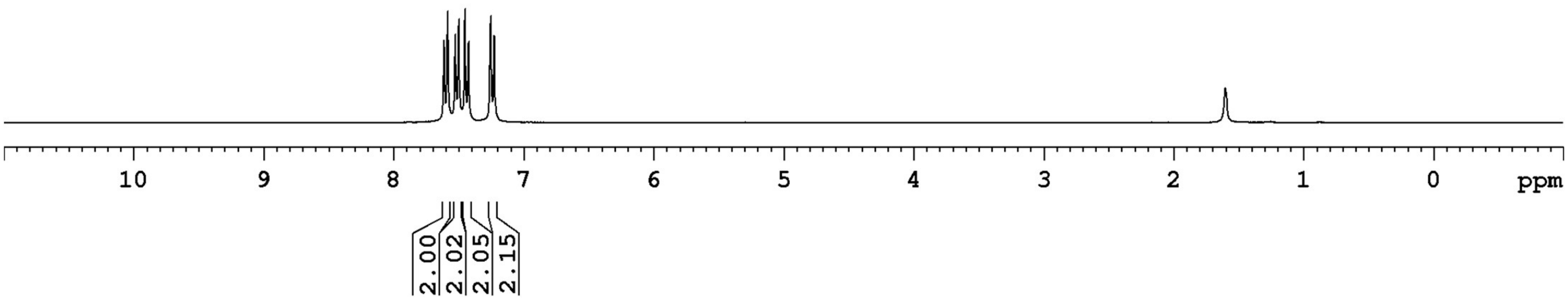

^{1}H NMR of compound **2k** (300 MHz, $CDCl_3$)

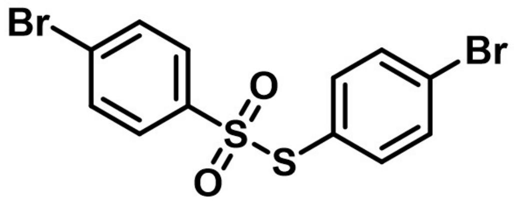

2k

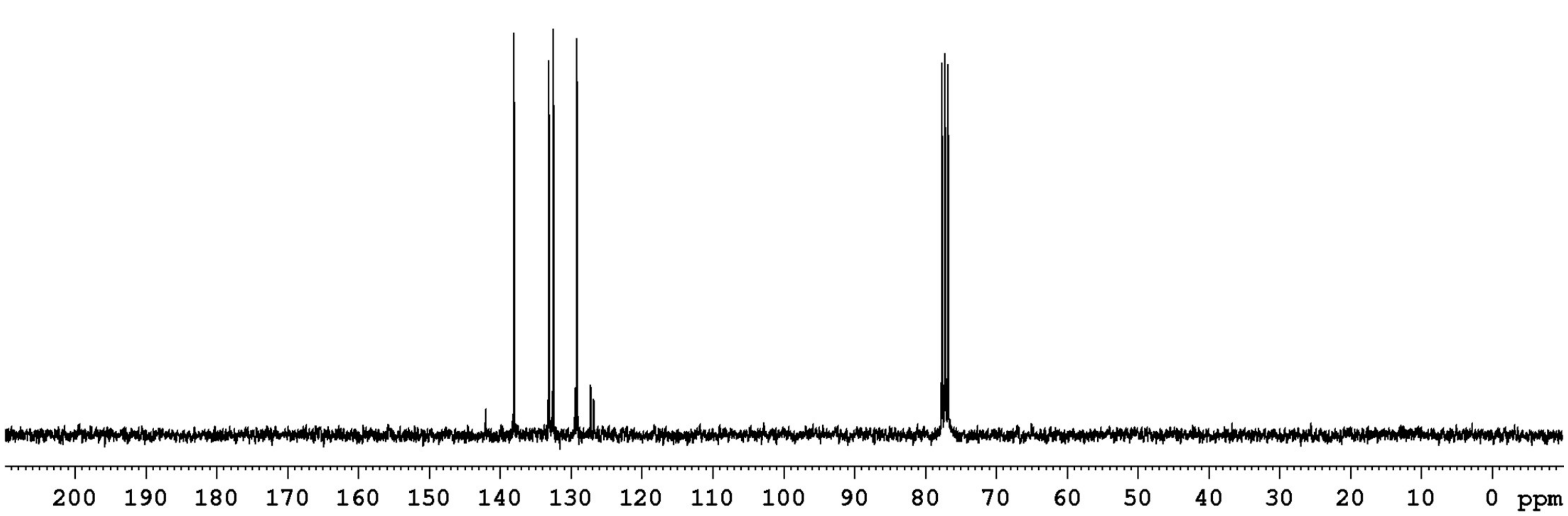

^{13}C {^{1}H} NMR of compound **2k** (75 MHz, $CDCl_3$)

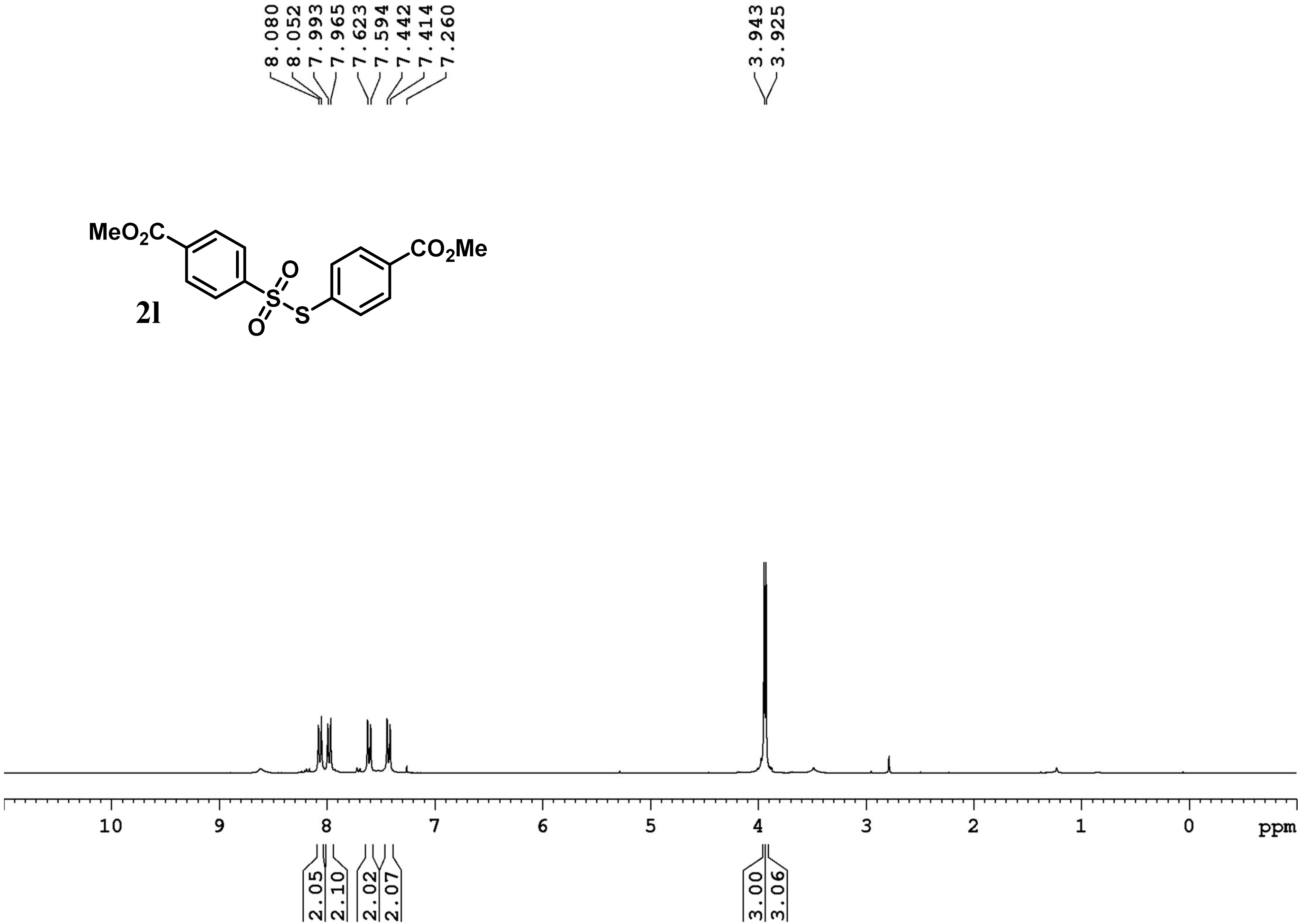

^{1}H NMR of compound **2l** (300 MHz, $CDCl_3$)

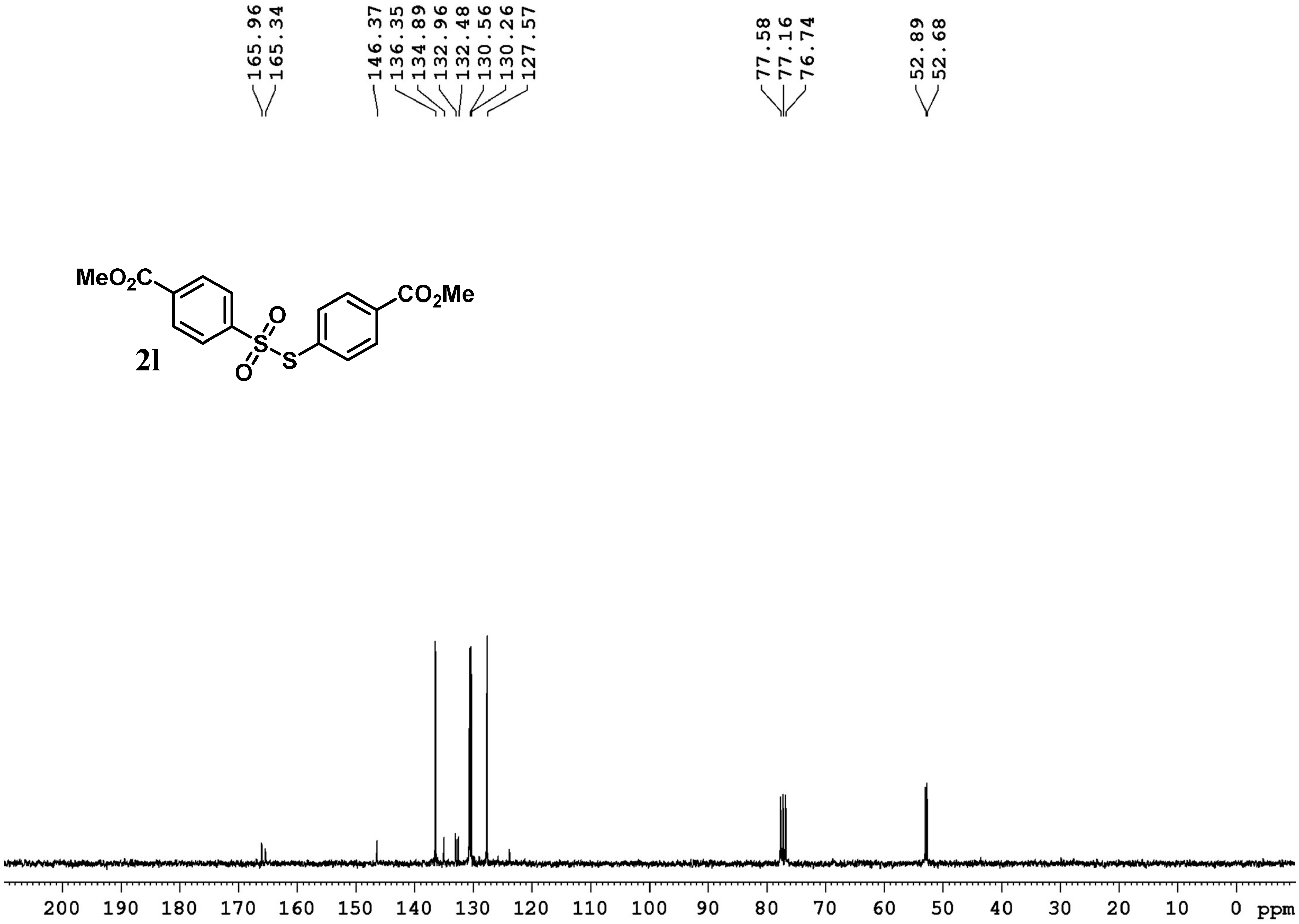

^{13}C {^{1}H} NMR of compound **2l** (75 MHz, $CDCl_3$)

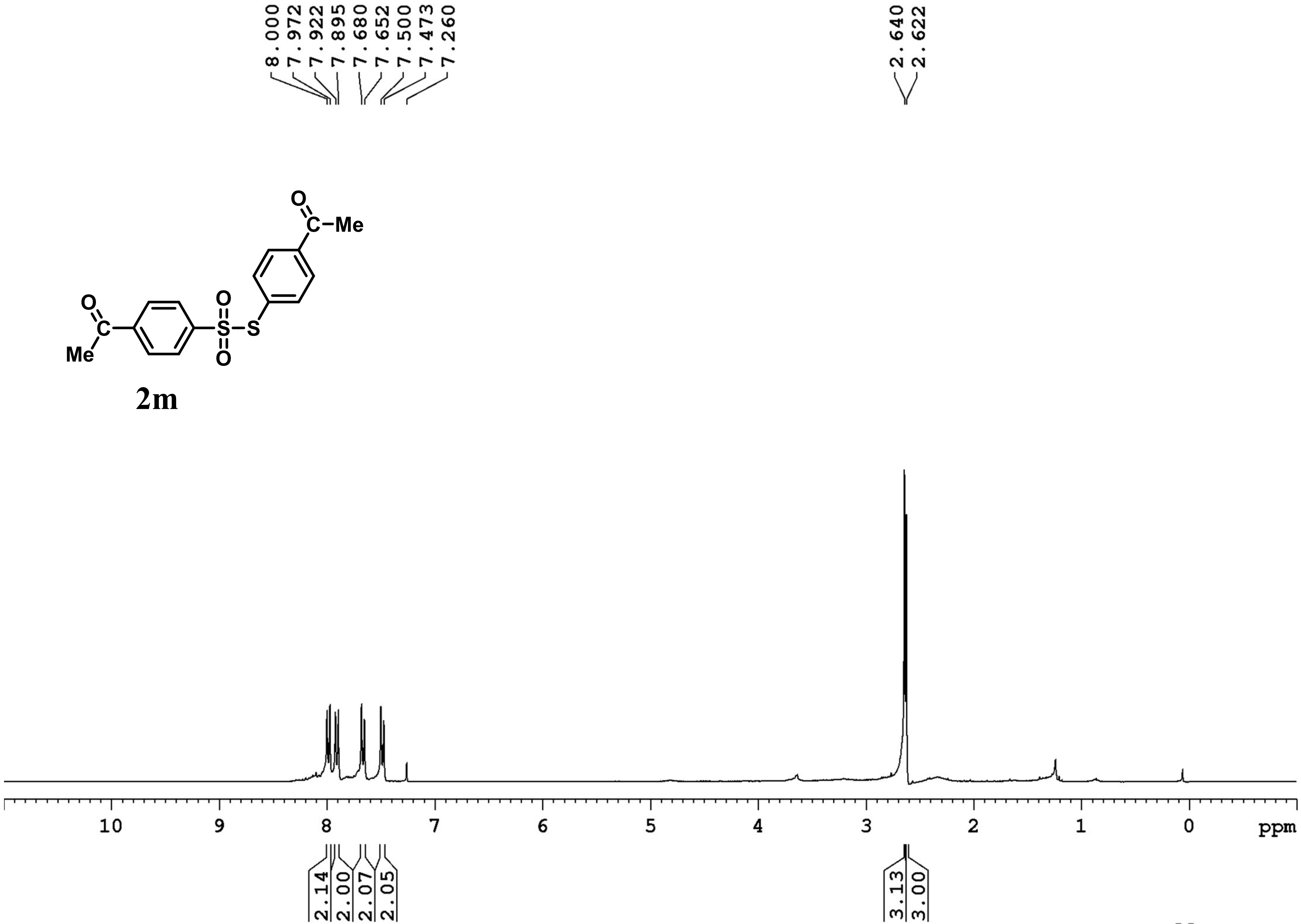

^{1}H NMR of compound **2m** (300 MHz, $CDCl_3$)

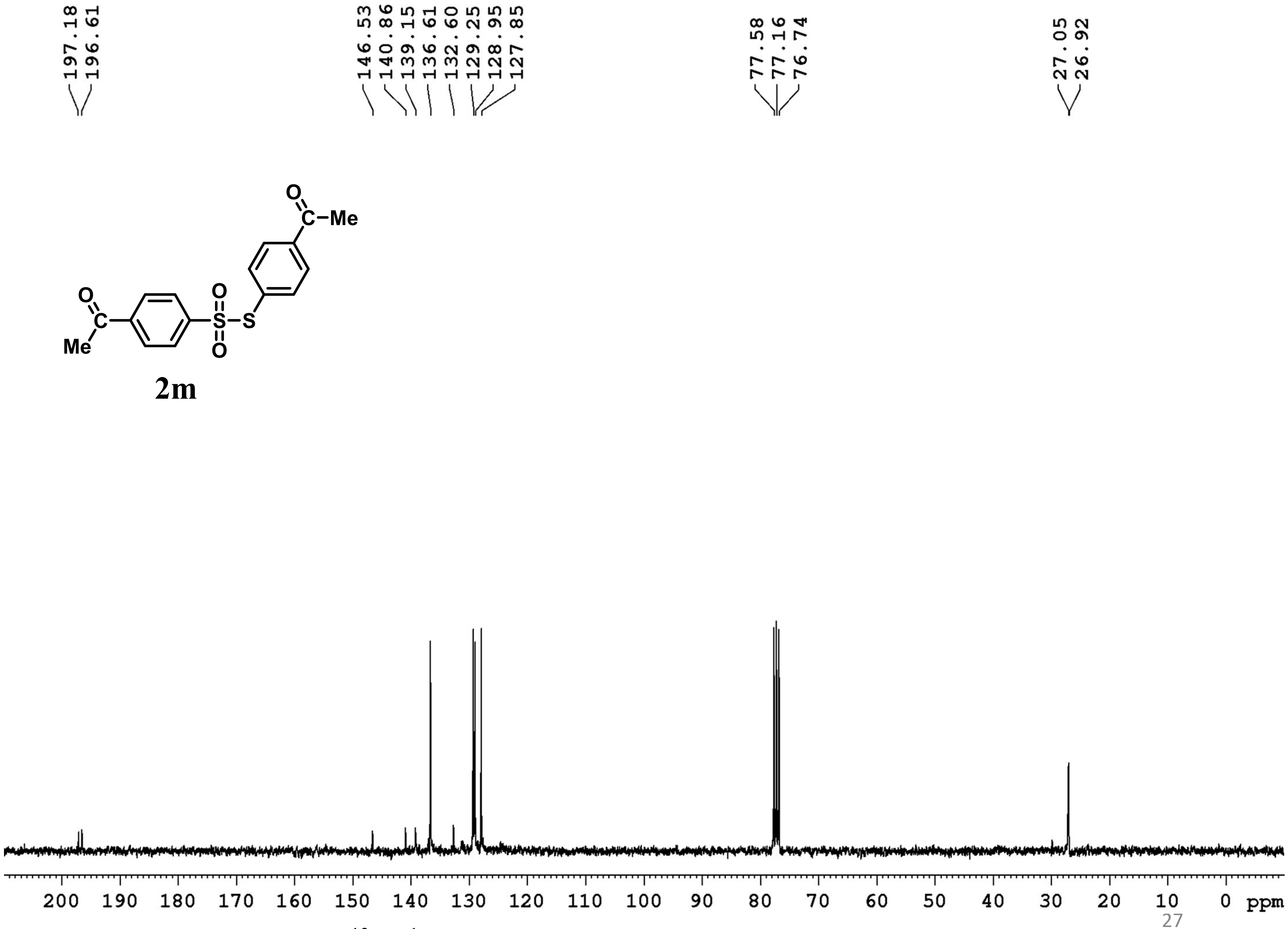

27

^{13}C {^{1}H} NMR of compound **2m** (75 MHz, $CDCl_3$)

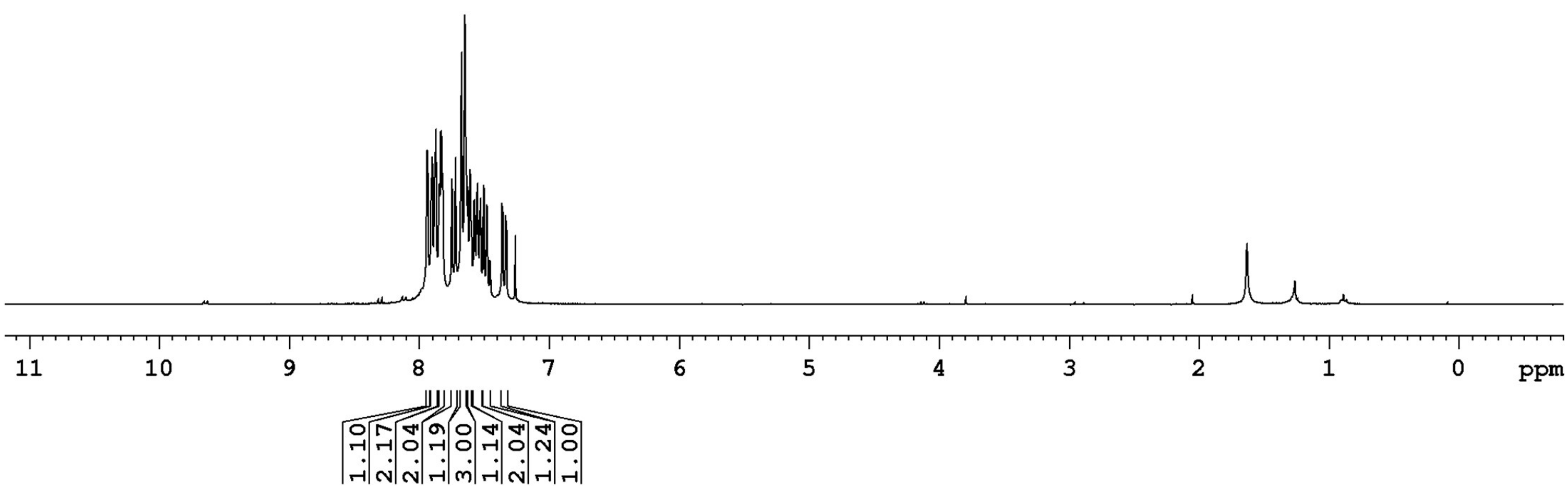

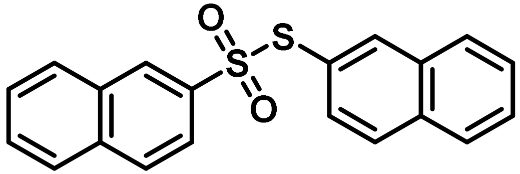

2n

^{1}H NMR of compound **2n** (300 MHz, $CDCl_3$)

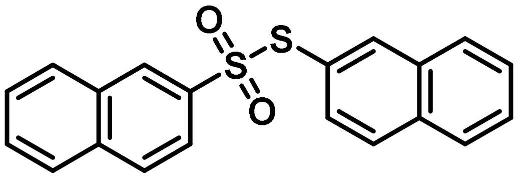

2n

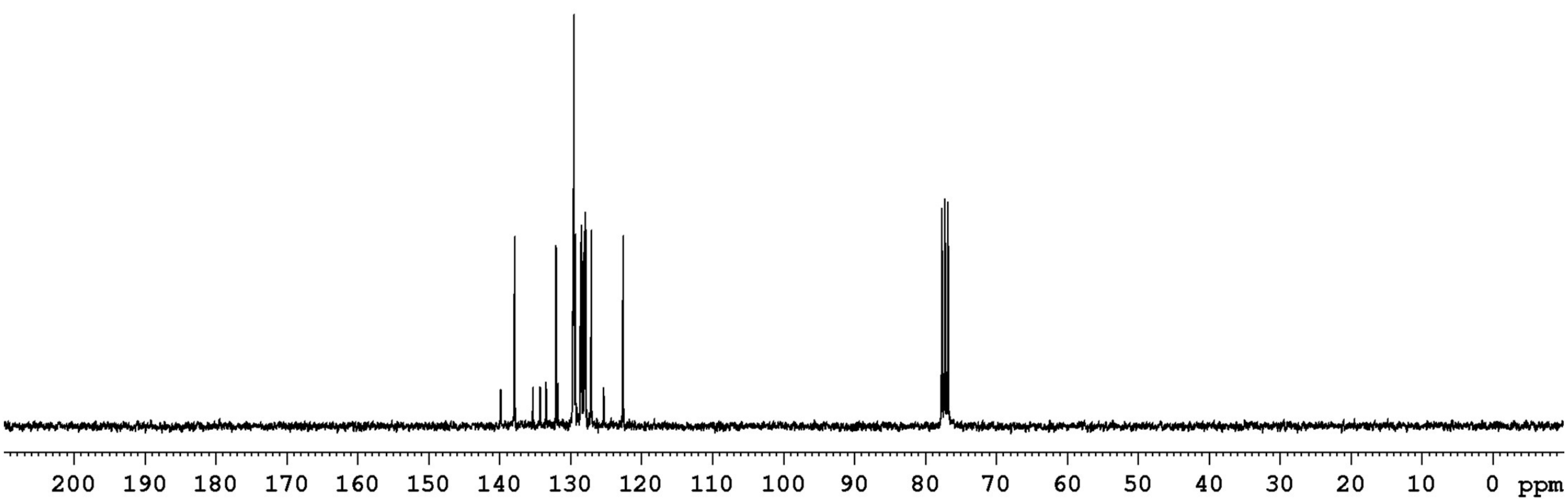

$^{13}C\ \{^{1}H\}$ NMR of compound **2n** (75 MHz, $CDCl_3$)

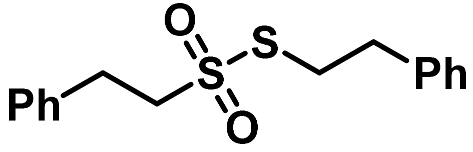

2o

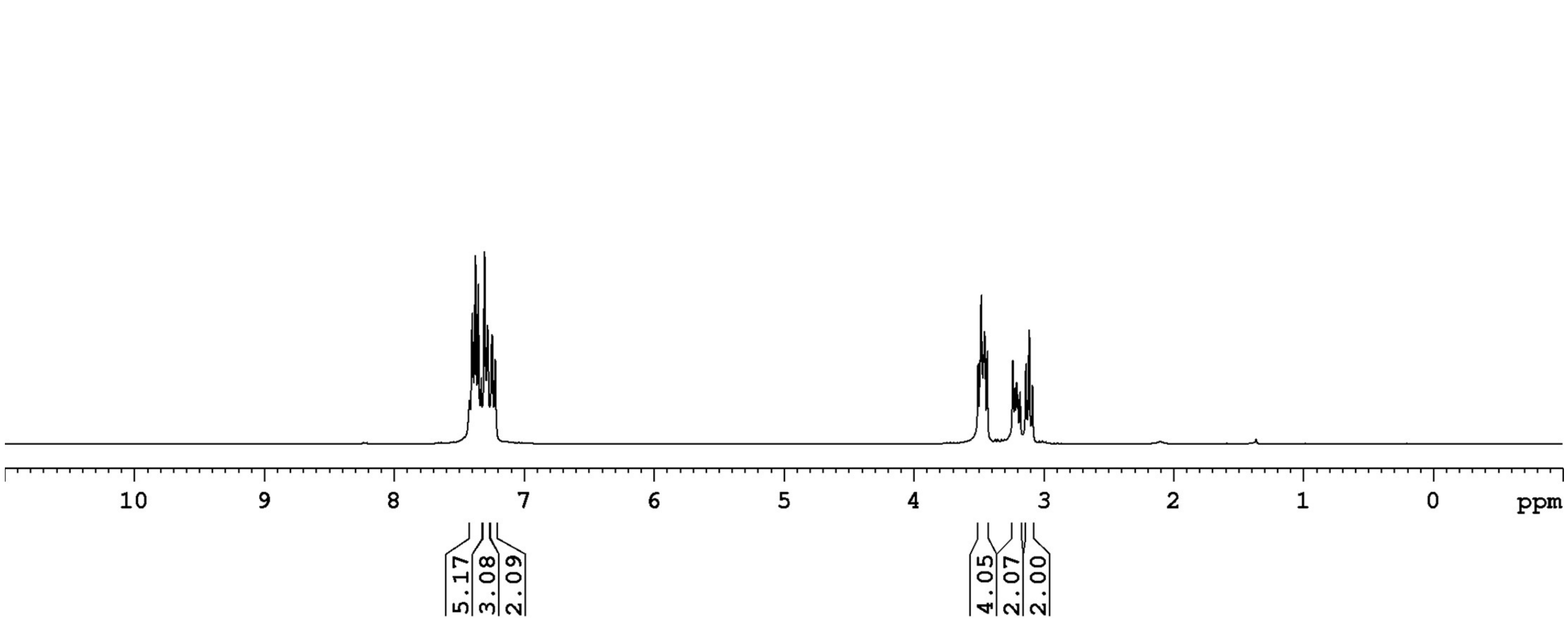

^{1}H NMR of compound **2o** (300 MHz, $CDCl_3$)

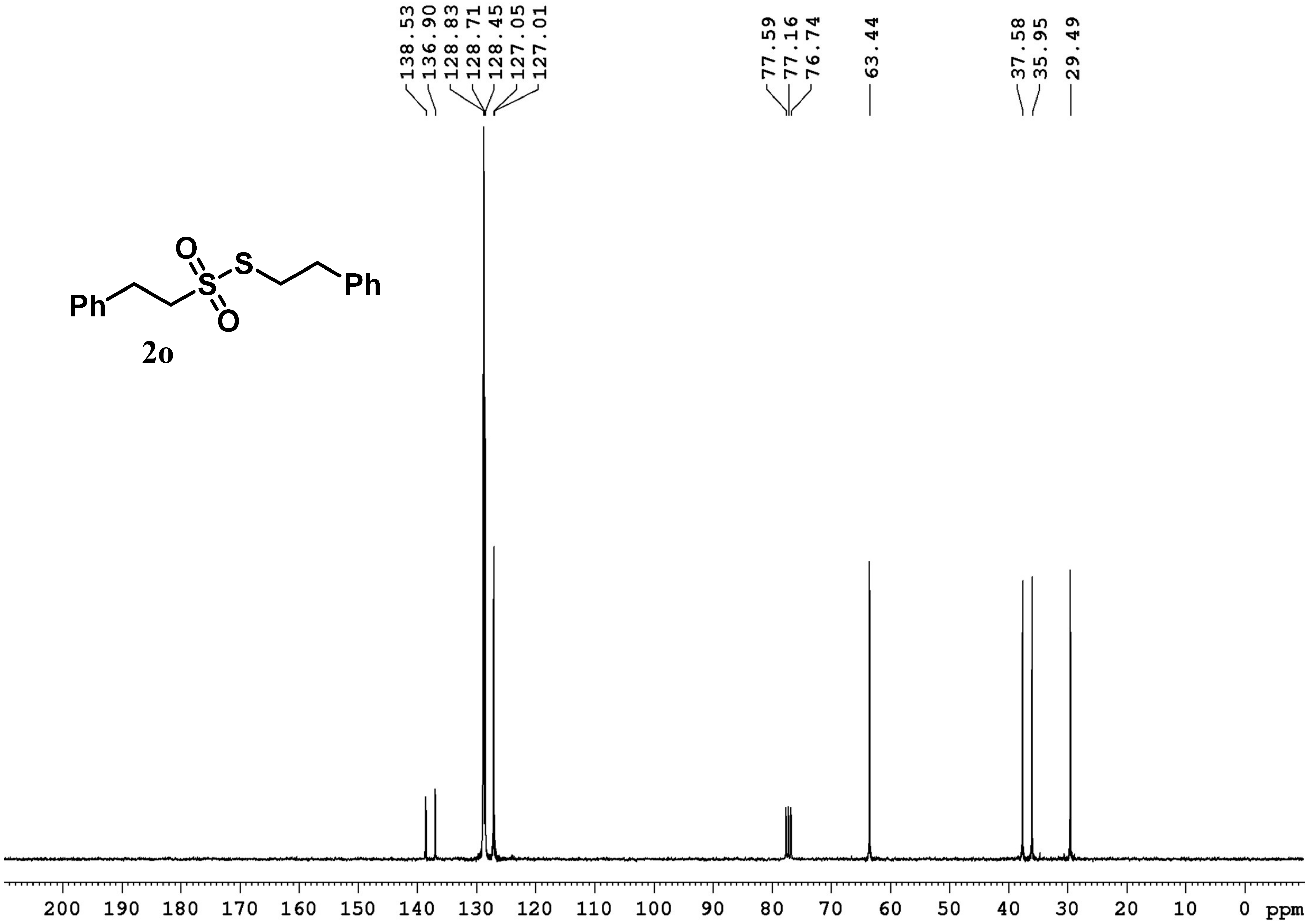

^{13}C {^{1}H} NMR of compound **2o** (75 MHz, $CDCl_3$)

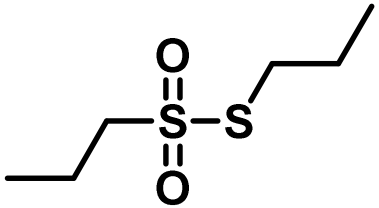

2p

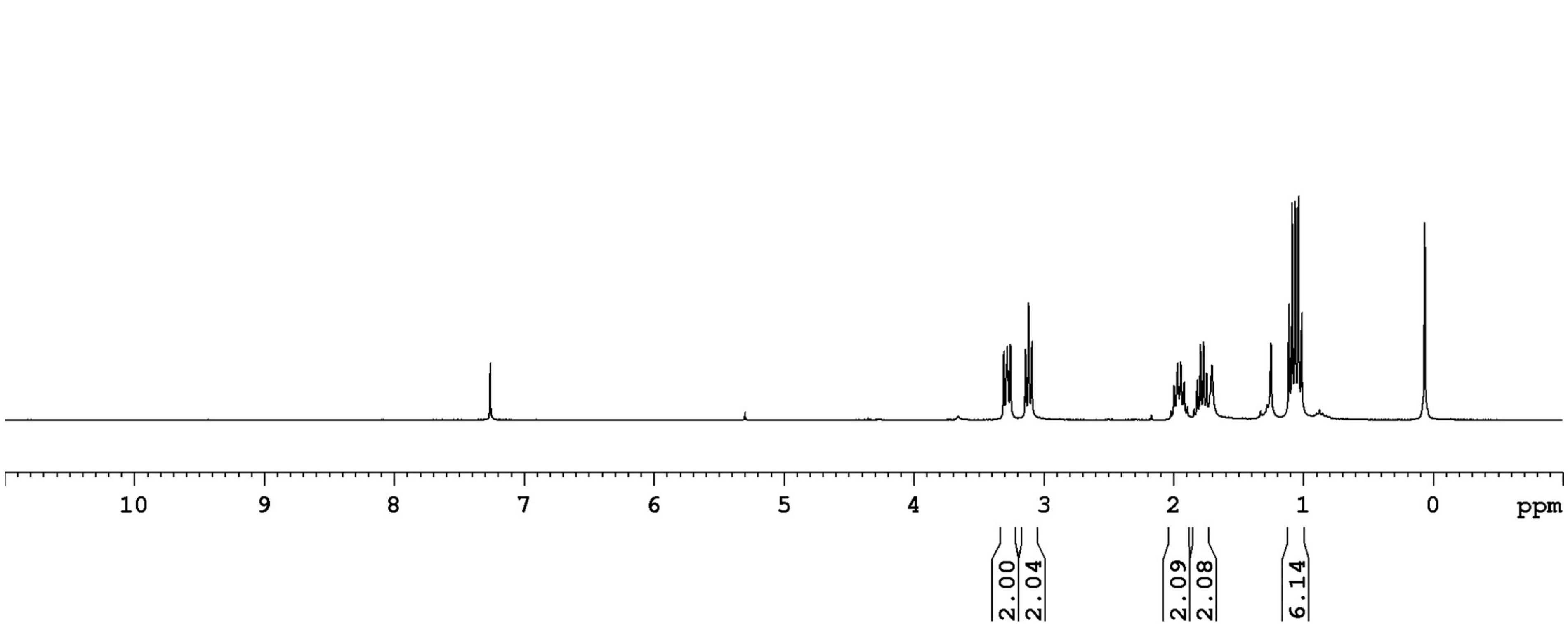

^{1}H NMR of compound **2p** (300 MHz, $CDCl_3$)

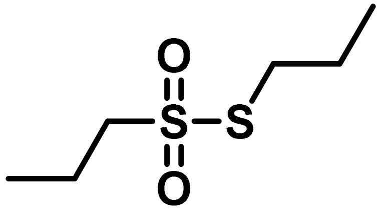

2p

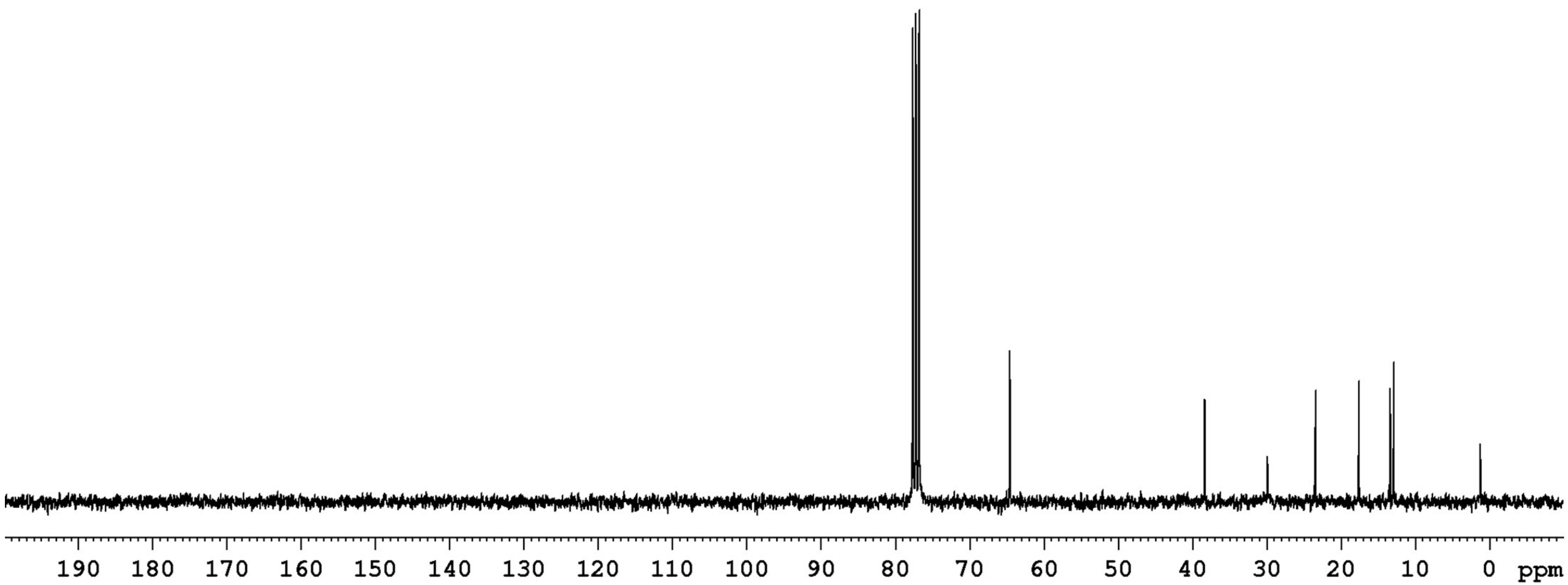

^{13}C {^{1}H} NMR of compound **2p** (75 MHz, $CDCl_3$)

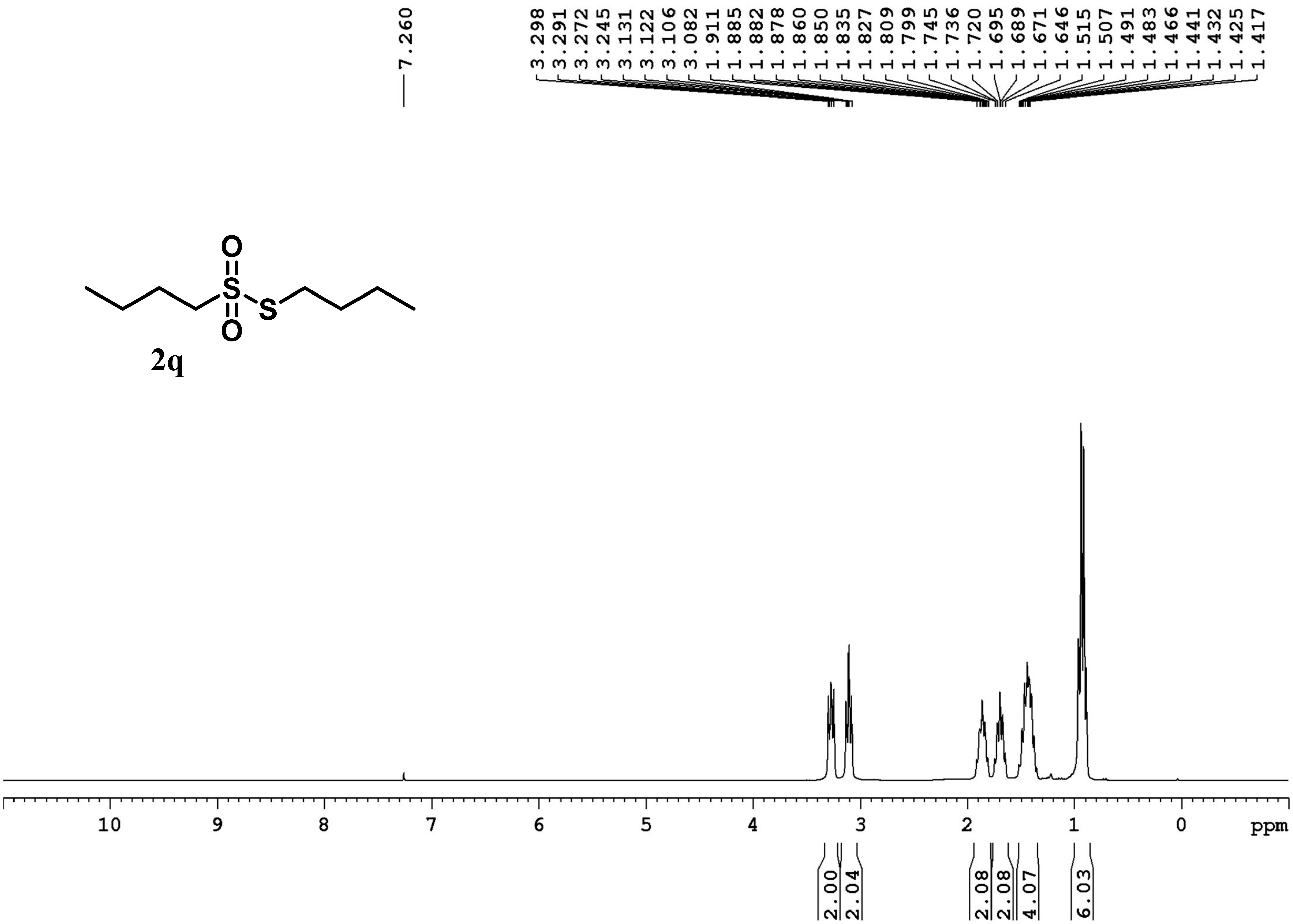

^{1}H NMR of compound **2q** (300 MHz, $CDCl_3$)

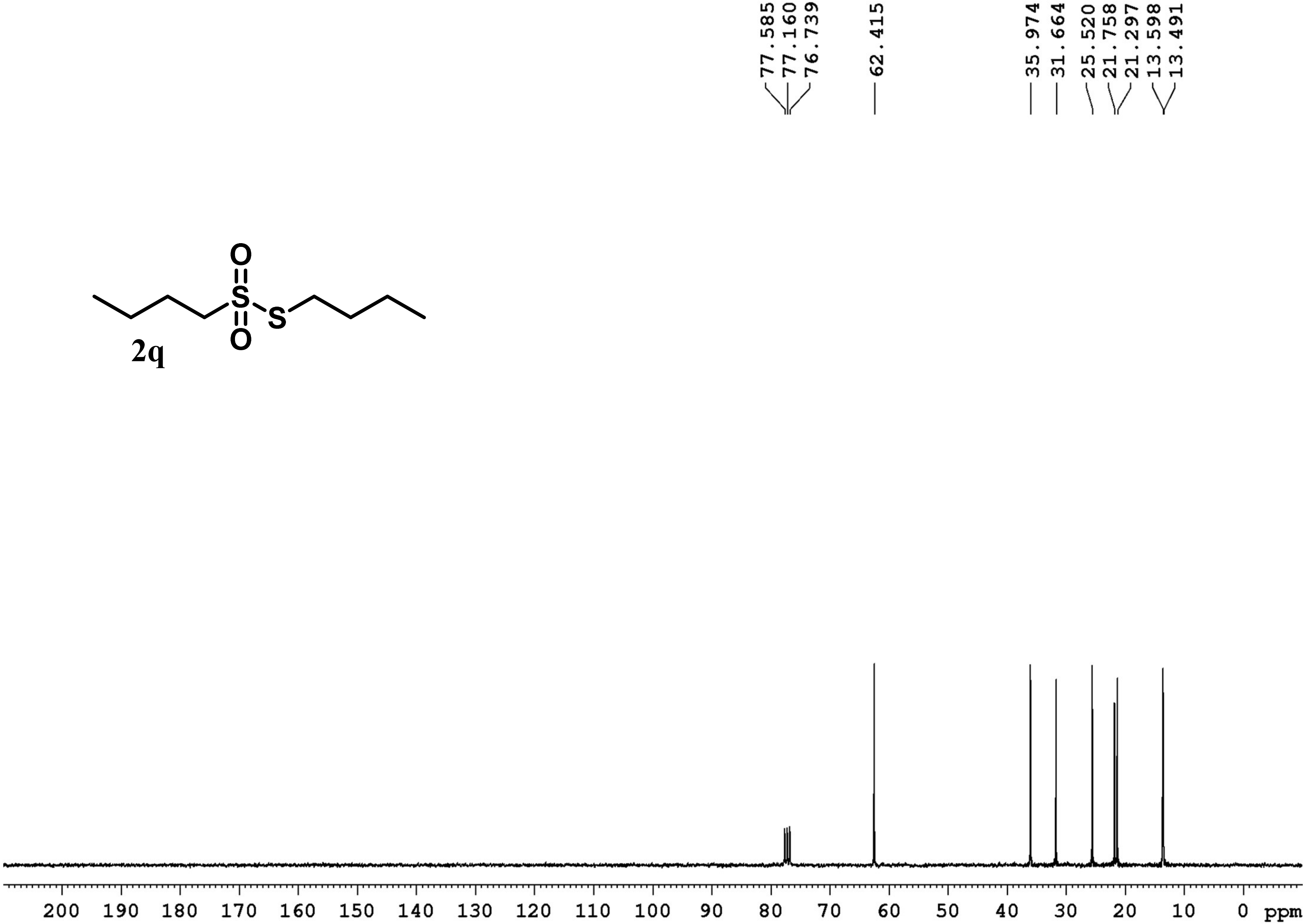

^{13}C {^{1}H} NMR of compound **2q** (75 MHz, $CDCl_3$)

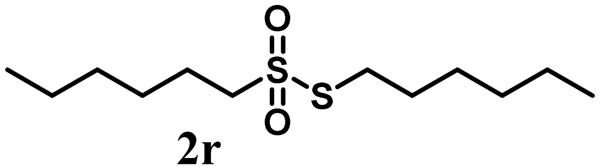

2r

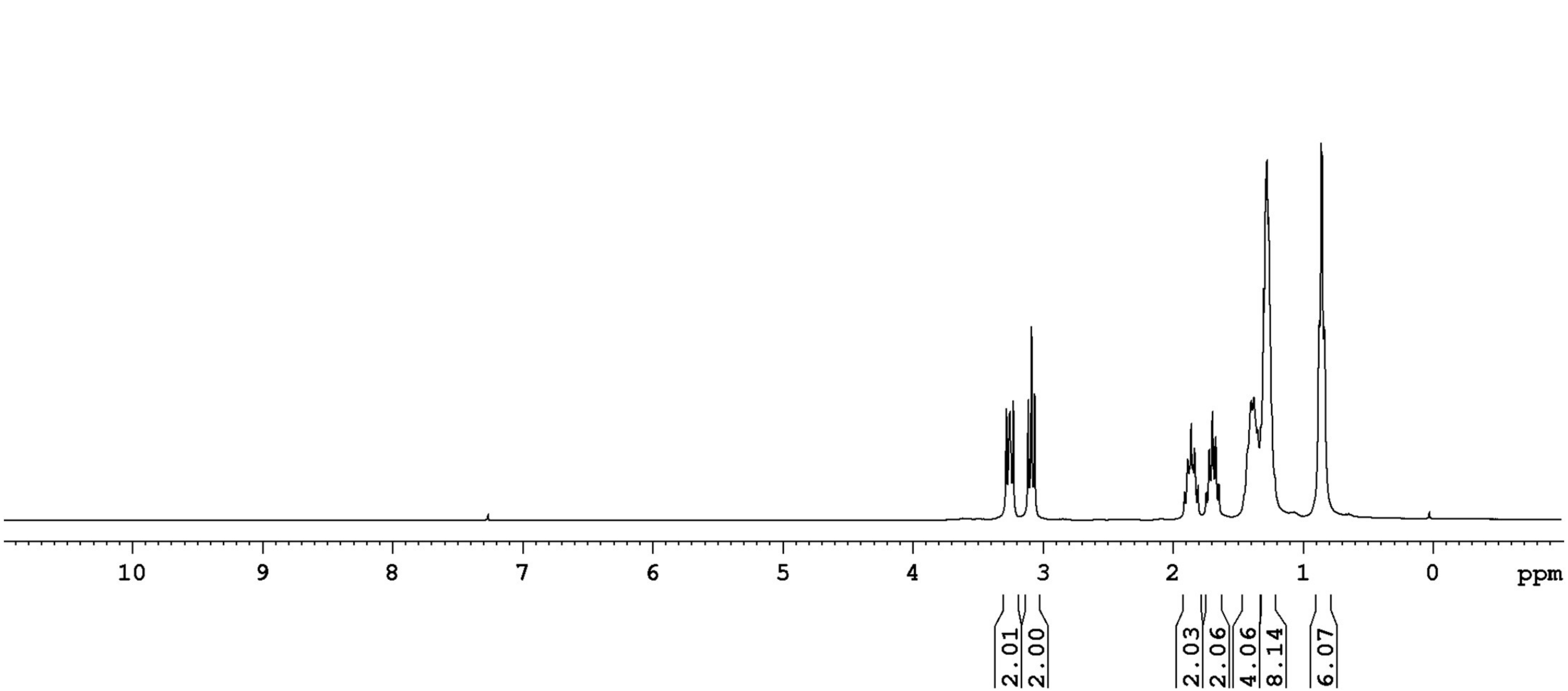

^{1}H NMR of compound **2r** (300 MHz, $CDCl_3$)

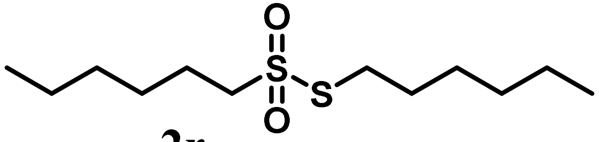

2r

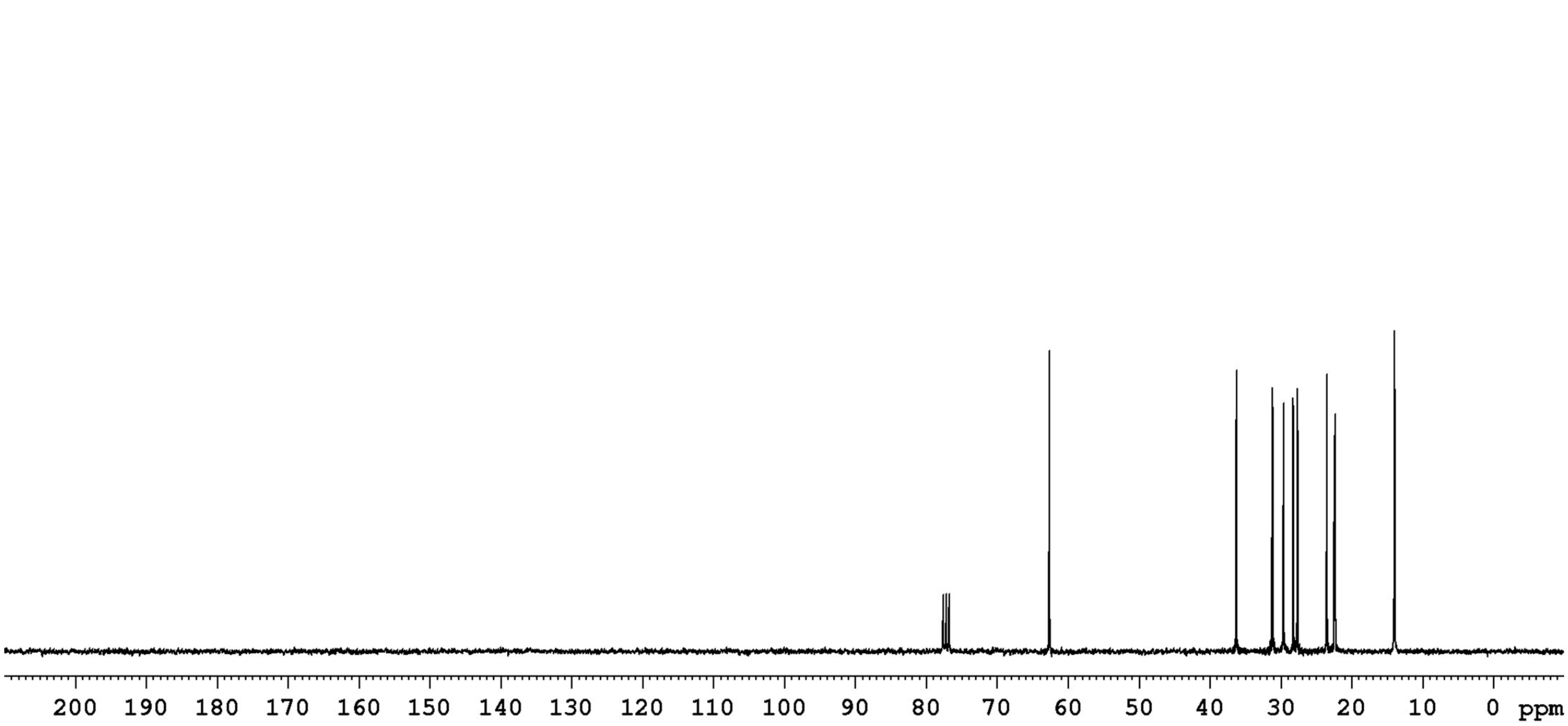

^{13}C {^{1}H} NMR of compound **2r** (75 MHz, $CDCl_3$)

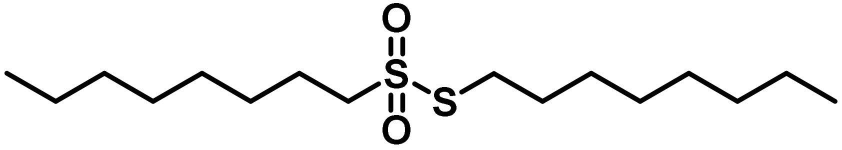

2s

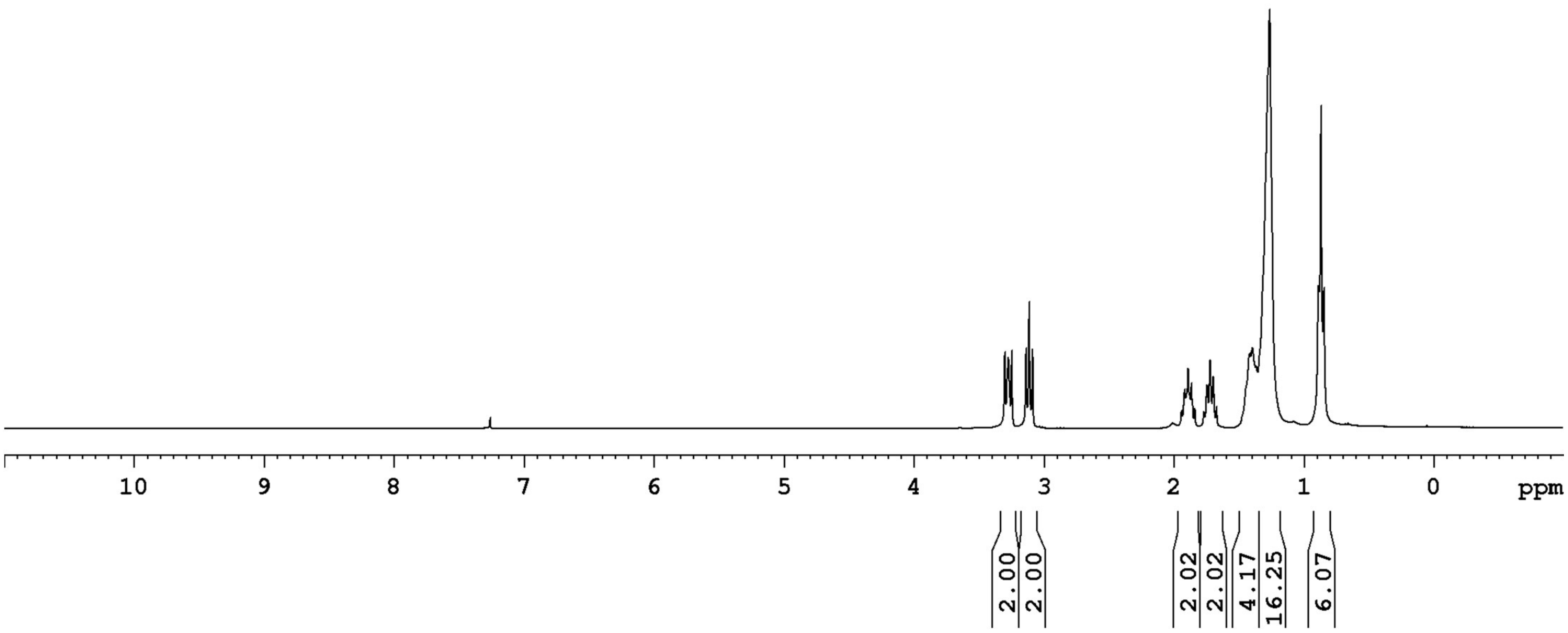

^{1}H NMR of compound **2s** (300 MHz, $CDCl_3$)

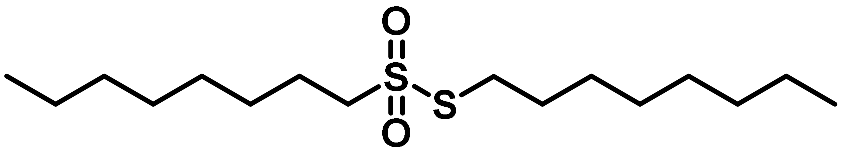

2s

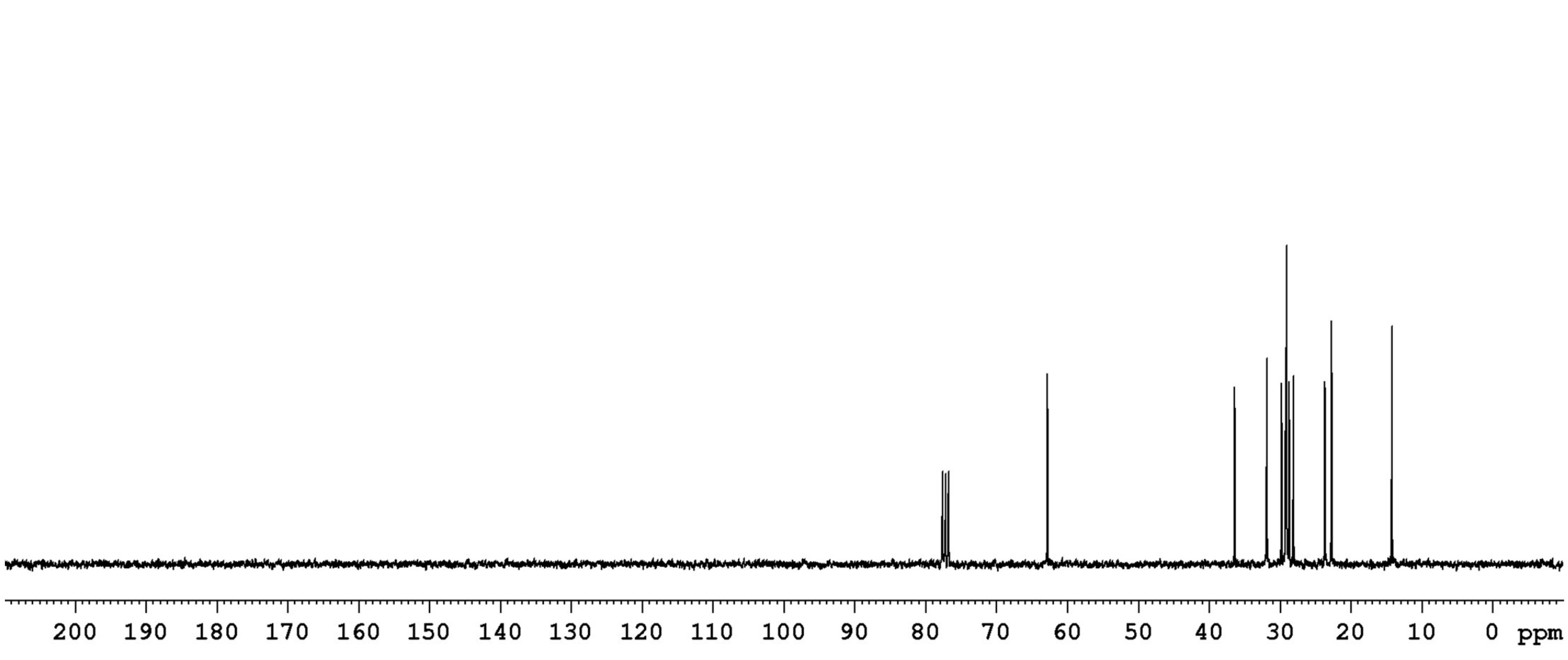

^{13}C {^{1}H} NMR of compound **2s** (75 MHz, $CDCl_3$)

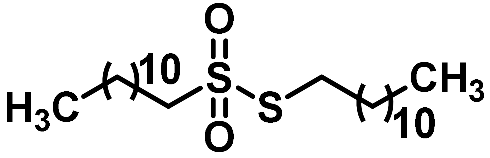

2t

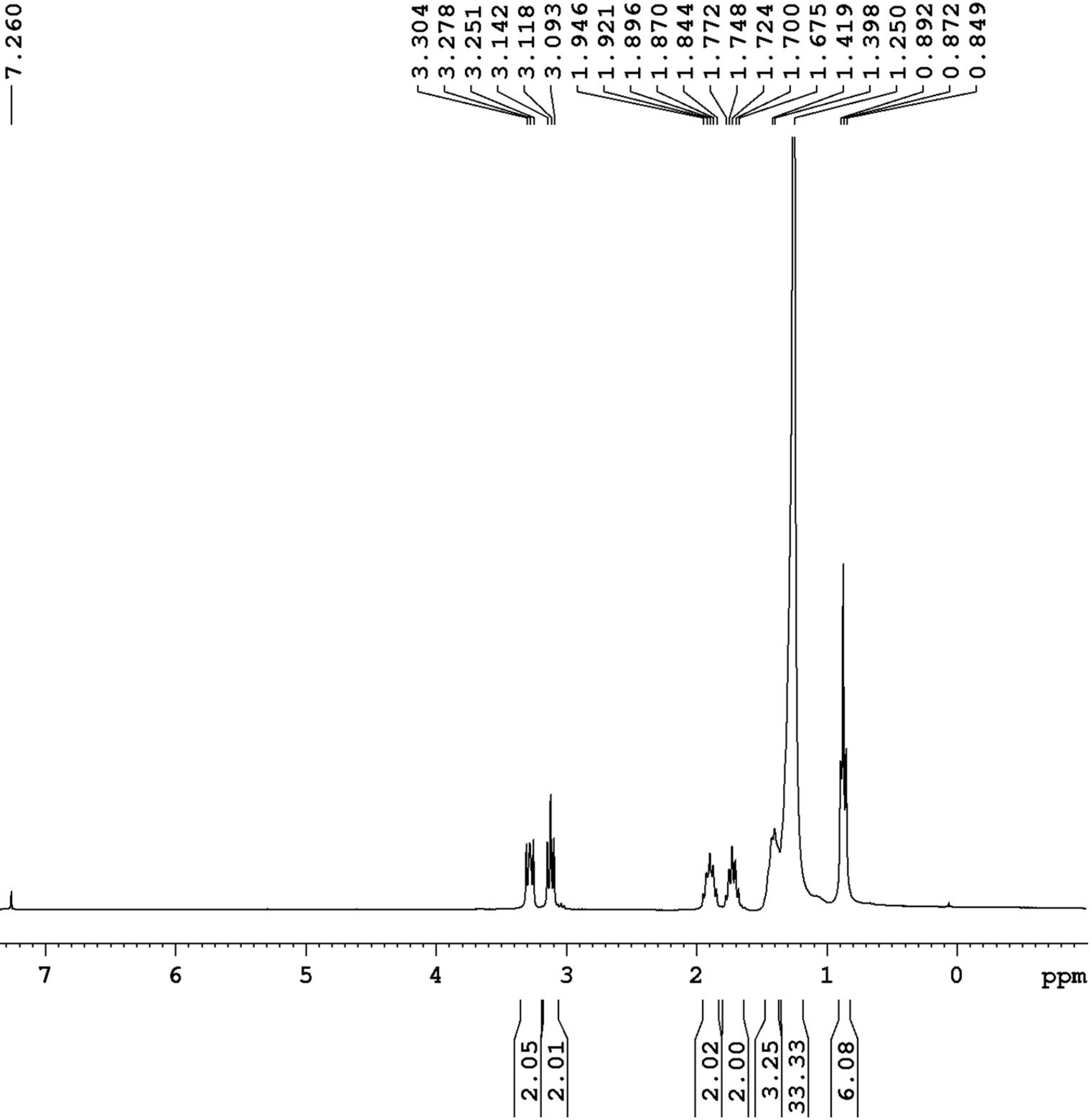

^{1}H NMR of compound **2t** (300 MHz, $CDCl_3$)

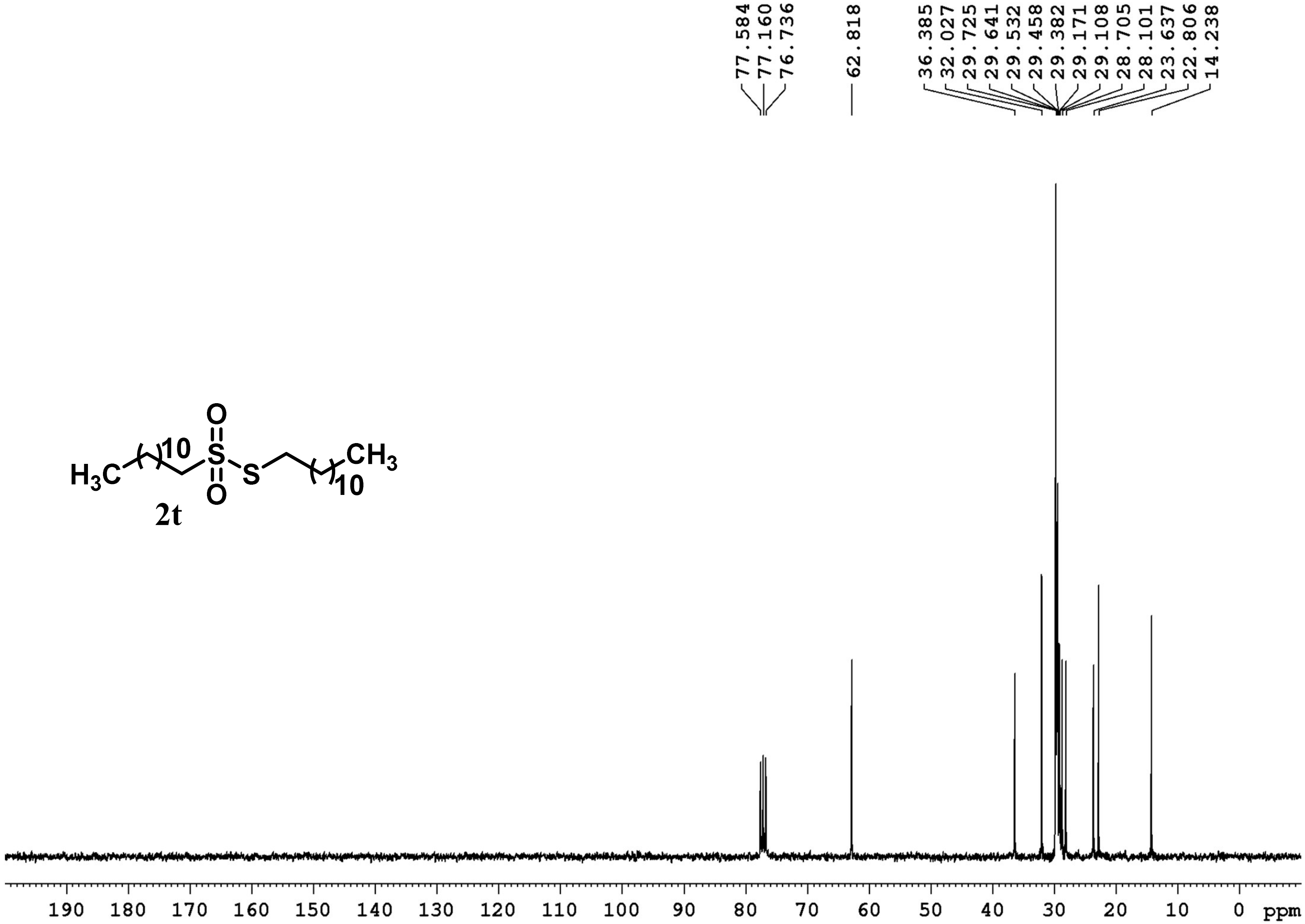

^{13}C {^{1}H} NMR of compound **2t** (75 MHz, $CDCl_3$)

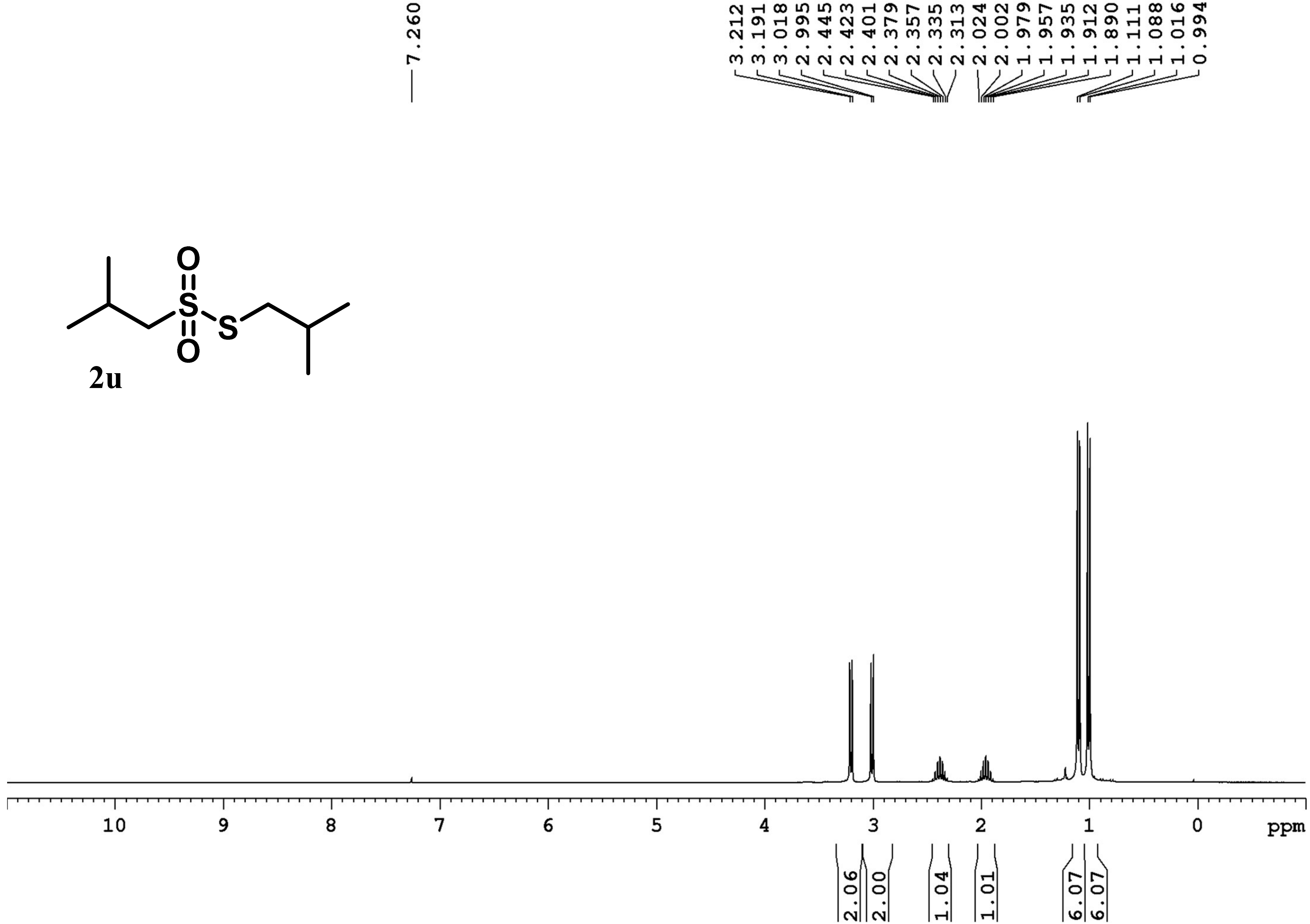

^{1}H NMR of compound **2u** (300 MHz, $CDCl_3$)

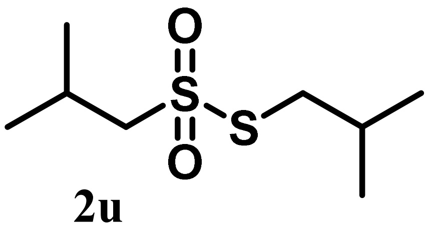

2u

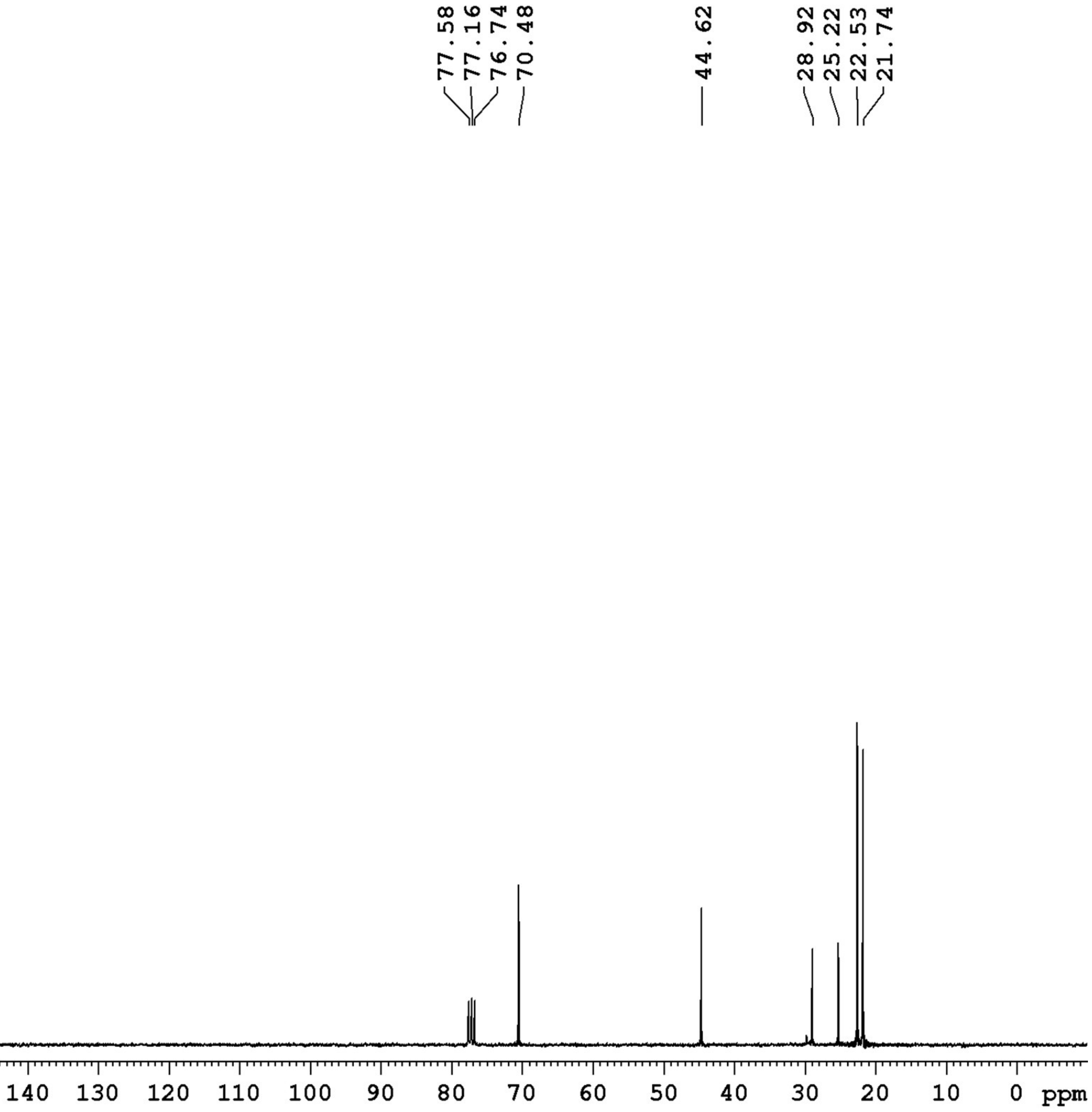

$^{13}C\ \{^{1}H\}$ NMR of compound **2u** (75 MHz, $CDCl_3$)

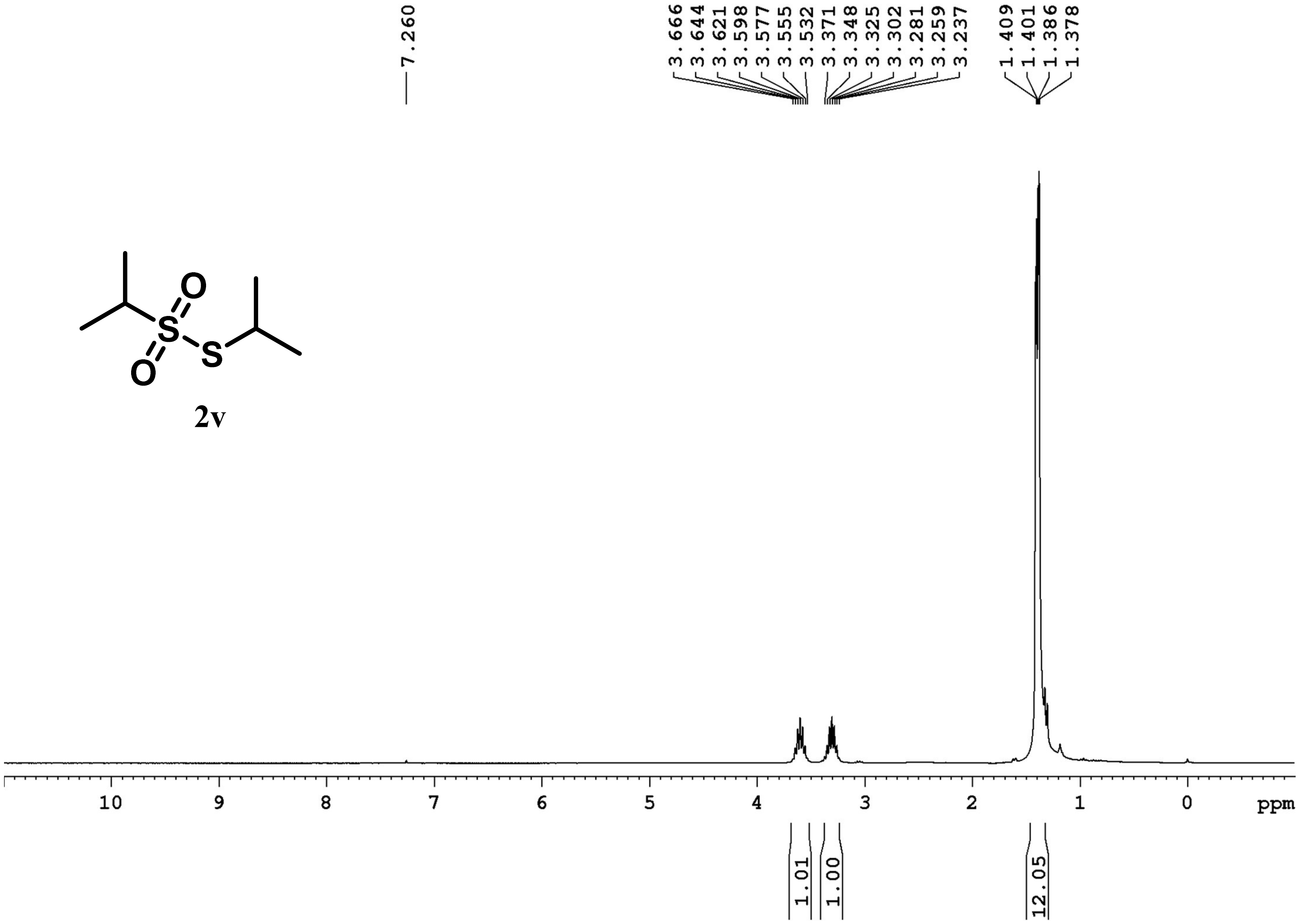

^{1}H NMR of compound **2v** (300 MHz, $CDCl_3$)

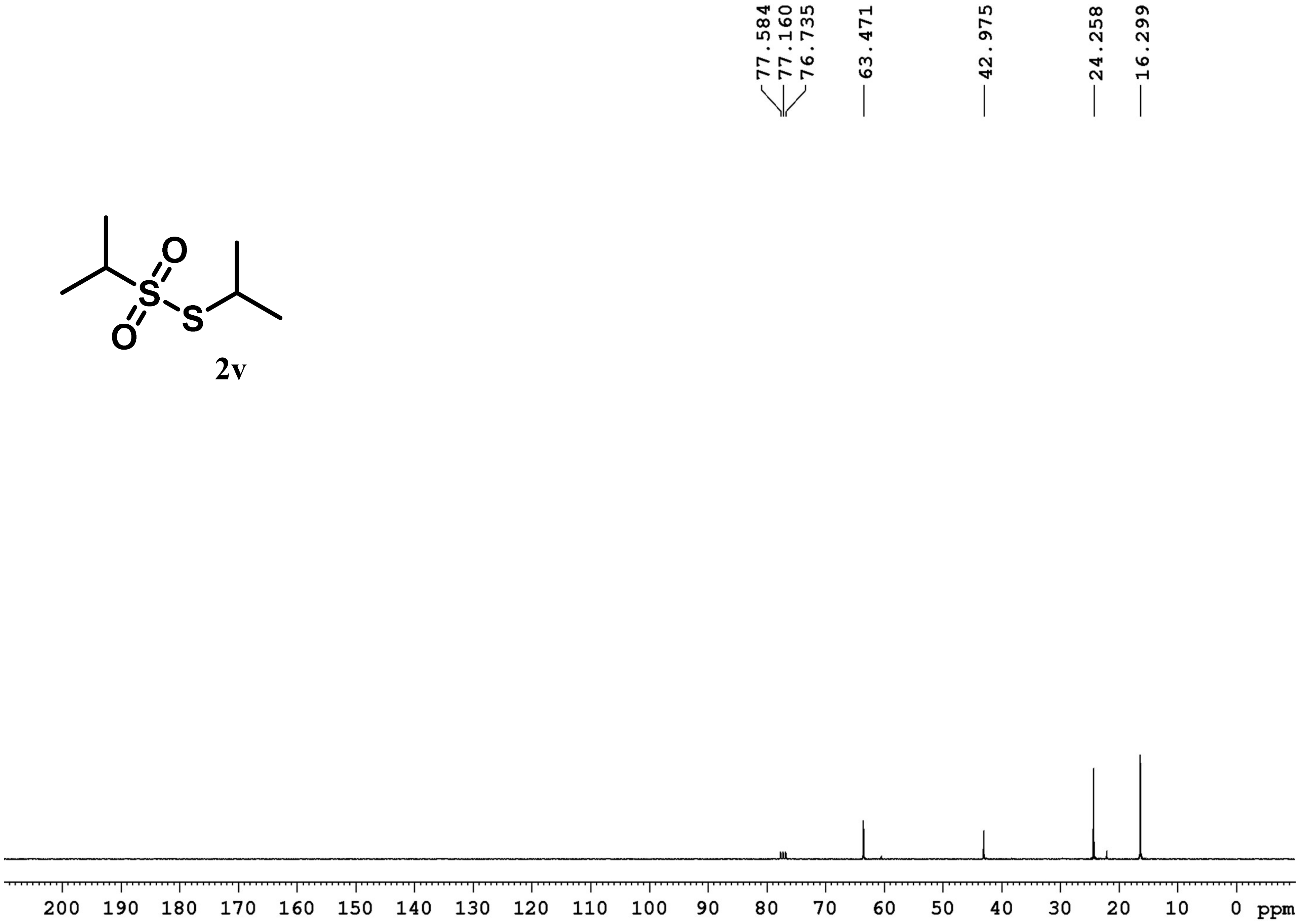

^{13}C {^{1}H} NMR of compound **2v** (75 MHz, $CDCl_3$)

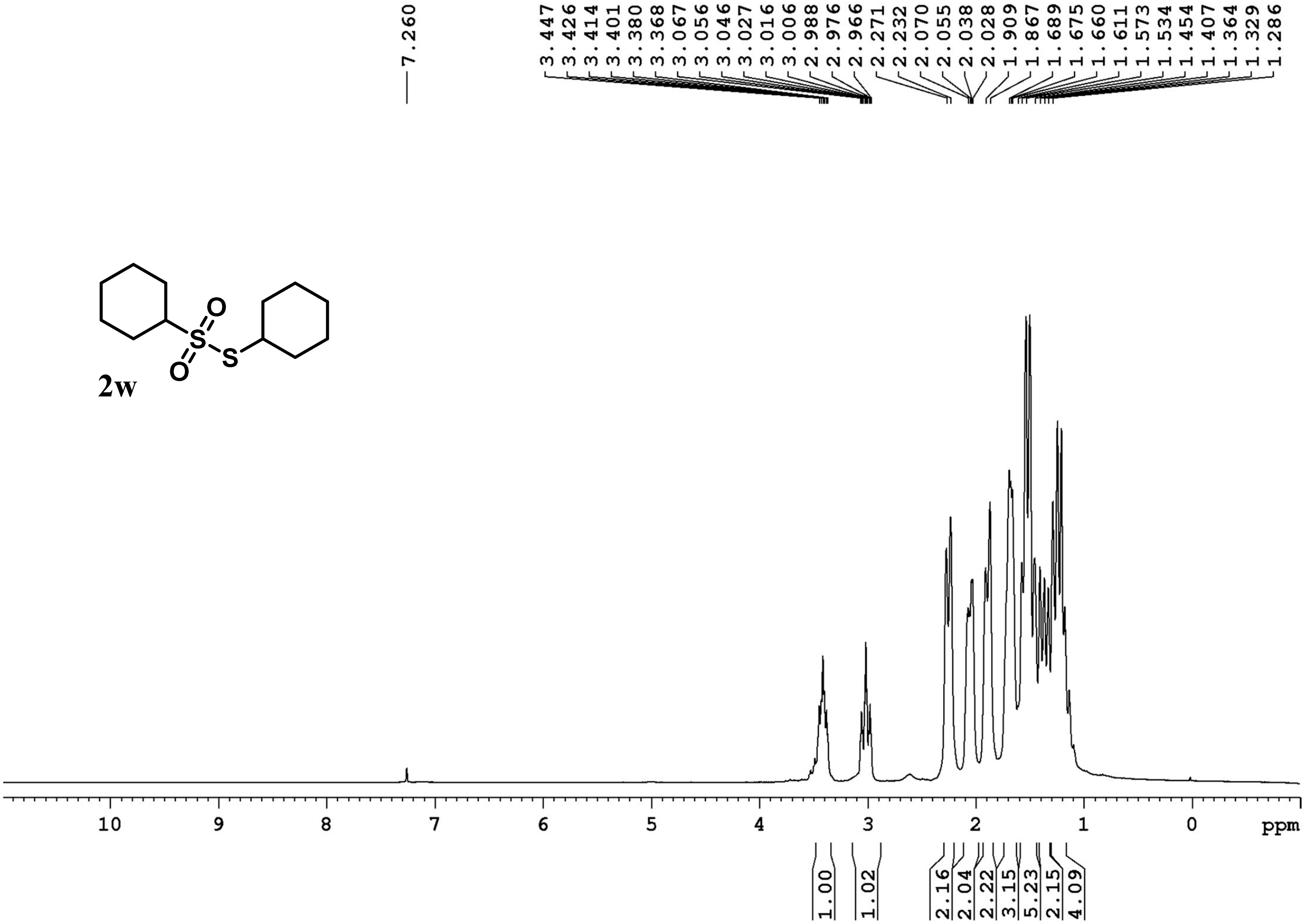

^{1}H NMR of compound **2w** (300 MHz, $CDCl_3$)

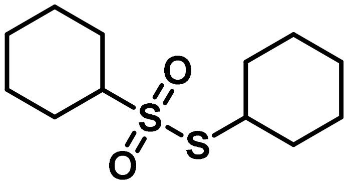

2w

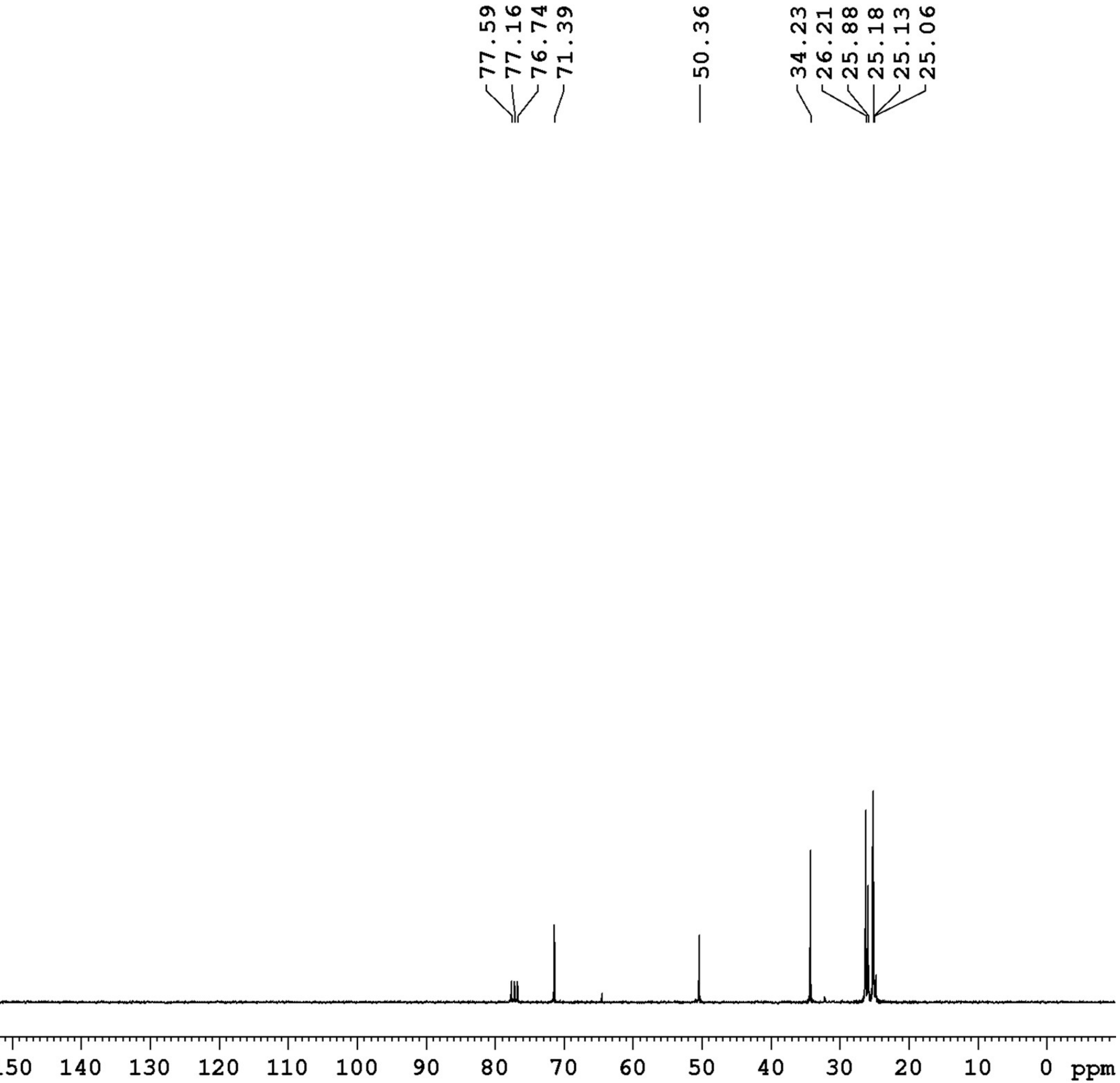

^{13}C {^{1}H} NMR of compound **2w** (75 MHz, $CDCl_3$)

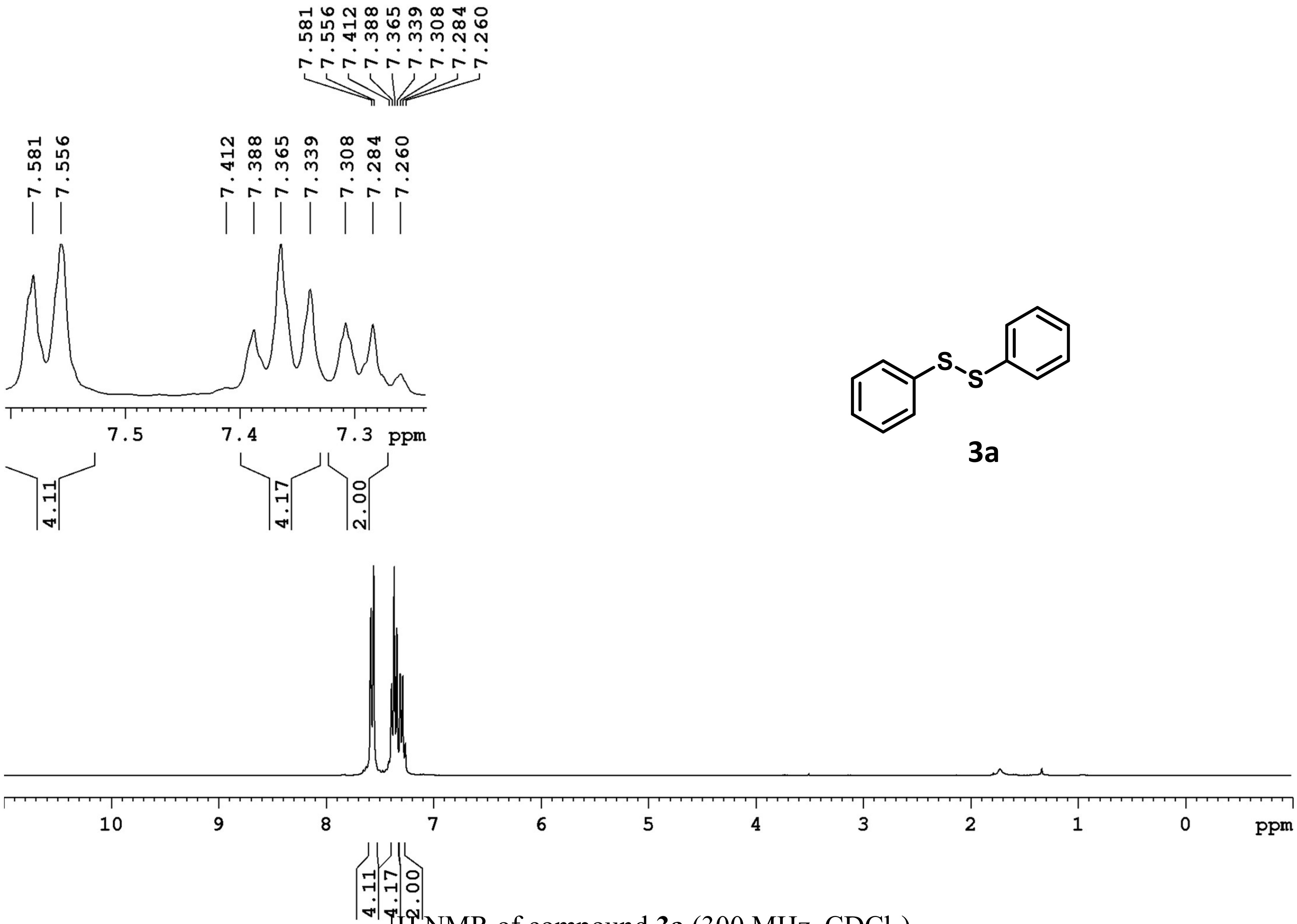

3a

^{1}H NMR of compound **3a** (300 MHz, $CDCl_3$)

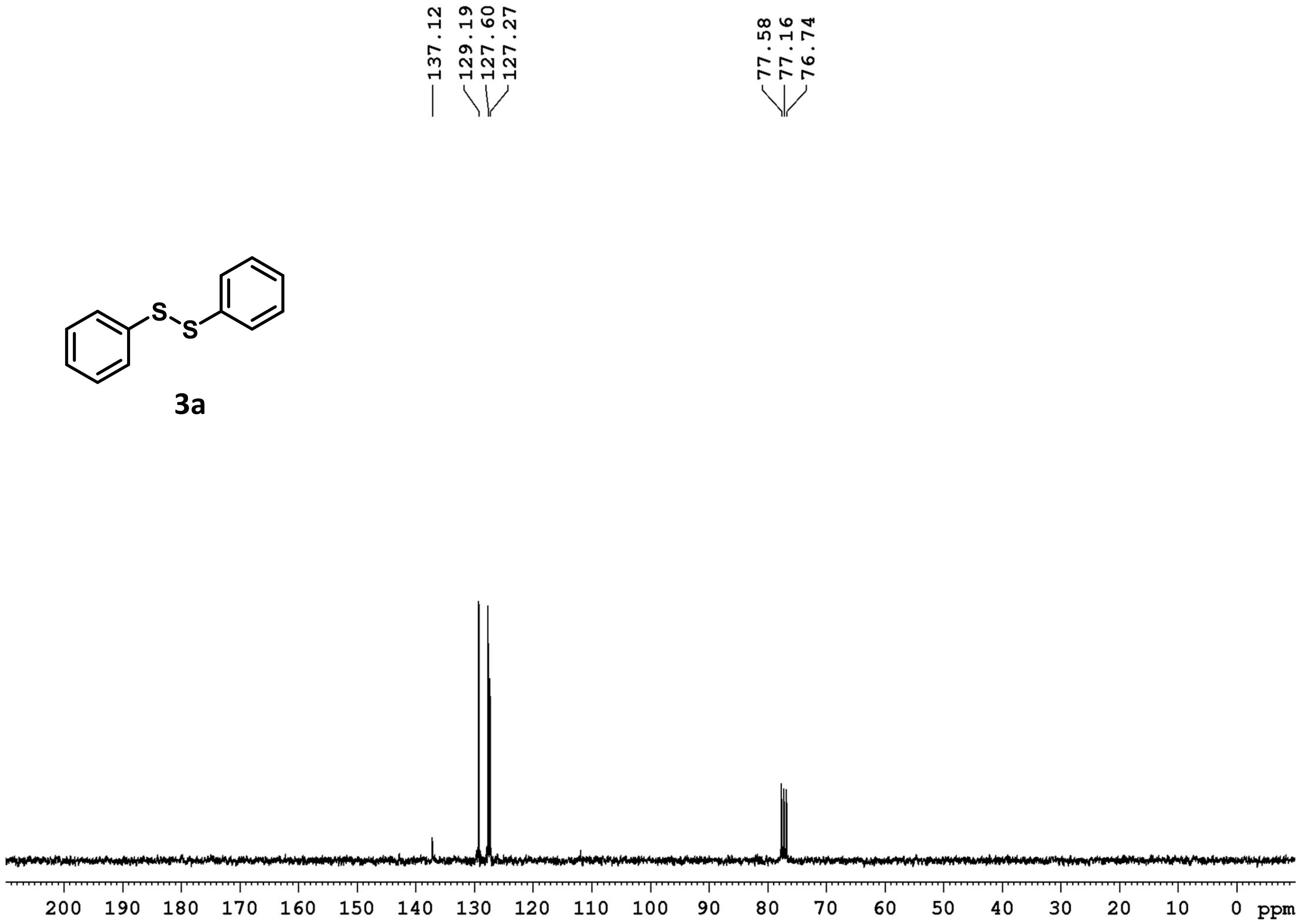

^{13}C {^{1}H} NMR of compound **3a** (75 MHz, $CDCl_3$)

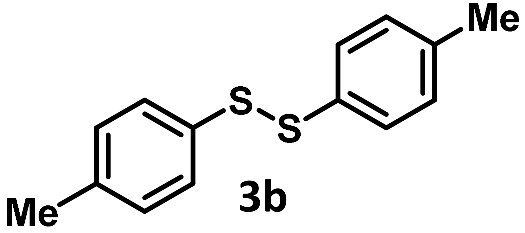

3b

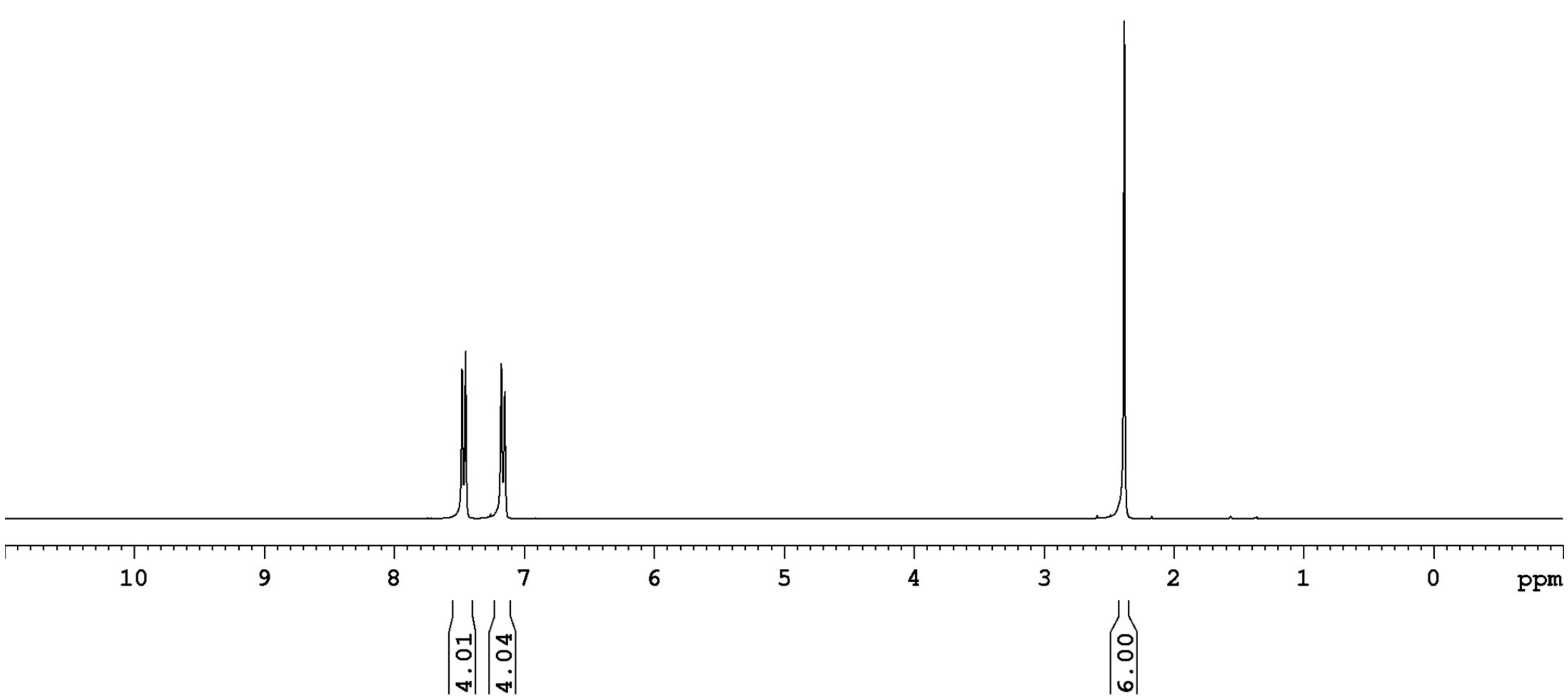

^{1}H NMR of compound **3b** (300 MHz, $CDCl_3$)

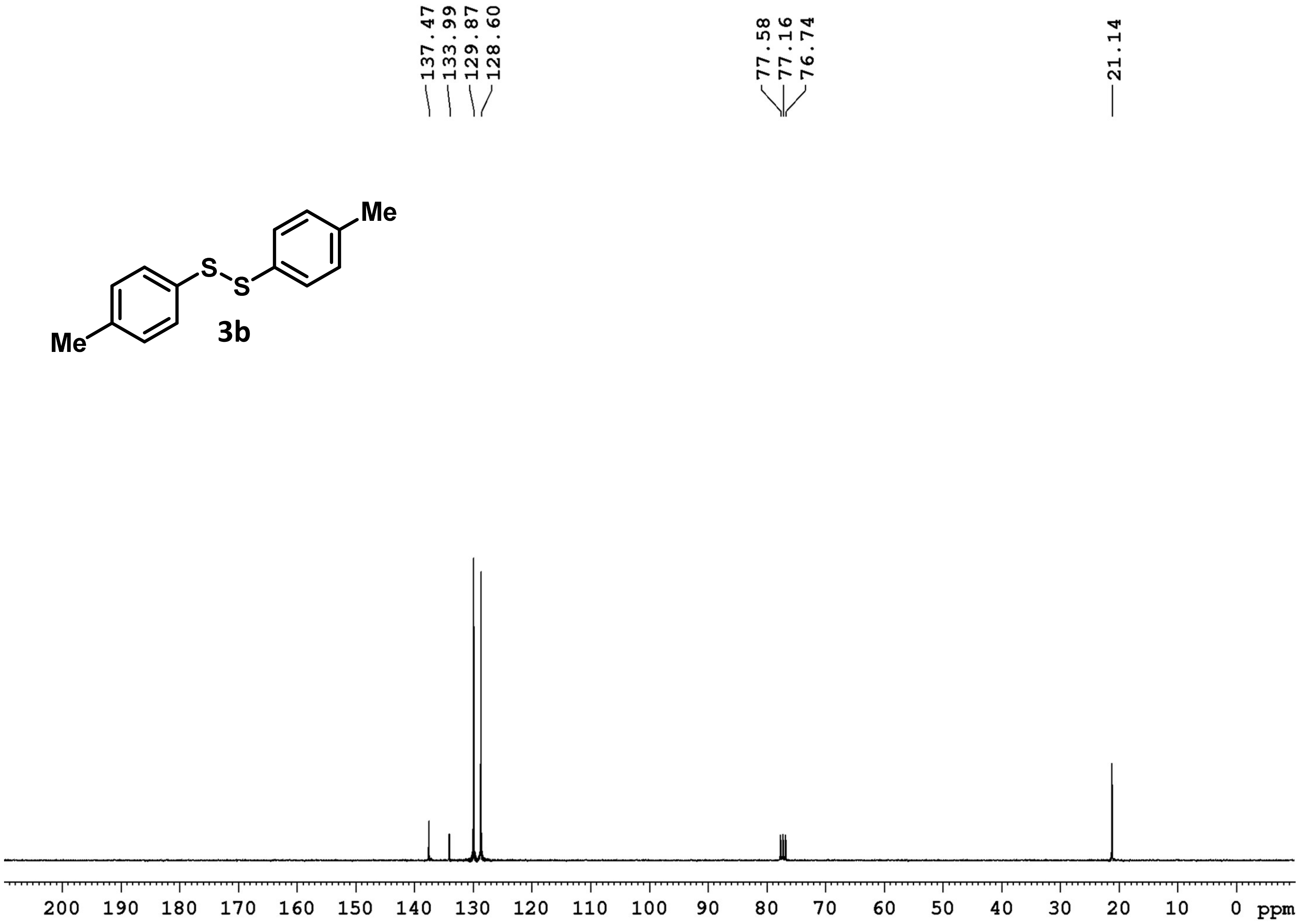

^{13}C {^{1}H} NMR of compound **3b** (75 MHz, $CDCl_3$)

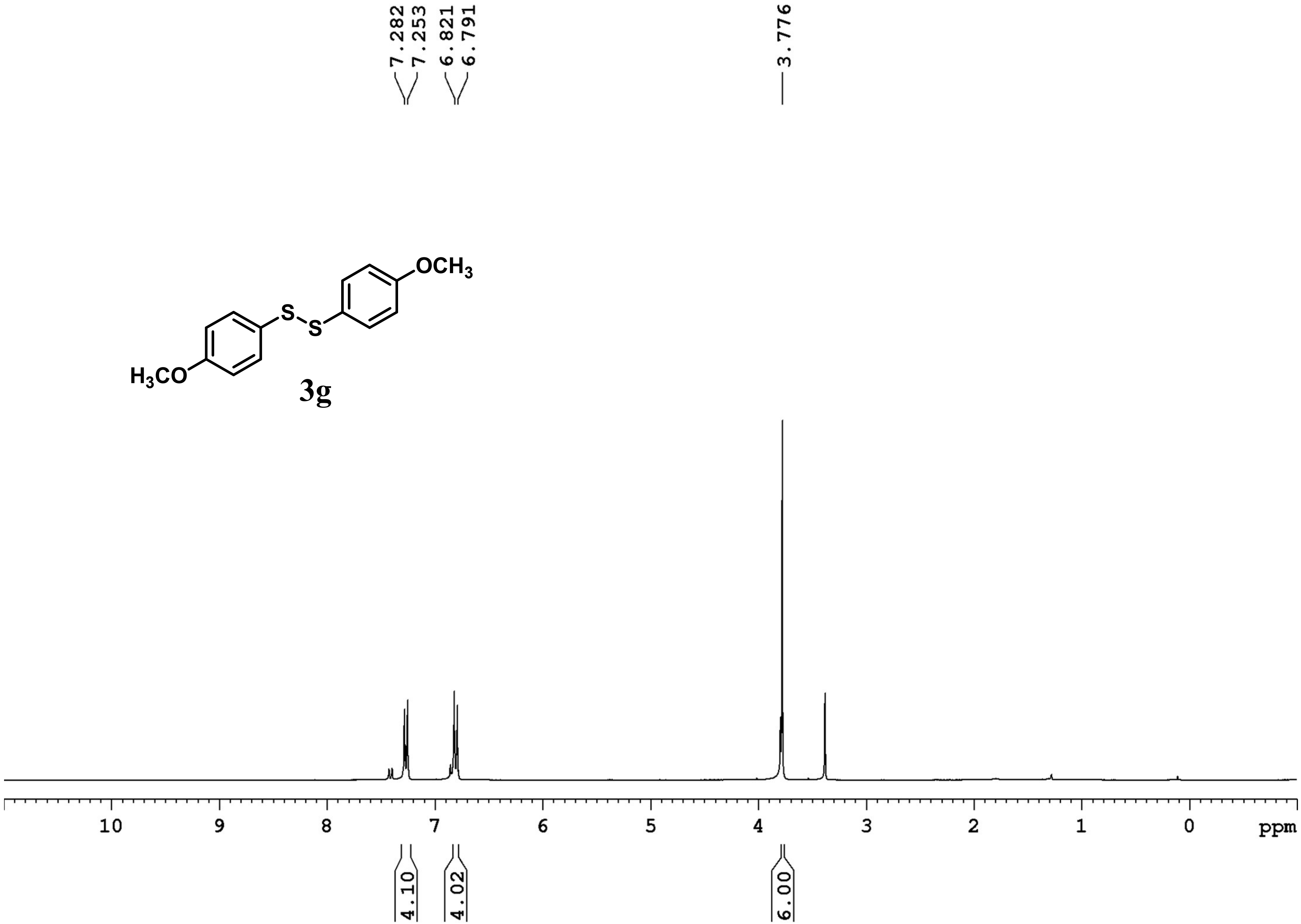

^{1}H NMR of compound **3g** (300 MHz, $CDCl_3$)

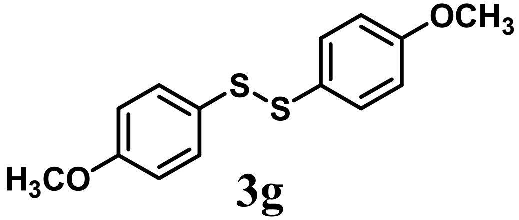

3g

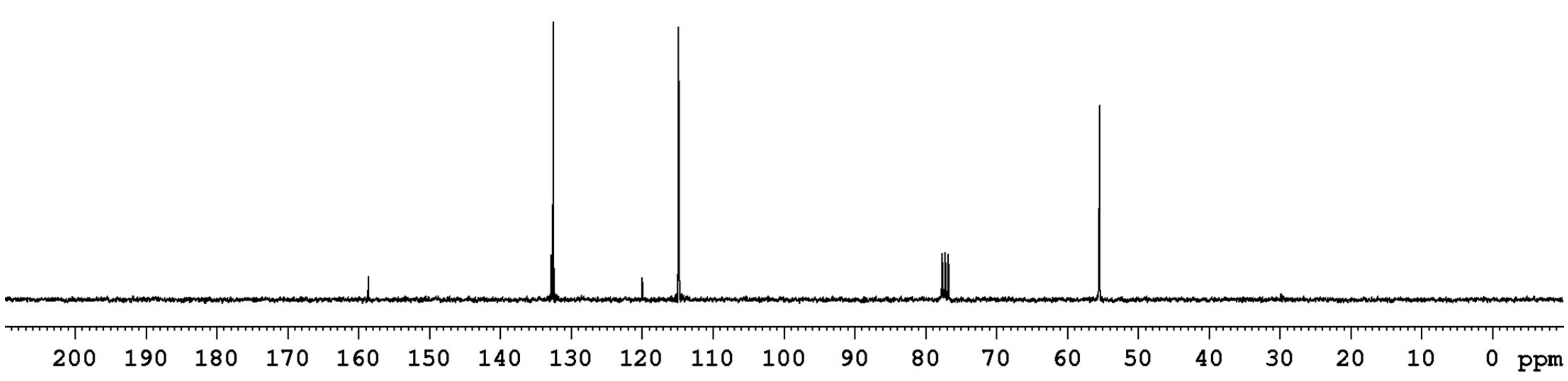

^{13}C {^{1}H} NMR of compound **3g** (75 MHz, $CDCl_3$)

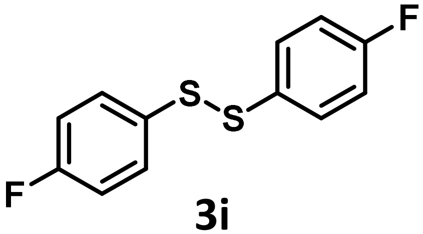

3i

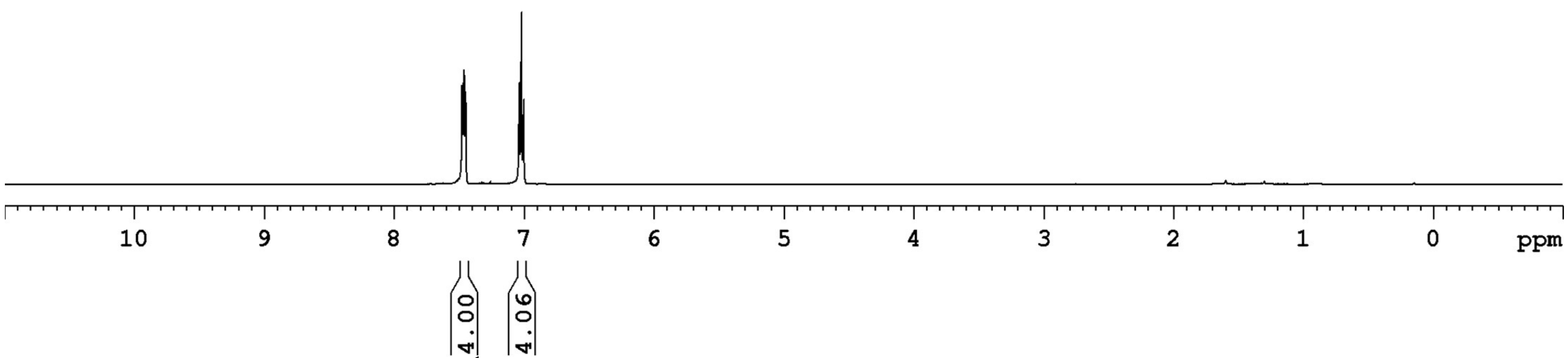

^{1}H NMR of compound **3i** (300 MHz, $CDCl_3$)

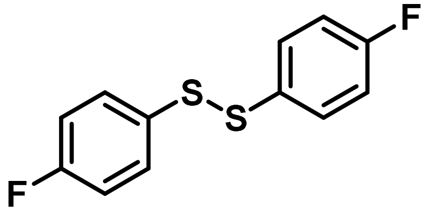

3i

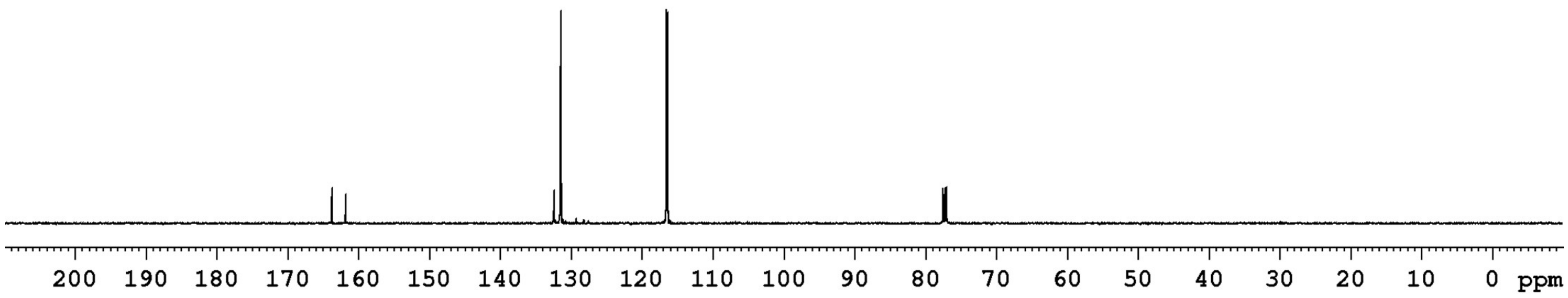

^{13}C {^{1}H} NMR of compound **3i** (75 MHz, $CDCl_3$)

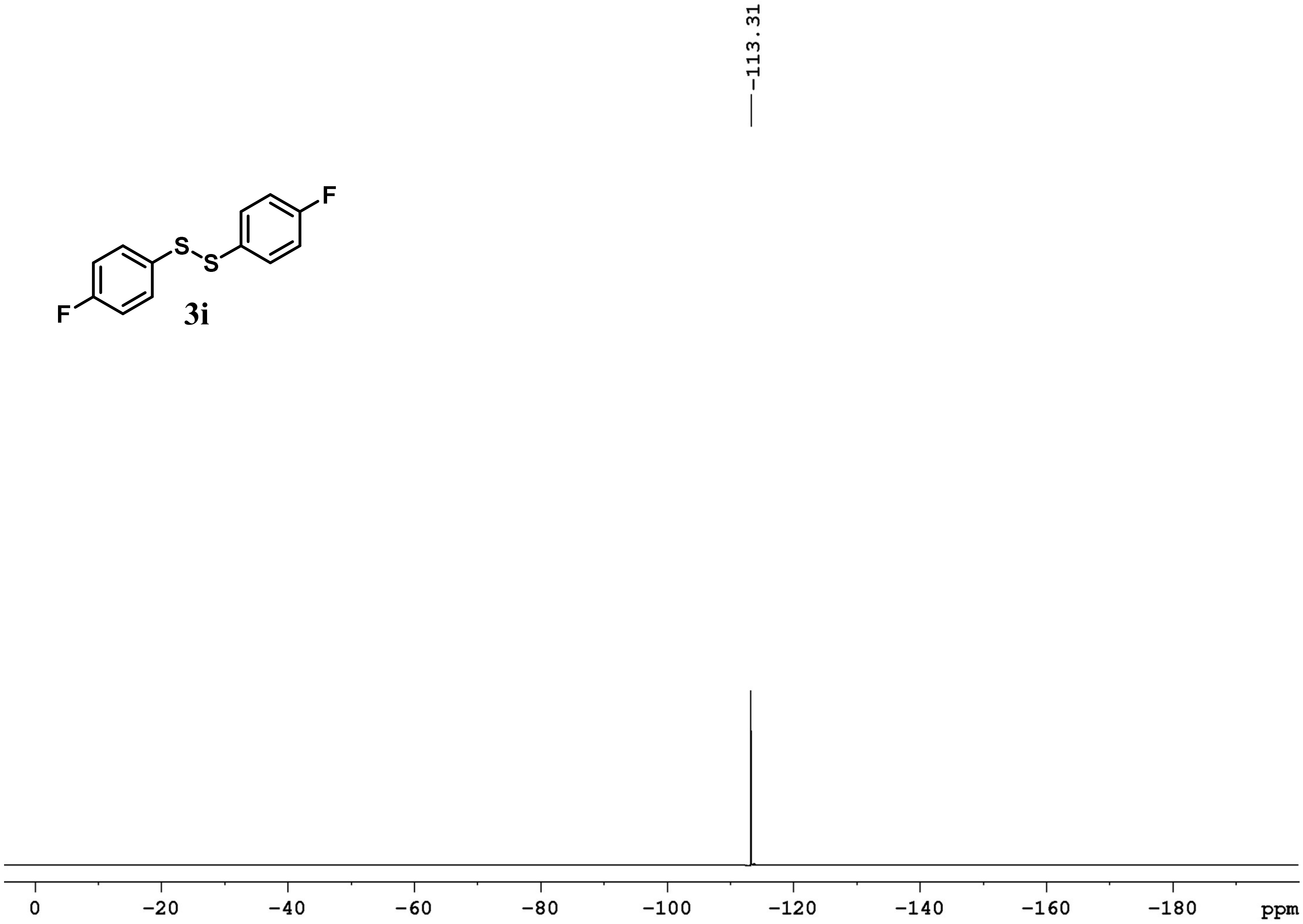

^{19}F {^{1}H} NMR of compound **3i** (470 MHz, $CDCl_3$)

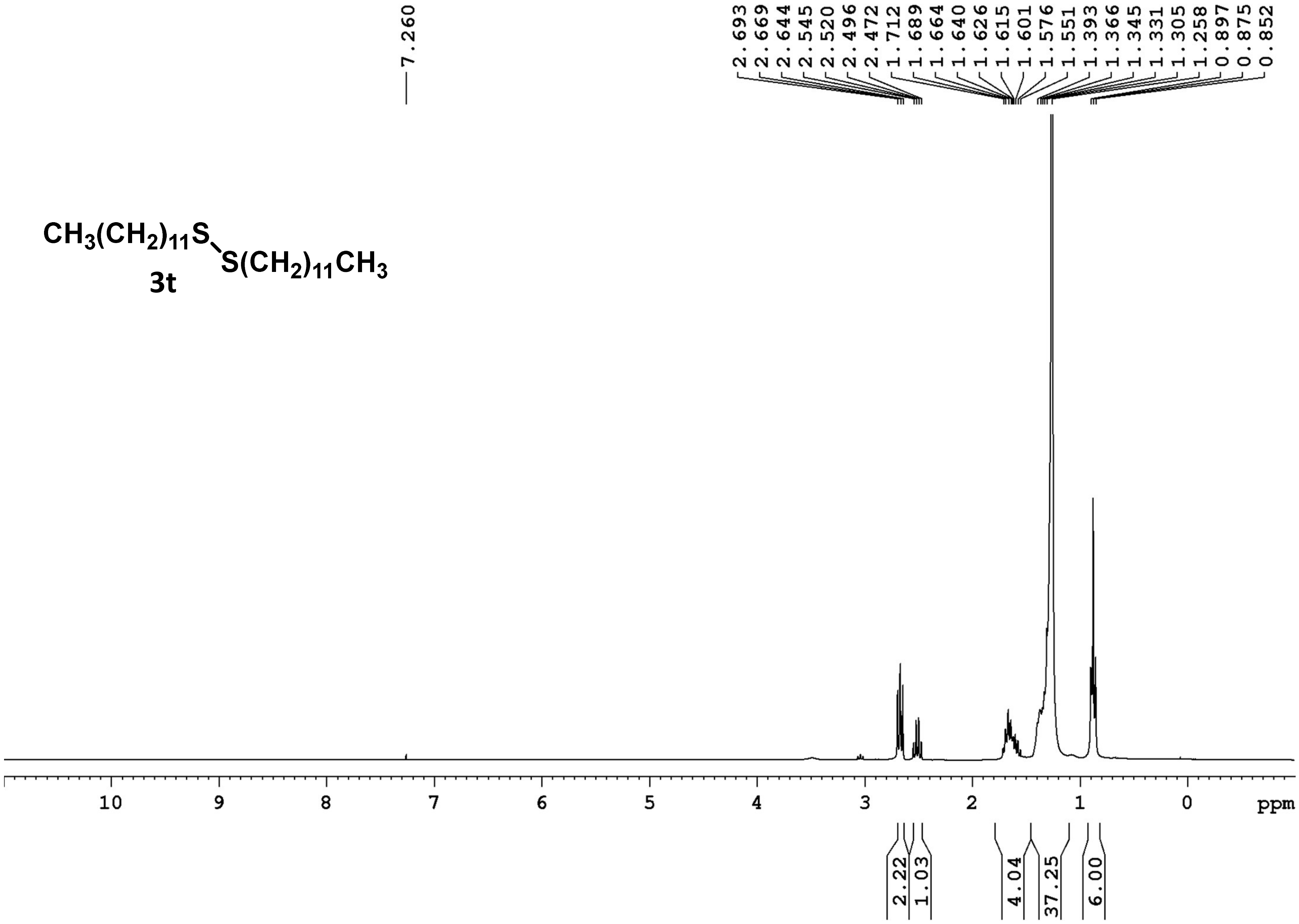

^{1}H NMR of compound **3t** (300 MHz, $CDCl_3$)

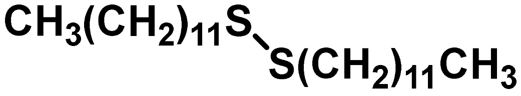

3t

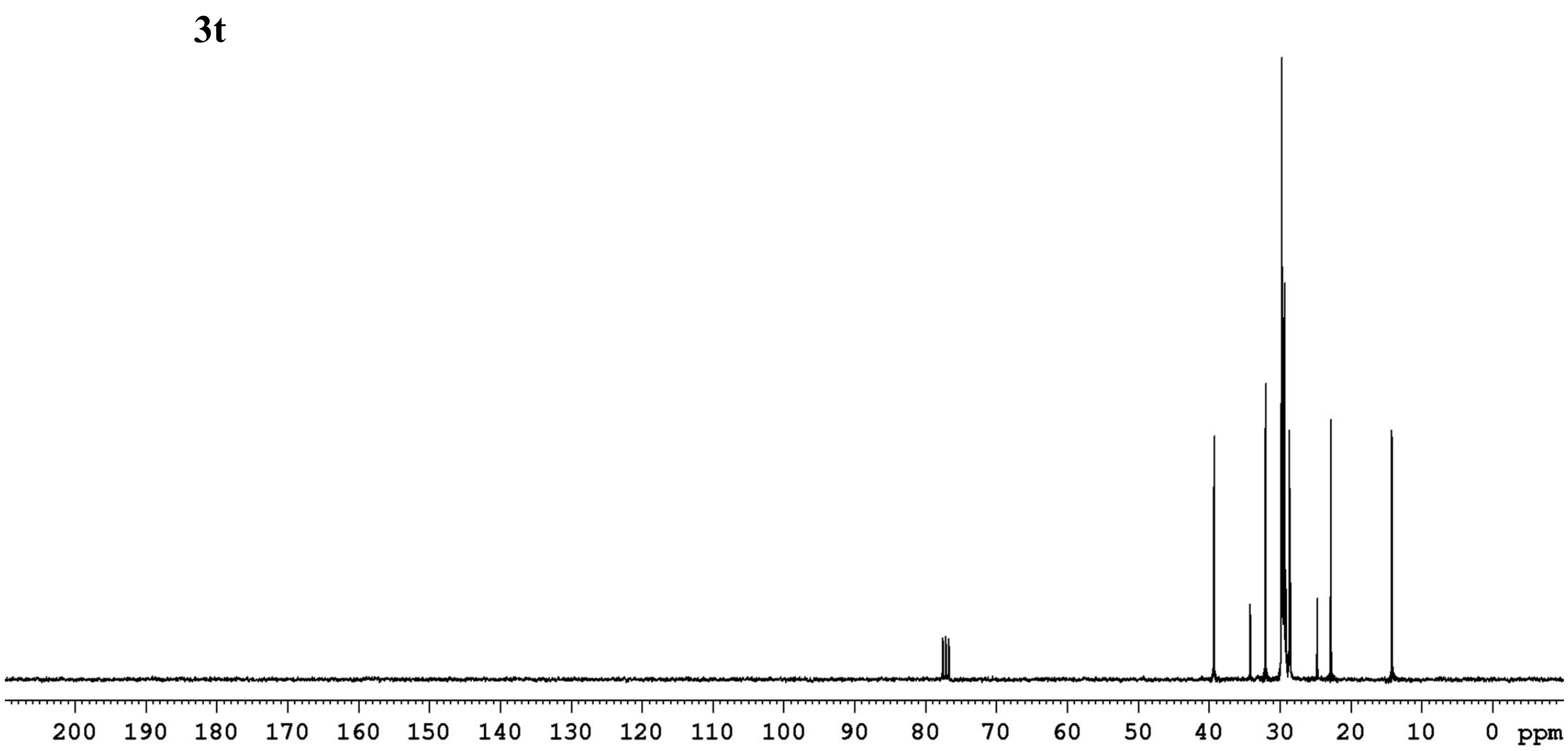

^{13}C {^{1}H} NMR of compound **3t** (75 MHz, $CDCl_3$)

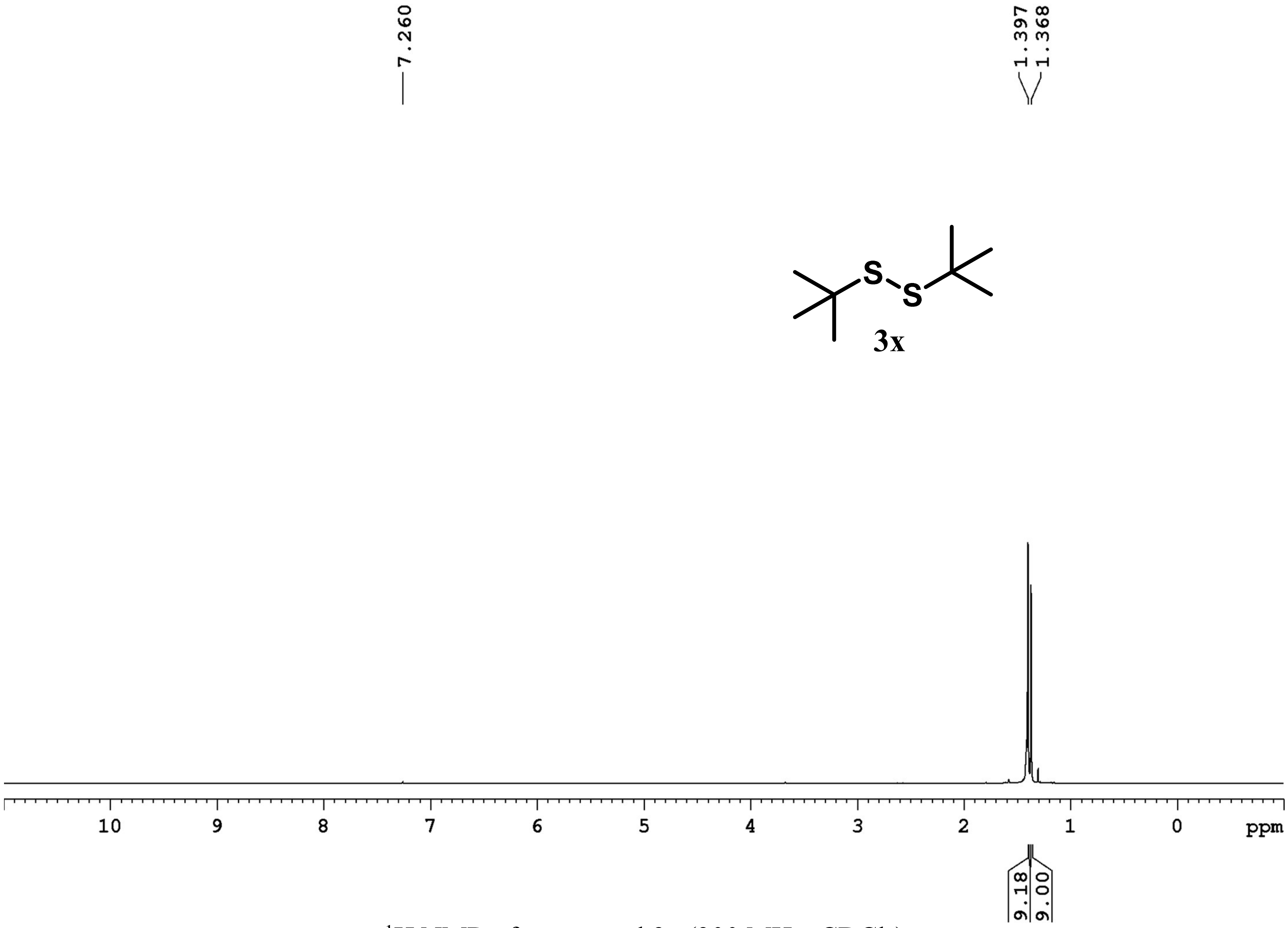

^{1}H NMR of compound **3x** (300 MHz, $CDCl_3$)

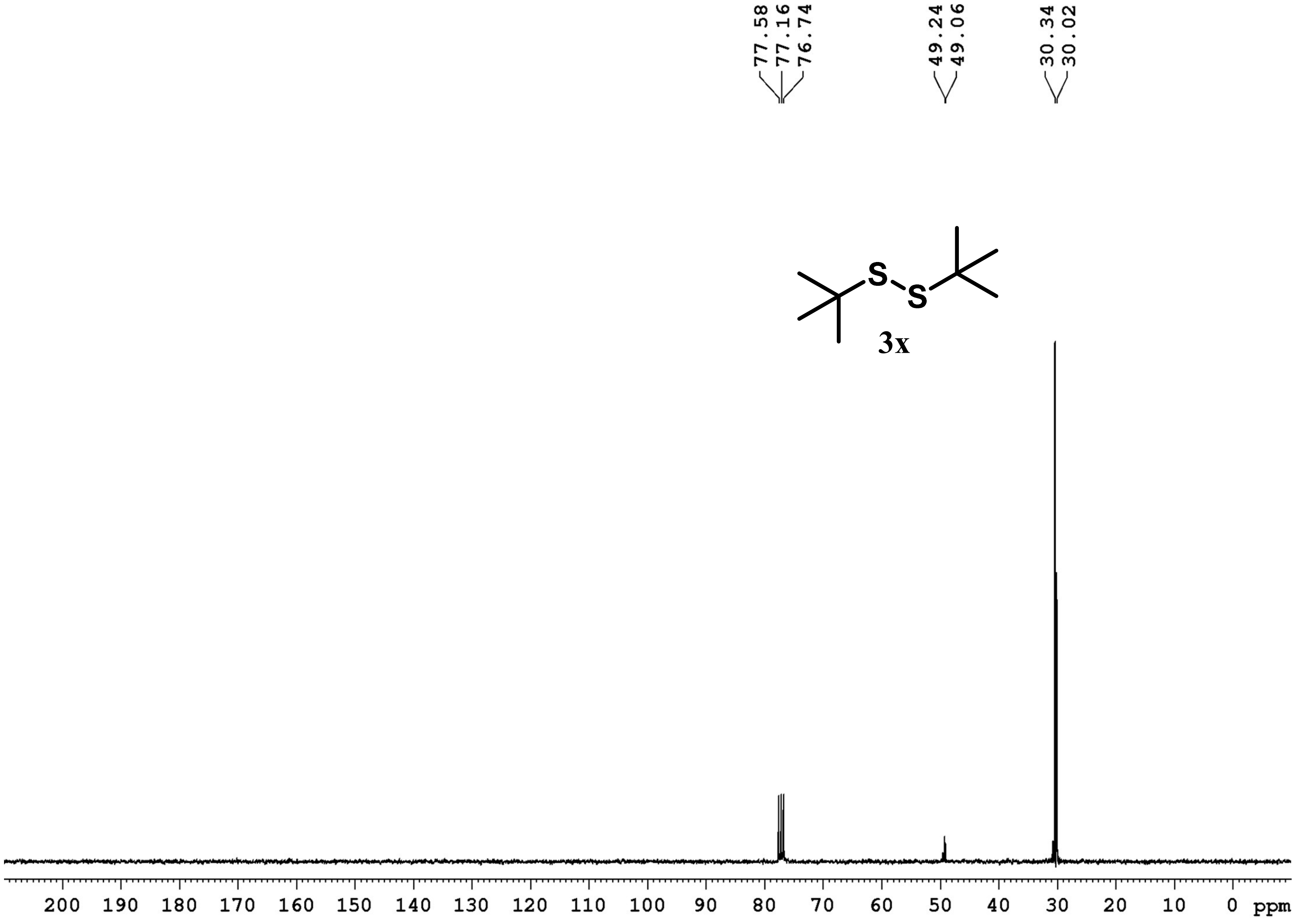

^{13}C {^{1}H} NMR of compound **3x** (75 MHz, $CDCl_3$)

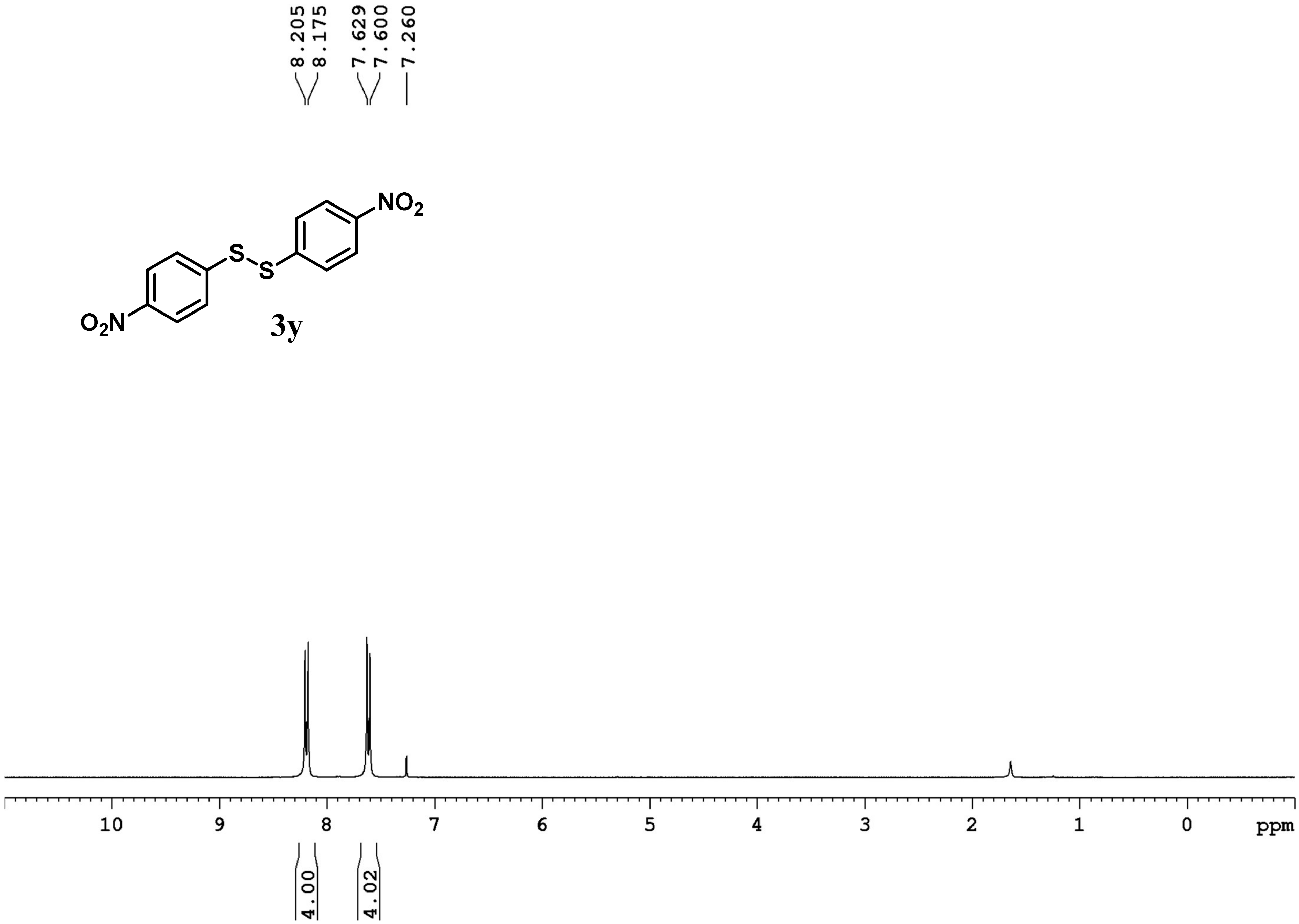

^{1}H NMR of compound **3y** (300 MHz, $CDCl_3$)

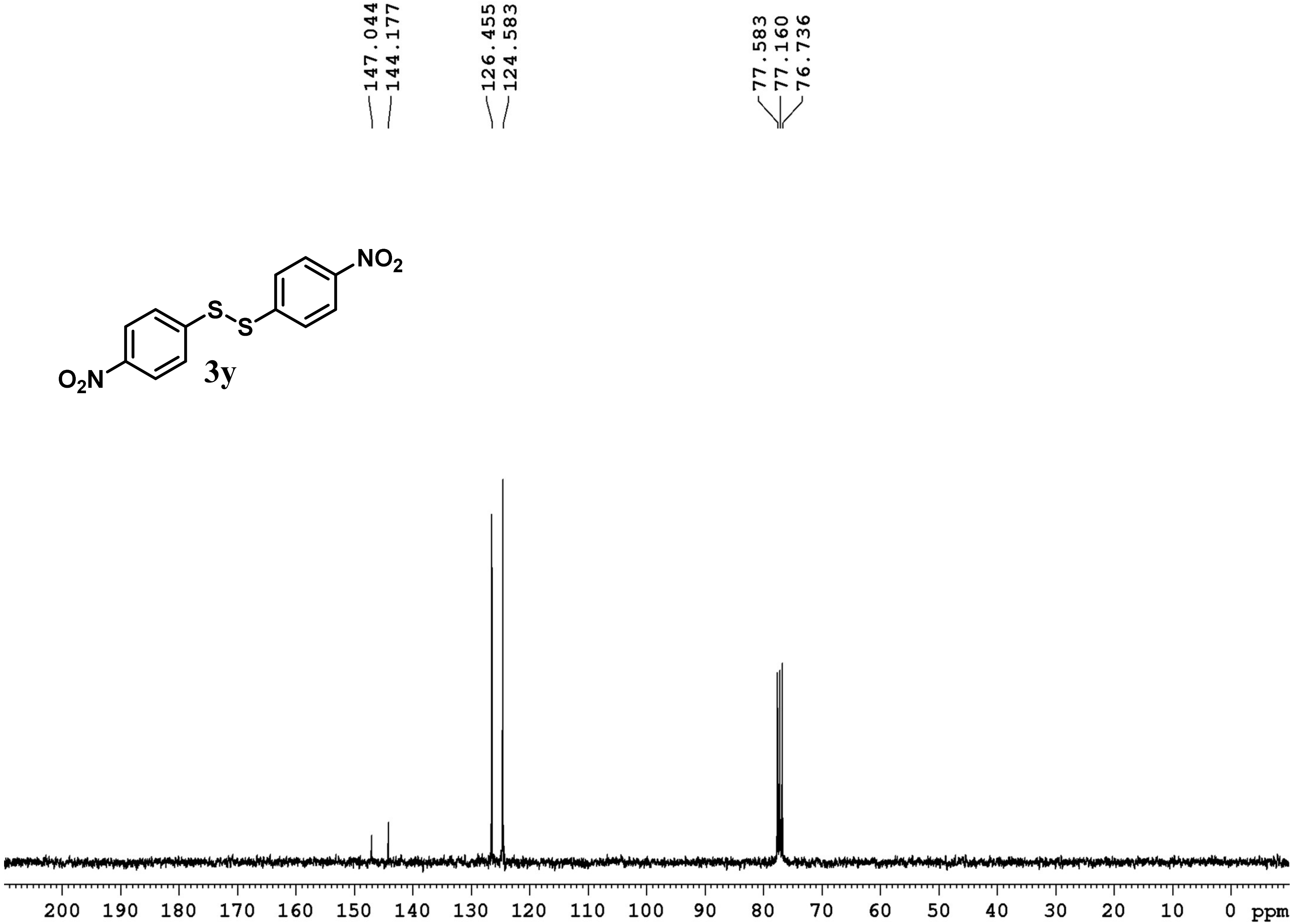

3y

^{13}C {^{1}H} NMR of compound **3y** (75 MHz, $CDCl_3$)

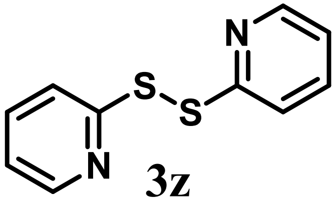

3z

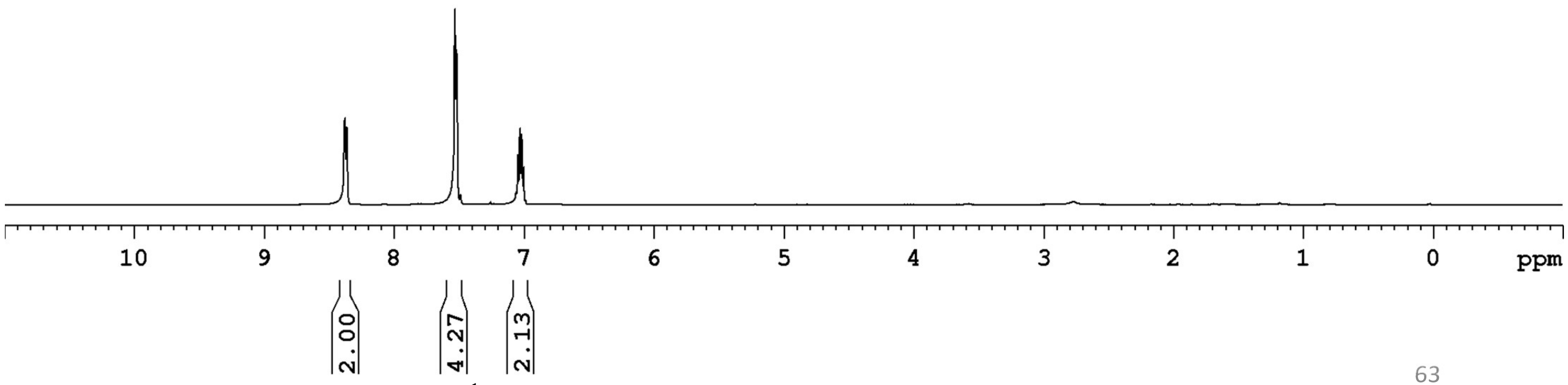

^{1}H NMR of compound **3z** (300 MHz, $CDCl_3$)

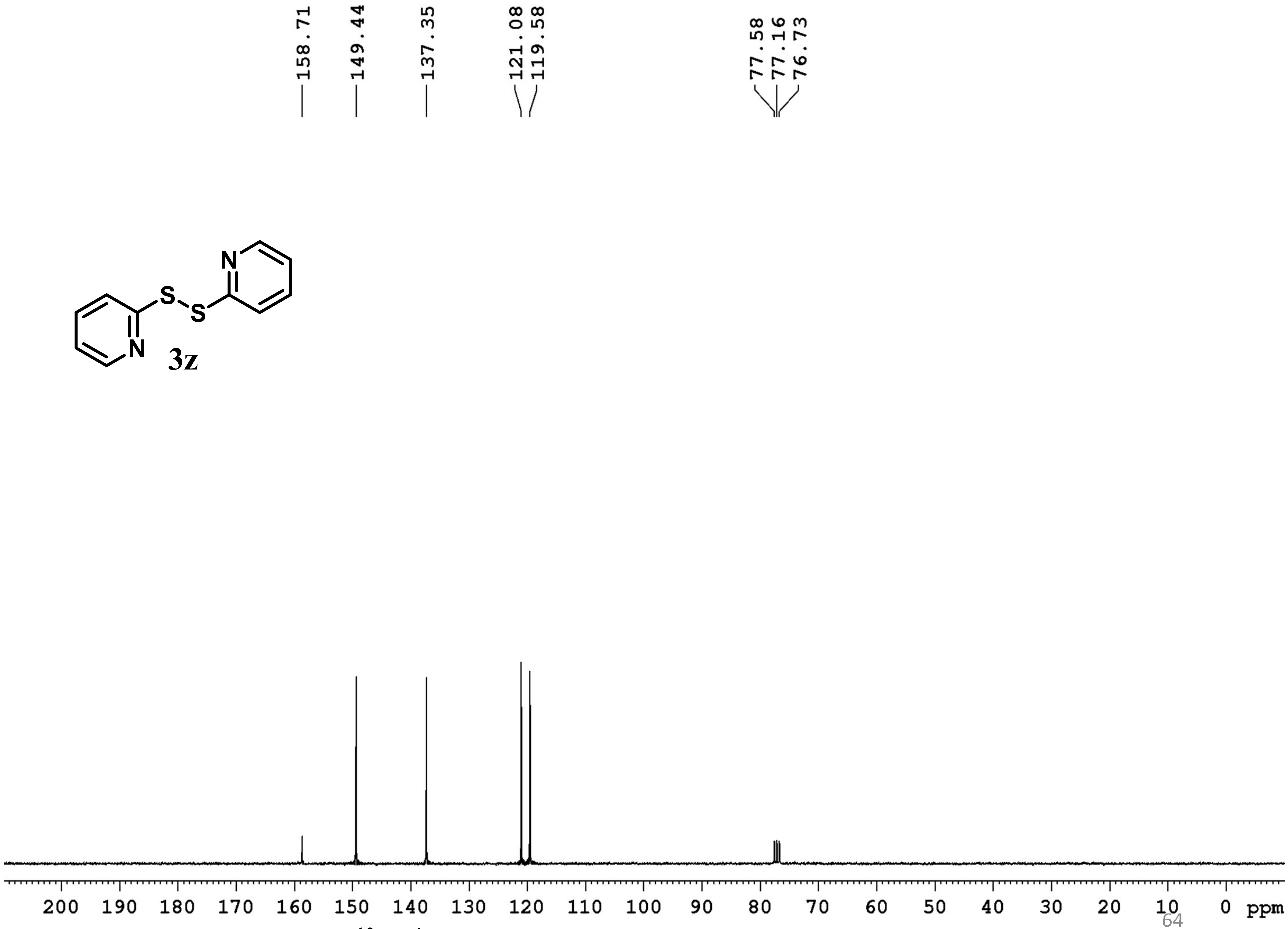

64

^{13}C {^{1}H} NMR of compound **3z** (75 MHz, $CDCl_3$)

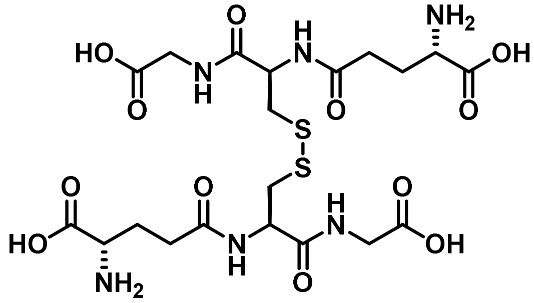

5

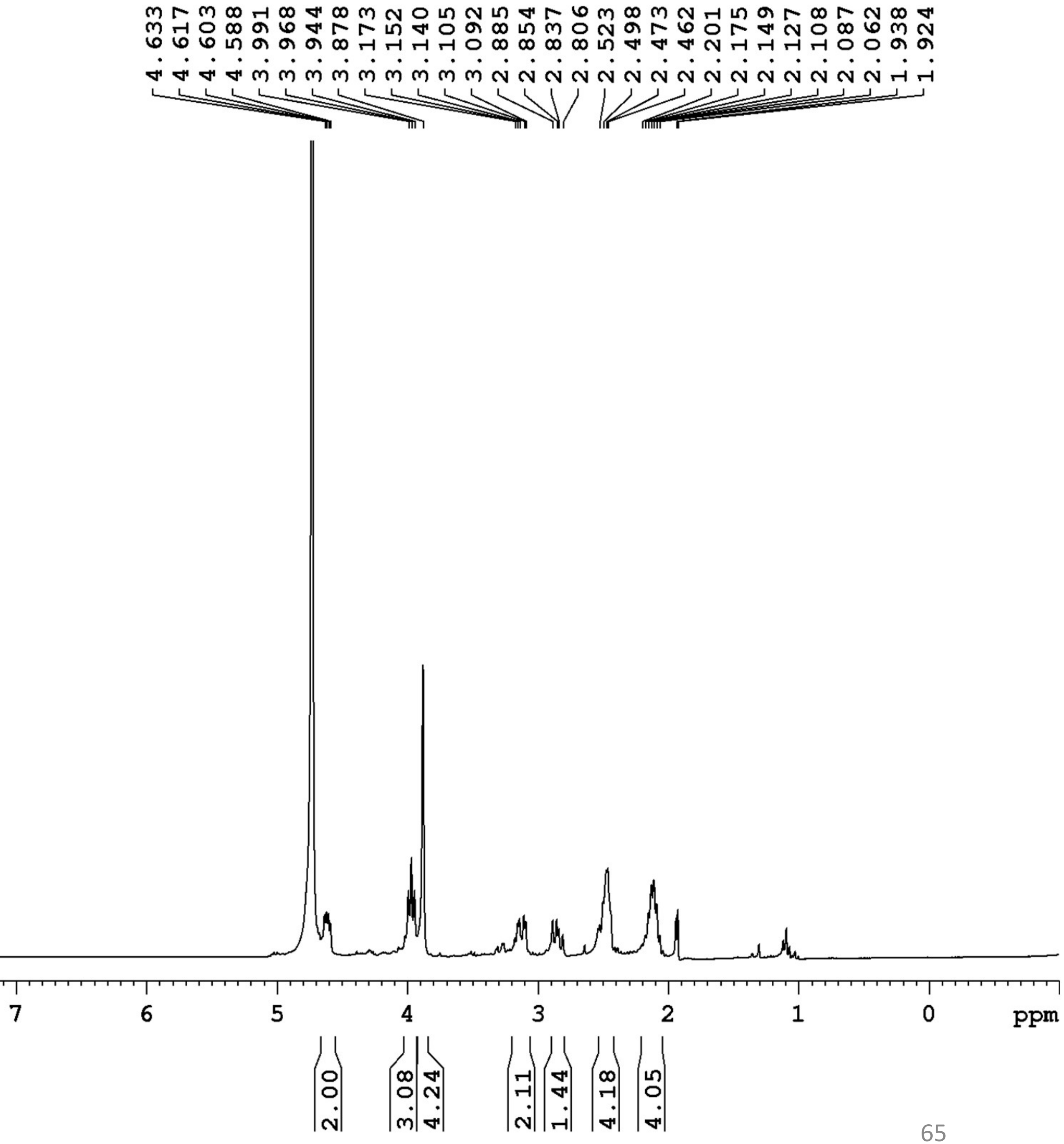

^{1}H NMR of compound **5** (300 MHz, D_2O)

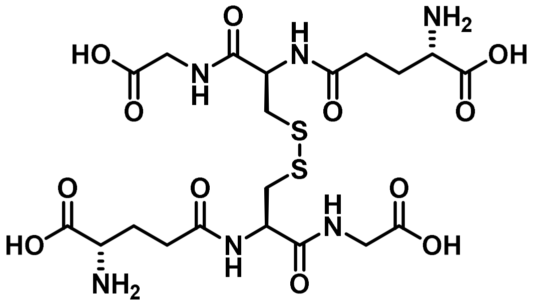

5

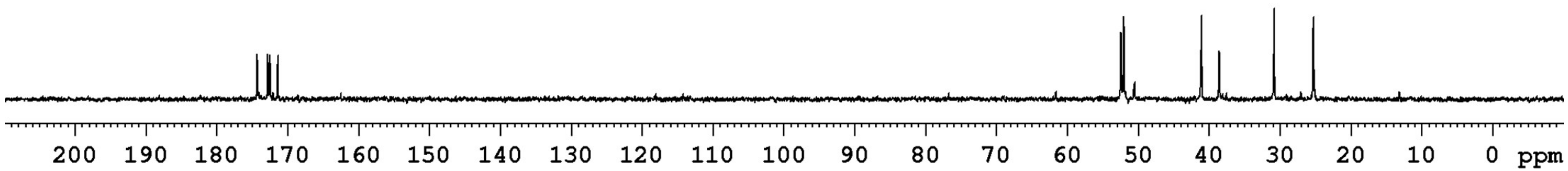

^{13}C {^{1}H} NMR of compound **5** (75 MHz, D_2O)
