# Supplementary figures and images for: A Stepwise, Nitrosonium-Catalyzed Aerobic Oxidation of Thiols to Disulfides and Thiosulfonates
Source: J Org Chem. 2026 Mar 20;91(13):4755–66. doi: 10.1021/acs.joc.6c00062 (PMC13054865; doi:10.1021/acs.joc.6c00062)

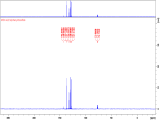

Supplement: Supplementary file 2 [file jo6c00062_si_002.zip › Thiosulfonate FID Files 23-12-2025/2a/13C/pdata/1/thumb.png]

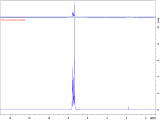

Supplement: Supplementary file 2 [file jo6c00062_si_002.zip › Thiosulfonate FID Files 23-12-2025/2a/1H/pdata/1/thumb.png]

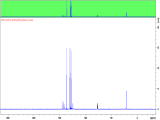

Supplement: Supplementary file 2 [file jo6c00062_si_002.zip › Thiosulfonate FID Files 23-12-2025/2b/13C/pdata/1/thumb.png]

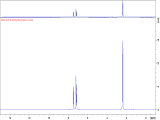

Supplement: Supplementary file 2 [file jo6c00062_si_002.zip › Thiosulfonate FID Files 23-12-2025/2b/1H/pdata/1/thumb.png]

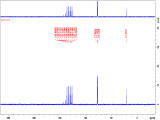

Supplement: Supplementary file 2 [file jo6c00062_si_002.zip › Thiosulfonate FID Files 23-12-2025/2c/13C/pdata/1/thumb.png]

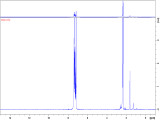

Supplement: Supplementary file 2 [file jo6c00062_si_002.zip › Thiosulfonate FID Files 23-12-2025/2c/1H/pdata/1/thumb.png]

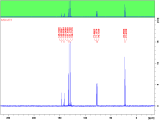

Supplement: Supplementary file 2 [file jo6c00062_si_002.zip › Thiosulfonate FID Files 23-12-2025/2d/13C/pdata/1/thumb.png]

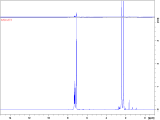

Supplement: Supplementary file 2 [file jo6c00062_si_002.zip › Thiosulfonate FID Files 23-12-2025/2d/1H/pdata/1/thumb.png]
